# TFAP2 paralogs regulate midfacial development in part through a conserved ALX genetic pathway

**DOI:** 10.1242/dev.202095

**Published:** 2024-01-02

**Authors:** Timothy T. Nguyen, Jennyfer M. Mitchell, Michaela D. Kiel, Colin P. Kenny, Hong Li, Kenneth L. Jones, Robert A. Cornell, Trevor J. Williams, James T. Nichols, Eric Van Otterloo

**Affiliations:** ^1^ Iowa Institute for Oral Health Research, College of Dentistry and Dental Clinics, University of Iowa, Iowa City, IA 52242, USA; ^2^ Department of Periodontics, College of Dentistry and Dental Clinics, University of Iowa, Iowa City, IA 52242, USA; ^3^ Department of Anatomy and Cell Biology, Carver College of Medicine, University of Iowa, Iowa City, IA 52242, USA; ^4^ Interdisciplinary Graduate Program in Genetics, University of Iowa, Iowa City, IA 52242, USA; ^5^ Department of Craniofacial Biology, University of Colorado Anschutz Medical Campus, Aurora, CO 80045, USA; ^6^ Department of Surgery, Carver College of Medicine, University of Iowa, Iowa City, IA 52242, USA; ^7^ Department of Pediatrics, University of Colorado Anschutz Medical Campus, Children's Hospital Colorado, Aurora, CO 80045, USA; ^8^ Department of Oral Health Sciences, University of Washington, School of Dentistry, Seattle, WA 98195, USA; ^9^ Department of Cell and Developmental Biology, University of Colorado Anschutz Medical Campus, Aurora, CO 80045, USA; ^10^ Craniofacial Anomalies Research Center, University of Iowa, Iowa City, IA 52242, USA

**Keywords:** TFAP2, Branchio-oculo-facial syndrome, Char syndrome, ALX, Frontonasal dysplasia, Neural crest

## Abstract

Cranial neural crest development is governed by positional gene regulatory networks (GRNs). Fine-tuning of the GRN components underlies facial shape variation, yet how those networks in the midface are connected and activated remain poorly understood. Here, we show that concerted inactivation of *Tfap2a* and *Tfap2b* in the murine neural crest, even during the late migratory phase, results in a midfacial cleft and skeletal abnormalities. Bulk and single-cell RNA-seq profiling reveal that loss of both TFAP2 family members dysregulates numerous midface GRN components involved in midface morphogenesis, patterning and differentiation. Notably, *Alx1*, *Alx3* and *Alx4* (ALX) transcript levels are reduced, whereas ChIP-seq analyses suggest TFAP2 family members directly and positively regulate ALX gene expression. *Tfap2a*, *Tfap2b* and ALX co-expression in midfacial neural crest cells of both mouse and zebrafish implies conservation of this regulatory axis across vertebrates. Consistent with this notion, *tfap2a* zebrafish mutants present with abnormal *alx3* expression patterns, Tfap2a binds ALX loci and *tfap2a-alx3* genetic interactions are observed. Together, these data demonstrate TFAP2 paralogs regulate vertebrate midfacial development in part by activating expression of ALX transcription factor genes.

## Teaser

**Summary:** Mouse and zebrafish genetic analyses and next-generation sequencing profiling identify a crucial conserved TFAP2-*ALX* transcriptional axis within the midface developmental gene regulatory network.

## INTRODUCTION

The evolution of jaws in parallel with the development of cranial neural crest cells (CNCCs) has led to a breadth of facial shapes, enabling gnathostome vertebrates to thrive and exploit varied ecological niches (Brandon et al., 2023; Martik and Bronner, 2021; Square et al., 2017; York and McCauley, 2020). The ability of CNCCs to give rise to skeletal elements that are essential for mastication and to house numerous sensory organs is driven by the coordination of epigenetic priming and transcriptional responses to local signaling inputs during embryonic development, ultimately to establish positional gene regulatory networks (GRNs) (Clouthier et al., 2010; Kessler et al., 2023; Santagati and Rijli, 2003; Selleri and Rijli, 2023). Fine-tuning of these programs underlies facial shape variation across species; likewise, disruption of such processes can lead to severe craniofacial birth defects, including orofacial clefts.

In humans, orofacial clefts usually present as a lateral or bilateral cleft of the upper lip and primary palate, with or without cleft secondary palate, and occur in ∼1 in 700 births (Mai et al., 2019; Tolarova and Cervenka, 1998). Although much less prevalent, clefts can also occur at the midline of the medial- and upper-face region forming the forehead, nose and cheeks (Tessier, 1976), generating a spectrum of midface pathologies that includes frontonasal dysplasia (MIMs: 136760, 613451, 613456) (Farlie et al., 2016; Vargel et al., 2021). Mutations in genes encoding the ALX homeodomain-containing transcription factors (*ALX1*, *ALX3* and *ALX4*) are associated with frontonasal dysplasia and produce midfacial phenotypes in vertebrate species (Beverdam et al., 2001; Iyyanar et al., 2022; Lakhwani et al., 2010; Lyons et al., 2016; McGonnell et al., 2011; Mitchell et al., 2021; Qu et al., 1999; Yoon et al., 2022). Although ALX transcription factors regulate midfacial development, how they connect with other midface genes into regulatory nodes remains uncertain. Of further importance, midface-specific modules are postulated to drive facial shape variation in humans (Claes et al., 2018; Feng et al., 2021; Naqvi et al., 2022; Xiong et al., 2019). Therefore, uncovering and connecting these genes in the midface GRN remains central to understanding facial development, morphology and pathology.

Members of the TFAP2 transcription factor family are known central determinants of vertebrate CNCC development (de Croze et al., 2011; Knight et al., 2003, 2004; Li and Cornell, 2007; Rothstein and Simoes-Costa, 2020; Schorle et al., 1996; Van Otterloo et al., 2018; Zhang et al., 1996) as well as facial shape variation and evolution (Erickson et al., 2018; Naqvi et al., 2022; Prescott et al., 2015). TFAP2 paralogs share highly similar DNA-binding and dimerization domains, recognize similar DNA-binding motifs (5′-GCCNNNGGC-3′), and are able to form both homo- and heterodimers, all of which suggest functional redundancy (Eckert et al., 2005; Liu et al., 2023; Williams et al., 1988; Williams and Tjian, 1991). The importance of cumulative TFAP2A and TFAP2B function in CNCCs during facial development is typified by animal models (Brewer et al., 2004; Knight et al., 2005; Li and Cornell, 2007; Martino et al., 2016; Van Otterloo et al., 2018). Furthermore, biochemical studies suggest that dominant mutations in *TFAP2A* or *TFAP2B* – resulting in branchio-oculo-facial syndrome (MIM: 113620) (Milunsky et al., 2008) or Char syndrome (MIM: 169100) (Satoda et al., 2000), respectively – create mutant proteins that may interfere with other TFAP2 paralogs (Li et al., 2013; Liu et al., 2023). The syndromes share specific phenotypes, such as hypertelorism, a broad nasal tip, and an abnormal philtrum and nasal bridge, supporting a role for both TFAP2A and TFAP2B in midfacial development. Because TFAP2 has been shown to modulate homeobox transcription factor gene expression in the pharyngeal arch CNCCs (Knight et al., 2004, 2003; Maconochie et al., 1999; Van Otterloo et al., 2018), we hypothesized that similar mechanisms operate in midface CNCCs.

In this study, we extend our analysis of TFAP2 function in midfacial development and gene regulation. Our findings show that similar midfacial clefting phenotypes are observed when *Tfap2a* and *Tfap2b* are lost either early or late in mouse neural crest development. This suggests that the crucial aspects of their function reside in post-migratory processes, such as proliferation, patterning and differentiation. By integrating mouse and zebrafish models with genome-wide molecular approaches, we further link TFAP2 with ALX family expression and function, placing them firmly in a GRN that underlies midface development, pathology and evolution.

## RESULTS

### TFAP2A and TFAP2B cooperatively function in CNCCs during midface development

We have previously identified that simultaneous loss of *Tfap2a* and *Tfap2b* in the murine neural crest results in clefting of midface and jaw elements (Van Otterloo et al., 2018). Subsequent profiling focused on the disruption of GRNs patterning the latter, whereas the role of TFAP2 during midfacial development was not pursued in detail. These studies also deployed a combination of null and floxed alleles, such that a lack of TFAP2 genes in the neural crest was accompanied by their heterozygosity in the remaining embryo (Fig. S1A). As loss of *Tfap2a* and *Tfap2b* in surface ectoderm disrupts development of the underlying CNCCs during facial development (Van Otterloo et al., 2022), we wished to ascertain whether loss of these two genes solely in the CNCCs was sufficient to cause midfacial clefting.

**Fig. 1.**
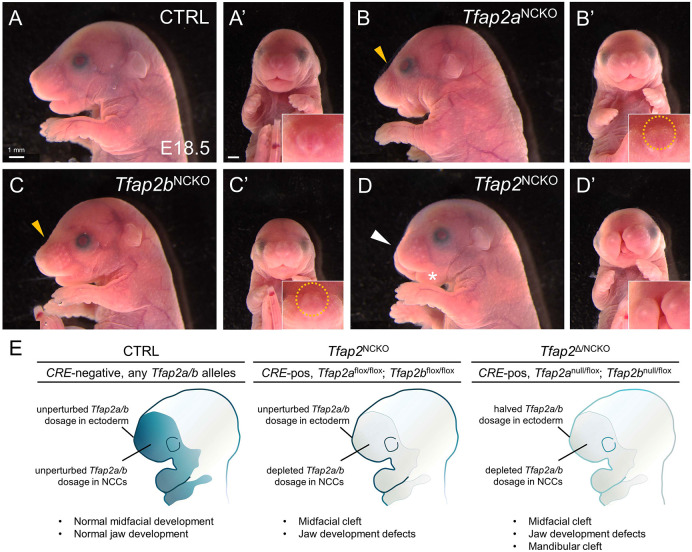
**TFAP2A and TFAP2B cooperatively function in CNCCs during midfacial development.** (A-D′) Lateral (A-D) or frontal (A′-D′) views of prenatal mice on embryonic day 18.5 (E18.5). Genotypes are as follows: CTRL (*CRE*-negative, any *Tfap2a/b* allelic combination; *CRE*-positive; *Tfap2a*^+/flox^; *Tfap2b*^+/flox^), *Tfap2a*^NCKO^ (*CRE*-positive; *Tfap2a*^flox/flox^; *Tfap2b*^+/flox^), *Tfap2b*^NCKO^ (*CRE*-positive; *Tfap2a*^+/flox^; *Tfap2b*^flox/flox^) and *Tfap2*^NCKO^ (*CRE*-positive; *Tfap2a*^flox/flox^; *Tfap2b*^flox/flox^). *n*=5 per genotype. Gold arrowheads in B and C indicate snout indentations. Insets in A′-D′ include higher magnification images of the snout, with misplaced vibrissae outlined. White arrowhead in D indicates the shortened snout; the asterisk indicates mandible hypoplasia. (E) Schematic summarizing regions of *Tfap2a* and *Tfap2b* gene deletion in various craniofacial tissues and the corresponding phenotypes (middle, this study; right, Van Otterloo et al., 2018). Regions of deletion are indicated by reduced color intensity.

To test this, we used only floxed alleles of *Tfap2a* and *Tfap2b* in combination with neural crest specific *CRE* transgenes (Fig. S1B). First, using *Wnt1:CRE* (Danielian et al., 1998), we conditionally deleted various allelic combinations of TFAP2 genes in pre-migratory neural crest cells and examined gross facial development. *CRE*-negative control embryos at embryonic day 18.5 (E18.5) displayed typical midface morphology that includes laterally positioned vibrissae and an elongated snout (Fig. 1A,A′). In *CRE*-positive littermates, *Tfap2a*^flox/flox^; *Tfap2b*^+/flox^ (*Tfap2a*^NCKO^) and *Tfap2a*^+/flox^; *Tfap2b*^flox/flox^ (*Tfap2b*^NCKO^) backgrounds resulted in mild and overlapping midfacial anomalies, such as an indented snout (Fig. 1B,C, gold arrowhead) and misplaced vibrissae on the nasal dorsum (Fig. 1B′,C′, circled). On the other hand, *CRE*-positive *Tfap2a*^flox/flox^; *Tfap2b*^flox/flox^ (*Tfap2*^NCKO^) mutants displayed a fully penetrant midfacial cleft and mildly shortened snout (Fig. 1D; white arrowhead). Because of the cleft, we could not identify by gross morphology whether *Tfap2*^NCKO^ embryos still harbored misplaced vibrissae atop the nares (Fig. 1D′). Earlier timepoints (E11.5, E12.5 and E15.5) revealed similar phenotypic trends, with the cleft becoming evident at E11.5 (Fig. S2A-K).

Gross examination and skeletal analyses of *Tfap2*^NCKO^ embryos revealed that they recapitulate the major jaw abnormalities we documented in the null allele (Fig. 1D, asterisk; Fig. S3A-E,E′) (Van Otterloo et al., 2018), with the exception that a mandibular cleft was not observed. Thus, we surmised that although the midface cleft and jaw defects can be attributed solely to loss of *Tfap2a* and *Tfap2b* in the neural crest, overt mandibular clefting is caused by reduced gene dose elsewhere – presumably the ectoderm (Fig. 1E). Although the remainder of our study primarily focused on embryos acquired from the flox/flox scheme (Fig. S1B), embryos acquired from the scheme containing both null and floxed alleles are indicated with ^Δ^ (Fig. S1C).

In summary, anomalies such as the midfacial cleft in *Tfap2*^NCKO^ embryos suggest that proper facial development relies on sufficient TFAP2 gene dose specifically in CNCCs. As observed in other neural crest stages and populations (Rothstein and Simoes-Costa, 2020; Schmidt et al., 2011; Seberg et al., 2017; Van Otterloo et al., 2018), these findings raise the hypothesis that TFAP2A and TFAP2B cooperatively function in GRNs typifying the midface region.

### TFAP2 in post-migratory CNCCs is required for proper midfacial development

TFAP2 paralogs are postulated to be crucial transcription factors operating throughout CNCC development, binding at genes poised to be activated upon their arrival in the face (Rada-Iglesias et al., 2012; Rothstein and Simoes-Costa, 2020). We next tested whether proper midfacial development depended on TFAP2 activity throughout CNCC development or at specific stages. Previous assessment at E9.0 and E10.0 using *r26r-lacZ*-mediated β-galactosidase staining revealed no overt changes in CNCC migration into the facial prominences between *Tfap2*^Δ/NCKO^ and control embryos (Van Otterloo et al., 2018). Using the same lineage tracing strategy in flox/flox embryos, we observed robust β-galactosidase staining in the E10.5 midface (i.e. successful CNCC occupancy) in the examined genotypes. However, *Tfap2*^NCKO^ embryos presented subtle differences in staining distribution and midface morphology compared with *Tfap2a*^NCKO^, *Tfap2b*^NCKO^ and *CRE*-positive *Tfap2a*^+/flox^; *Tfap2b*^+/flox^ control littermates (i.e. *Tfap2*^HET^) (Fig. S4A-D, white arrowhead). Compared with controls, *Tfap2*^NCKO^ mutants presented reduced cell proliferation in the E11.5 midfacial CNCCs, as marked by phospho-histone H3 immunofluorescence (Fig. S4E-G), whereas no cell death was observed by TUNEL staining (Fig. S4H-I′). In summary, lineage tracing revealed subtle changes in midfacial CNCC distribution in *Tfap2*^NCKO^ embryos relative to controls, possibly owing to reduced rates of proliferation.

**Fig. 2.**
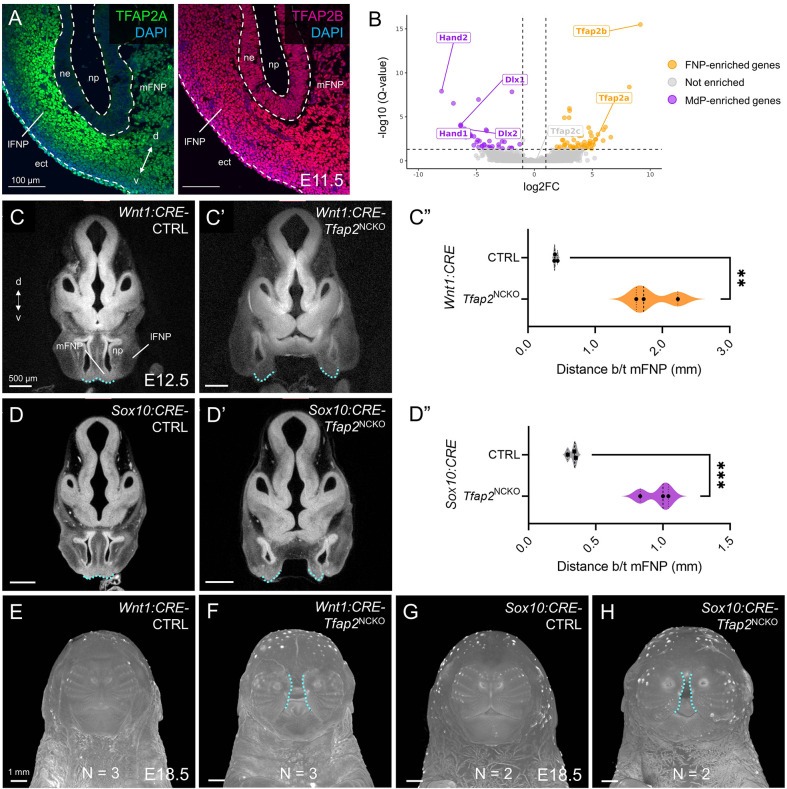
**A prominent role for TFAP2A and TFAP2B in post-migratory midfacial CNCCs.** (A) E11.5 wild-type midface tissue sections (horizontal angle) showing immunofluorescence of TFAP2A (left) and TFAP2B (right). White dashed lines indicate the ectoderm-mesenchyme boundaries. (B) Volcano plot displaying differentially expressed genes between E11.5 CNCCs occupying the mandibular prominence (MdP, purple) or frontonasal prominence (FNP, gold). (C-D″) Micro-computed tomography sections of E12.5 control-*Tfap2*^NCKO^ littermate pairs in *Wnt1:CRE* (C-C′) and *Sox10:CRE* (D-D′) schemes. Blue dashed lines outline the medial domains of the FNP (mFNP), with the distance between them quantified and visualized as violin plots in C″ and D″. *n*=3 per genotype. Unpaired Student's *t*-test, **P<0.01, ***P<0.001. (E-H) Front view of micro-computed tomography-scanned E18.5 embryos, with indicated genotypes and sample sizes. Blue dashed lines highlight the midfacial cleft. d, dorsal; ect, ectoderm; lFNP, lateral FNP; ne, nasal epithelium; np, nasal pit; v, ventral.

In addition to *Tfap2a* and *Tfap2b* gene expression in pre-migratory and migratory CNCCs (Mitchell et al., 1991), we observed TFAP2A and TFAP2B protein expression in the E11.5 midface mesenchyme with overlapping patterns (Fig. 2A; Fig. S5). Reanalysis of published E11.5 CNCC transcriptomes (Hooper et al., 2020) showed *Tfap2a* and *Tfap2b* transcript levels persist longer in the frontonasal prominence compared with the mandible prominence (Fig. 2B), suggesting that TFAP2 harbors additional functions after midfacial CNCC migration. To test this genetically, we took advantage of the *Sox10:CRE* driver (Matsuoka et al., 2005) to inactivate TFAP2 genes in late-migratory stages (∼E9.0) (Hari et al., 2012; Jacques-Fricke et al., 2012). Micro-computed tomography analyses revealed that, like *Wnt1:CRE-Tfap2*^NCKO^ embryos (Fig. 2C-C″, Fig. S6A,A′,C), *Sox10:CRE-Tfap2*^NCKO^ embryos presented with a midface cleft and nasal morphology defects, relative to controls (Fig. 2D-D″, Fig. S6B,B′). Pre-migratory loss (i.e. *Wnt1:CRE*) of TFAP2 genes had a slightly more pronounced effect on cleft severity and midfacial outgrowth at later stages (Fig. 2C″,D″,E-H, Fig. S6D-G), consistent with earlier roles for TFAP2 in CNCC development. Nevertheless, our combined expression and *Sox10:CRE* studies suggest that proper midfacial development relies on TFAP2 in post-migratory CNCCs.

### Appropriate formation of the midfacial skeleton depends on *Tfap2a* and *Tfap2b* gene dose in post-migratory CNCCs

**Fig. 3.**
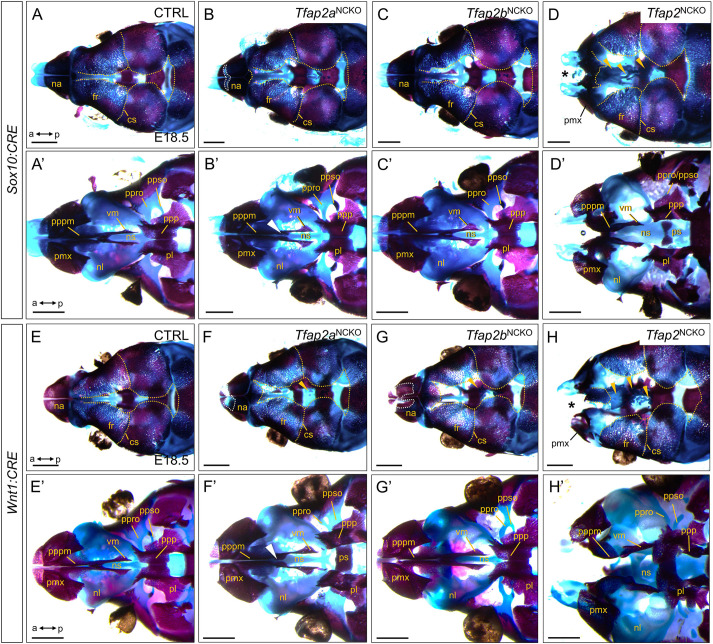
**Formation of midfacial bone and cartilage structures depends on *Tfap2a* and *Tfap2b* gene dose in CNCCs.** (A-H′) Alizarin Red (bone) and Alcian Blue (cartilage) staining preparations of E18.5 *Sox10:CRE* (A-D) or *Wnt1:CRE* (E-H) animals, with indicated genotypes and maxilla removed. *n*=5 per genotype. Scale bars: 1 mm. Anterior (a) is towards the left, posterior (p) towards the right. (A-H) Top-down views of the midfacial skeleton. Gold dashed lines (A-H) outline the peripheral edges of the calvaria bones. White dashed lines (B,F,G) outline nasal bone gaps. Gold arrowheads (D,F-H) indicate cartilaginous ectopias adjacent to the frontal bones. Asterisks (D,H) highlight missing nasal bones. (A′-H′) Bottom-up views of the midfacial skeleton. White arrowheads (B′,F′) indicate a gap in the vomer bones. cs, coronal suture; fr, frontal bone; na, nasal bone; nl, nasal/ethmoid labyrinth; ns, nasal septum; pl, palatine bone; pmx, premaxilla; ppp, palatal process of the palatine; pppm, palatal process of the premaxilla; ppro, pila preoptica; ppso, pila postoptica; ps, presphenoid; vm, vomer bone.

We next sought to leverage our transgenic models to better understand the developmental timing of TFAP2 during midfacial bone and cartilage formation. Accordingly, we compared E18.5 skeletal preparations of control, *Tfap2a*^NCKO^, *Tfap2b*^NCKO^ and *Tfap2*^NCKO^ backgrounds. Detailing the midfacial skeleton first in *Sox10:CRE* embryos, controls showed appropriate ossification of frontal and nasal bones interfacing at the midline (Fig. 3A). Underneath the cranium lies the primary palate formed by the premaxilla bones and vomer bones (Fig. 3A′). The nasal capsules are joined, connected to a compact cartilaginous nasal (or ethmoid) labyrinth and septum that sit adjacent to the anterior presphenoid (Fig. 3A′). *Tfap2a*^NCKO^ and *Tfap2b*^NCKO^ mutants exhibited small ectopic cartilage islands forming on the calvaria and mildly increased gaps between the frontal bones (Fig. 3B,C). Comparing *Tfap2a*^NCKO^ and *Tfap2b*^NCKO^ mutants, the former exhibited nasal bone gaps and a mild split in the vomer bones (Fig. 3B,B′, white arrowhead; Fig. 3C,C′). By contrast, *Tfap2*^NCKO^ embryos lacked nasal bones, exposing the underlying cleft nasal capsules (Fig. 3D, asterisk). Large, discontinuous cartilage ectopias were also found in between the hypoplastic frontal bones that, based on some individuals, appeared to stem from the nasal septum as dorsal projections (Fig. 3D, gold arrowheads; Fig. S7A, A′). Interestingly, these ectopias were replaced by severe frontal bone fractures in one individual (Fig. S7A″). Additionally, *Tfap2*^NCKO^ mutants presented clefting of the primary palate bones and malformed presphenoid bone (Fig. 3D′; Fig. S7B-B″, dashed lines), inflation of the posterior nasal labyrinth, and thickened nasal septum (Fig. S8A,B, arrowheads).

Examining the midface elements in *Wnt1:CRE* animals, *Tfap2a*^NCKO^ and *Tfap2b*^NCKO^ single mutants presented largely overlapping phenotypes as their *Sox10:CRE* equivalents, although with slight differences (Fig. 3E-G′). For example, compared with controls (Fig. 3E,E′), both single conditional mutants share gapped nasal bones and mishappened cartilage scaffolds connecting the frontal bone and anterior cranial base (pila pre/postoptica) (Fig. 3F,-G′). Strikingly, *Wnt1:CRE-Tfap2*^NCKO^ embryos recapitulated nearly all severe phenotypes observed in their *Sox10:CRE* equivalent, including missing nasal bones, smaller frontal bones, cartilaginous cranial ectopias, cleft primary palate bones, inflated nasal labyrinths and a thickened nasal septum (Fig. 3H,H′, Fig. S8C,D, arrowheads). Finally, although the jaw phenotypes were largely shared between *CRE* models (Figs S9A-D, S3), *Sox10:CRE*-*Tfap2*^NCKO^ mutants exhibited lower penetrance for fusion of the upper and lower jaw (i.e. syngnathia) (one out of five skeletons) (Fig. S9E), whereas fusion between maxillary and jugal bones was more frequent (three out of five skeletons) (Fig. S9F) relative to *Wnt1:CRE*-*Tfap2*^NCKO^ mutants (five out of five skeletons for syngnathia, 0 of 5 skeletons for maxilla-jugal bone fusions) (Fig. S3E′).

In sum, although we suspect that minor phenotypic distinctions between *Sox10:CRE* and *Wnt1:CRE* skeletons are due to timing or to small CNCC subpopulations uniquely labeled by each *CRE* (Debbache et al., 2018), remarkable similarities exist. The overlap in major defects between *CRE* models further emphasizes a crucial dependence on post-migratory CNCC-specific *Tfap2a* and *Tfap2b* gene dose during skeletal differentiation and patterning events.

### Combined *Tfap2a* and *Tfap2b* loss dysregulates numerous midface GRN components, including frontonasal dysplasia-related *Alx1*, *Alx3* and *Alx4* genes

**Fig. 4.**
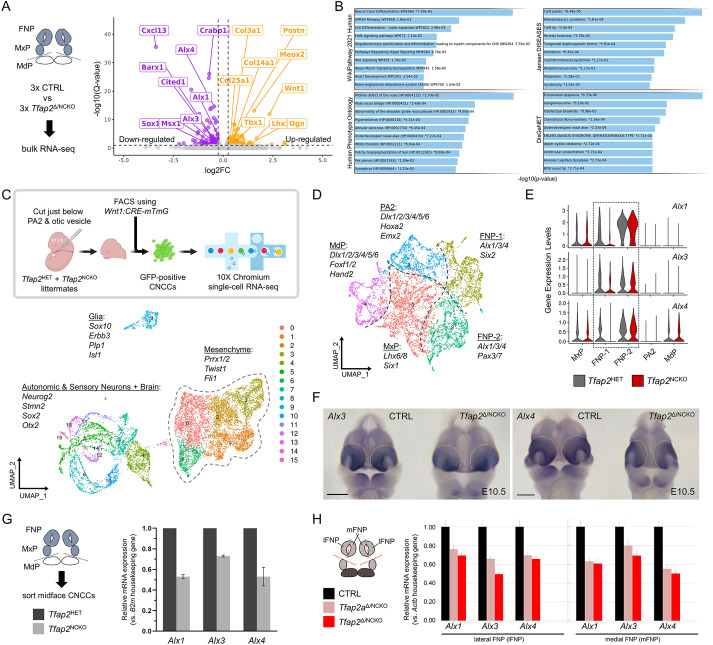
**Transcriptomic analyses reveal loss of TFAP2 genes compromises midface *Alx1*, *Alx3* and *Alx4* gene expression.** (A) Bulk RNA-seq volcano plot of frontonasal and maxillary prominence (FNP/MxP) tissues isolated from E10.5 control and *Tfap2*^Δ/NCKO^ littermates (cartoon workflow on the left). Downregulated (purple) and upregulated (gold) genes are shown. (B) Enrichr (Kuleshov et al., 2016) terms based on the RNA-seq downregulated genes. (C) Overview of the scRNA-seq experiment performed on E11.5 *Tfap2*^HET^ controls and *Tfap2*^NCKO^ mutants. The top box displays the workflow of sorting GFP-positive head CNCCs from pharyngeal arch 2 (PA2) and rostral of, and including, PA2. Created using Biorender.com. Below is the resulting uniform manifold approximation and projection (UMAP) plot of the three major groupings and genes enriched in each. The mesenchyme population is outlined and is further subset and re-clustered (D) using MAGIC imputation (van Dijk et al., 2018). Dashed lines on the mesenchyme UMAP indicate the positional identities, with their respective gene signatures. These include clusters for the FNP (two generated), MxP, mandibular prominence (MdP) and PA2. (E) Violin plots of *Alx1*, *Alx3* and *Alx4* gene expression between conditions and divided based on clusters. The FNP clusters are outlined. (F) Frontal view of whole-mount *in situ* hybridizations for *Alx3* and *Alx4* between E10.5 controls and *Tfap2*^Δ/NCKO^ mutants. Scale bars: 500 µm. Dashed lines outline the FNP. (G,H) Real-time PCR analysis for *Alx1*, *Alx3* and *Alx4* gene expression based on cDNA generated from E11.5 FNP/MxP CNCCs sorted from *Tfap2*^HET^-*Tfap2*^NCKO^ pairs (G) or E10.5 medial and lateral FNP (mFNP, lFNP) bulk tissue isolated from control-*Tfap2*^Δ/NCKO^ pairs (H).

Our results revealed that TFAP2A and TFAP2B act within a GRN in post-migratory midfacial CNCCs, but the genes regulated by these transcription factors were unknown. To address this, we compared the transcriptomic profiles of controls and mutants during crucial timepoints for midface patterning, including bulk RNA-seq of E10.5 midface tissue and scRNA-seq of E11.5 CNCCs. With respect to the first approach, comparison between *CRE*-negative control and *Tfap2*^Δ/NCKO^ mutants uncovered 149 genes to be dysregulated between groups (86 downregulated, 63 upregulated) (Fig. 4A; Table S1). Ontology, enrichment and pathway analysis via Enrichr (Kuleshov et al., 2016) revealed an over-representation of midfacial pathology terms associated with downregulated genes (Fig. 4B), many containing homeobox transcription factor genes such as *Msx1* (Han et al., 2007; Ishii et al., 2005; Roybal et al., 2010) as well as *Pax3* and *Pax7* (Table S1) (Gu et al., 2022; Zalc et al., 2015). Notably, terms such as ‘midline defect of the nose’ (*P*=2.00e-05, adj. *P*<0.05) as well as ‘frontonasal dysplasia’ (*P*=6.33e-06, adj. *P*<0.05) contained *Alx1*, *Alx3* and *Alx4* (Fig. 4A,B), the combinatorial loss of which leads to midfacial clefting that phenocopies that found in *Tfap2*^NCKO^ mutants (Beverdam et al., 2001; Gu et al., 2022; Iyyanar et al., 2022; Lakhwani et al., 2010; Qu et al., 1999). *Crabp1* and *Aldh1b1*, encoding regulators of a retinoic acid pathway linked to midfacial clefting (Fig. 4A; Table S1) (Gao et al., 2021; Kennedy and Dickinson, 2012; Lohnes et al., 1994; Williams and Bohnsack, 2019; Wu et al., 2022), were also reduced. Unexpectedly, among the upregulated genes were those encoding collagens (e.g. *Col14a1* and *Col3a1*), periostin (*Postn*), osteoglycin (*Ogn*) and fibronectin leucine rich transmembrane protein 2 (*Flrt2*) (Fig. 4A; Table S1), all components that are involved in skeletal formation (Bhatt et al., 2013; Dash and Trainor, 2020), suggesting premature activation of this process. Thus, by E10.5, transcriptional changes for genes associated with midfacial anomalies are already emerging.

Next, we conducted scRNA-seq of CNCCs sorted from the heads of *Tfap2*^HET^ and *Tfap2*^NCKO^ littermates (Fig. 4C). Integration and Uniform Manifold Approximation and Projection (UMAP) plotting of control and mutant genotypes generated 16 clusters divided into three major cell groupings (Fig. 4C). Concordant with *Wnt1:CRE* cell labelling and other scRNA-seq studies (Jacques-Fricke et al., 2012; Soldatov et al., 2019), these cells clustered into groups with signatures for glia (e.g. *Sox10*, *Erbb3*, *Plp1* and *Isl1*), neurons and components of the brain (e.g. *Neurog2*, *Stmn2*, *Sox2* and *Otx2*), and the mesenchyme (e.g. *Prrx1*, *Prrx2*, *Twist1* and *Fli1*) (Fig. 4C, Fig. S10A-D). Although total cell numbers between conditions were not drastically altered, mutant glial/mesenchymal populations were reduced (∼7-8% each) whereas neuronal populations increased (∼12%) (Fig. S11A). Gene expression-based cell cycle scoring did not detect noticeable differences between control and mutants (Fig. S11B), indicating cellular profiles between genotypes are not due to altered proliferation states between CNCC derivatives. We conclude that although TFAP2 may bias CNCCs towards particular fates, their loss alone was not sufficient to drive drastic switches in the same way as previously reported factors (Cox et al., 2012; Fan et al., 2021; Scerbo and Monsoro-Burq, 2020; Simoes-Costa and Bronner, 2016; Soldatov et al., 2019).

To identify gene expression differences in the mesenchyme group (Fig. 4C, outlined), we employed a ‘pseudobulk’ approach. This analysis identified 383 dysregulated genes (124 downregulated, 259 upregulated; log fold-change threshold=0.1, adj. *P*<0.05) that contained overlaps with E10.5 RNA-seq profiling (Table S1). For example, downregulated genes yielded several Enrichr terms associated with transcription factor gene regulation and craniofacial features associated with midface factors (e.g. *Msx1*, *Pax3*, *Pax7*, *Alx1* and *Alx4*) (Fig. S12A, Table S1). Although *Alx3* did not appear in our pseudobulk dataset, we suspect this is partly due to a combination of it being the least expressed paralog in the whole mesenchyme and the limited sequencing depth. In the terms for upregulated genes, there was an enrichment of the forkhead domain term coming from various Fox family members (e.g. *Foxd1*, *Foxf2*, *Foxp1* and *Foxp2*) (Fig. S12B, Table S1). These genes are downstream targets of a Hedgehog signaling pathway (Everson et al., 2018; Jeong et al., 2004) associated with midfacial clefting when precociously activated (Brugmann et al., 2010; Firulli et al., 2014; Xu et al., 2022, 2018). Furthermore, we continued to observe several extracellular matrix terms from elevated transcripts of multiple collagen genes (e.g. *Col1a1*, *Col2a1*, *Col3a1* and *Col4a1*) (Fig. S12B, Table S1).

Confirming the positional specificity of transcript changes, we mapped expression levels of select genes on the mesenchyme UMAP that was computationally re-clustered via MAGIC imputation (Fig. 4D, Fig. S13A-G) (van Dijk et al., 2018). Consistent with our previous findings and other past studies (Knight et al., 2003; Maconochie et al., 1999; Van Otterloo et al., 2018), mutant lower face CNCC populations showed reduced cell numbers and gene expression changes (Fig. S14A-B, Table S1). Among the two midface clusters (enriched for *Tfap2a*, *Tfap2b*, *Alx1*, *Alx3*, *Alx4*, *Six2*, *Pax3* and *Pax7*) (Figs S13F,G, S15A,B), mutant cells were enriched in one (frontonasal cluster 1 or FNP-1) and reduced in the other (frontonasal cluster 2 or FNP-2) (Fig. S14A,A′). Linked to these changes in the midface clusters were decreased levels of *Alx1*, *Alx3*, *Alx4* (Fig. 4E), *Msx1*, *Pax3* and *Pax7*, concomitant with the upregulated collagen and Fox genes (Fig. S14C). Finally, orthogonal approaches such as whole-mount *in situ* hybridization (Fig. 4F) and real-time PCR (Fig. 4G,H) validated that, compared with controls, TFAP2 mutants exhibited reduced midfacial *Alx1*, *Alx3* and *Alx4* expression.

In total, profiling the loss of TFAP2 genes in CNCCs by transcriptomics identified dysregulation of numerous transcription factor and signaling pathway genes within the midface positional program. These data reveal that TFAP2 paralogs regulate midface patterning, morphogenesis and skeletal formation through a range of genes that include the frontonasal dysplasia-associated *Alx1*, *Alx3* and *Alx4*.

### TFAP2 plays a direct role in the midface GRN in part by occupying *Alx1*, *Alx3* and *Alx4* regulatory elements

**Fig. 5.**
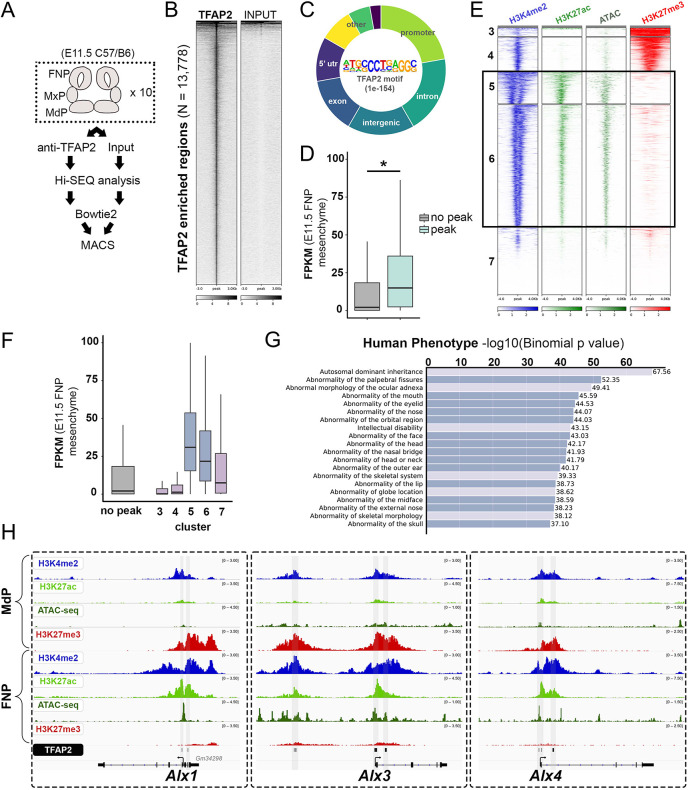
**ChIP-seq profiling identifies TFAP2 paralogs directly bind *Alx1*, *Alx3* and *Alx4* regulatory elements.** (A) Cartoon workflow for anti-TFAP2 ChIP-seq of wild-type E11.5 C57BL/6 facial prominence tissues. (B) Density heatmap displaying read-depth at the 13,778 TFAP2 ChIP-seq ‘peaks’ (*y*-axis) relative to non-immunoprecipitated input. (C) Hollow pie chart summarizing the distribution of TFAP2 ‘peaks’ throughout the genome, relative to key features (e.g. promoters, introns, etc.). The TFAP2 motif, along with the significance of its enrichment in all TFAP2 ‘peaks’, is displayed in the center. (D) Box-and-whisker graph plotting the FPKM values of 15,842 genes expressed in the E11.5 frontonasal prominence (FNP) mesenchyme (Hooper et al., 2020), with or without an associated TFAP2 peak (*P=2.60e-256, unpaired Wilcoxon test). (E) Density heatmaps for anti-H3K4me2 (blue), anti-H3K27ac (green), ATAC-seq (forest green) and anti-H3K27me3 (red) profiles of E10.5 FNP CNCCs (Minoux et al., 2017) at the TFAP2-positive coordinates, divided by k-means clustering. (F) Box-and-whisker graph, as in D, but with further partitioning the ‘peak’ group based on k-means clusters from E. Boxes represent the 1st and 3rd quartiles; whiskers represent the spread of values, excluding outliers (i.e. within 1.5× of the inter-quartile range). (G) GREAT (McLean et al., 2010) pathway analysis of genes associated with cluster 5. ‘Human Phenotype’ terms are listed, with craniofacial-specific terms in darker boxes. (H) IGV browser views of the *Alx1*, *Alx3* and *Alx4* loci overlaid with the epigenome signatures (blue, H3K4me2; green, H3K27ac; khaki, ATAC; red, H3K27me3) of E10.5 CNCCs residing in the mandibular prominence (MdP, top four tracks) and FNP (tracks 5-8), as well as the cluster 5-assigned TFAP2 peaks (bottom, gray highlights).

Our genome-wide transcriptomic analyses identified important genes that were dysregulated by TFAP2 gene loss in the midface CNCCs. To identify the subset directly regulated by TFAP2 paralogs, we conducted ChIP-seq in E11.5 whole facial prominence tissue using an antibody that recognizes TFAP2A, TFAP2B and TFAP2C (Fig. 5A, Fig. S16) (Jin et al., 2015; Schuur et al., 2001). We identified 13,778 enriched regions (i.e. TFAP2 ‘peaks’) relative to input (Fig. 5B, Table S2), while antibody specificity was reflected by the TFAP2 consensus binding motif being the most significantly enriched (*P*=1.00e-154) (Fig. 5C). Further analysis of TFAP2 binding coordinates revealed ∼35% of them were in proximal promoters, 23% were intronic, 16% were intergenic and 9% were in the 5′ UTR, while remaining peaks were found in additional genomic regions (Fig. 5C). Because we isolated all facial prominences, these peaks presumably reflect unique or shared targets within mesenchyme from each region, as well as those present in other tissues, such as ectoderm and neuronal tissue. To focus on genes relevant to the midface, we cross-referenced these peak coordinates with the E11.5 midface mesenchyme transcriptome (Hooper et al., 2020). Of the 15,842 genes with detectable midface expression (i.e. a recorded FPKM value), the 8754 genes associated with at least one TFAP2 peak were expressed in the midface at a significantly higher level than the remaining 7088 midface genes without a TFAP2-associated peak (*P*=2.60e-256) (Fig. 5D, Table S2). Furthermore, despite the unique features of different datasets (e.g. E10.5 versus E11.5, CNCC-only versus whole face), ∼66% (76 of 115) of the genes dysregulated in our overlapping bulk and scRNA-seq dataset were associated with a TFAP2 peak (Table S2).

We next assessed the epigenome status at these ∼13,800 genomic regions with the prediction that a subset of these coordinates would be found within midface-expressed, CNCCs-associated, accessible and ‘active’ regions. Using k-means clustering along with previously published histone ChIP- and ATAC-seq datasets generated from wild-type E10.5 midface CNCCs (Minoux et al., 2017), five major clusters (clusters 3, 4, 5, 6 and 7; clusters 1 and 2 had fewer than six peaks) were identified (Fig. 5E, Table S2). Of these five clusters, over half the TFAP2-bound regions (*n*=7995; 58% of all peaks) clustered into clusters 5 and 6 (Fig. 5E, Table S2). Both contained substantial levels of the active H3K4me2 and H3K27ac, open chromatin, and marked reduction in the repressive H3K27me3 mark (Fig. 5E). In contrast to clusters 5 and 6, clusters 3 and 4 displayed substantially reduced levels of open chromatin and active H3K27ac, accompanied by expanded H3K27me3 signal (i.e. repressed regions in the E10.5 frontonasal prominence CNCCs) (Fig. 5E, Table S2), whereas cluster 7 was not enriched for any specific signatures (Fig. 5E). Consistent with these profiles, genes associated with clusters 5 and 6 were highly expressed in the midface, relative to genes without a TFAP2 peak or to genes associated with a peak(s) found in other clusters (Fig. 5F, Table S2). Pathway analysis for cluster 5 (1658 peaks) and cluster 6 (6336 peaks) revealed terms associated with head and midfacial pathology (Fig. 5G). Notably, genes associated with clusters 3 and 4 were significantly enriched for skin (cluster 4), neuronal (clusters 3 and 4) and cardiac (cluster 3) terms (Fig. S17), potentially reflecting TFAP2 binding in non-NCC-derived tissues in which TFAP2 is expressed.

Finally, to identify whether TFAP2 regulation of *Alx1*, *Alx3* and *Alx4* gene expression is direct or indirect, peak files revealed that all three ALX loci contained TFAP2 peaks (Fig. 5H). These regions were associated with cluster 5, concordant with active and open genomic regions at the site of TFAP2 binding (Fig. 5H); moreover, integration of mandibular CNCC datasets showed reduced H3K4me2/H3K27ac and increased H3K27me3 (Fig. 5H), consistent with limited *Alx1*, *Alx3* and *Alx4* levels in this region (Minoux et al., 2017). Overall, our ChIP-seq analysis suggests a direct mode of regulation by TFAP2 paralogs for genes associated with midfacial patterning and differentiation, i.e. *Alx1*, *Alx3* and *Alx4*.

### Zebrafish *tfap2a* regulates *alx3* expression and genetically interacts with *alx3* during midfacial development

Although different vertebrates use different TFAP2 paralogs during CNCC development, a member commonly used is TFAP2A (Eckert et al., 2005; Meulemans and Bronner-Fraser, 2002). Like humans and mice, the zebrafish *tfap2a* paralog is a crucial determinant for the formation of craniofacial structures (Knight et al., 2003; Neuhauss et al., 1996; O'Brien et al., 2004; Schilling et al., 1996). Re-examining our published zebrafish CNCC scRNA-seq datasets (Stenzel et al., 2022), we observed *tfap2a* gene expression predominantly in the frontonasal cluster (i.e. midface) (Fig. S18A). Like mice (Fig. S15A,B), co-expression analyses indicated that *tfap2a* expression overlapped with *alx1*, *alx3* and *alx4a* (Fig. S18B), suggesting that TFAP2-mediated regulation of ALX gene expression is a conserved transcriptional axis in vertebrates.

**Fig. 6.**
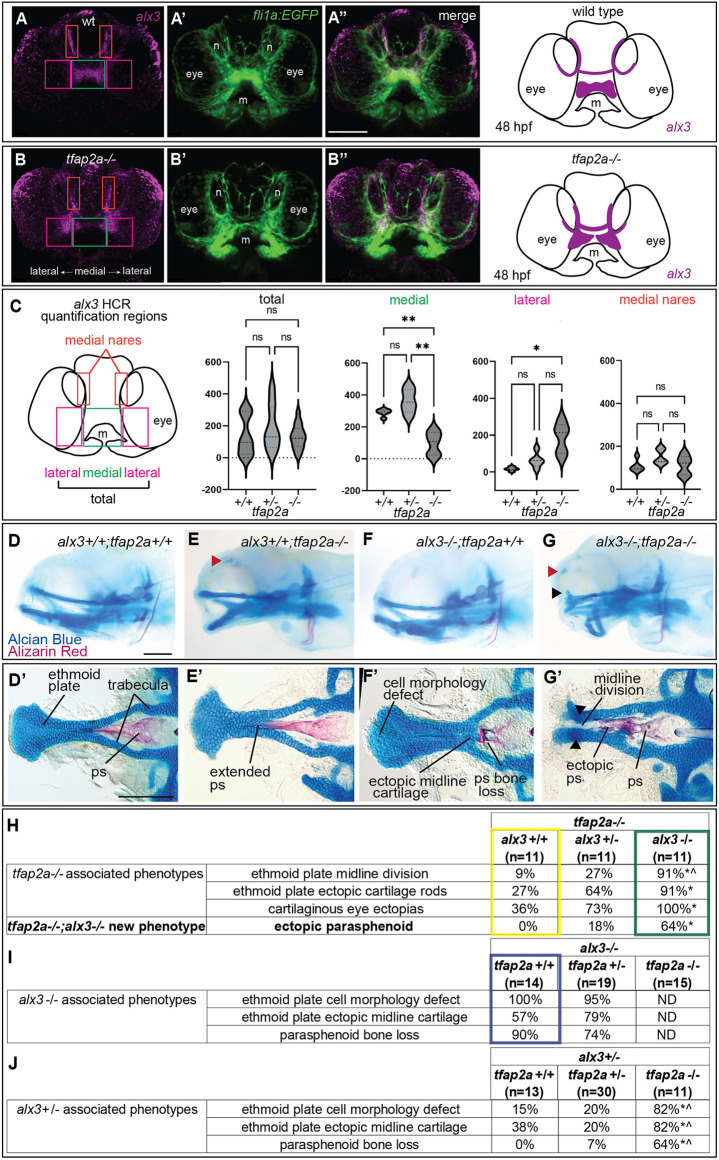
**Gene expression and epistasis studies indicate zebrafish *alx3* is genetically downstream of *tfap2a*.** (A-B″) Frontal views of zebrafish embryos at 48 h post-fertilization (hpf). *alx3 in situ* HCR (magenta, A,B), *fli1a:EGFP*-labelled cranial neural crest cells (green, A′,B′) or the merge (A″,B″) in a control (A-A″) and a *tfap2a*^−/−^ (B-B″) sibling. Schematics summarizing expression patterns observed for *alx3* are shown on the far right, *n*=5 per genotype. Boxes in A and B show regions used for quantification in C. (C) Quantification of HCR signal in distinct domains of the larval head, as indicated by boxed regions in the schematic. (D-G) Whole-mount, lateral views of Alizarin Red/Alcian Blue stained skeletal preparations of 6-day-old zebrafish heads, with indicated genotypes. The anterior is towards the left. (D′-G′) Neurocrania were dissected and flat mounted. Red arrowheads indicate ectopic cartilage near the eye socket. Black arrowheads mark dorsally projecting ectopic ethmoid plate cartilage. (H-J) Tables summarizing penetrance, sample sizes and statistics of the epistasis experiments as follows: (H) the removal of wild-type *alx3* copies from *tfap2a* homozygous mutants; (I) removal of wild-type *tfap2a* copies from *alx3* homozygous mutants; (J) removal of wild-type *tfap2a* copies from *alx3* heterozygotes. Boxed values correspond to the genotypes shown in E, F′ and G. Yellow, *tfap2a* mutant phenotypes and their penetrance (corresponding to E, E′, G′); green, removal of both *alx3* copies in *tfap2a*-mutant backgrounds (corresponding to G, G′); purple, *alx3* mutants (F′). Asterisks indicate significant difference (*P*<0.05) compared with the far-left column and carets indicate significant difference (*P*<0.05) compared with middle column (Fisher's exact test). Scale bars: 100 µm. m, mouth; *n*, nares; ND, not determined; ps, parasphenoid.

To test this, we examined: (1) ALX gene expression in *tfap2a* mutants (Knight et al., 2003; Schilling et al., 1996) versus control siblings (Fig. 6A-C); and (2) Tfap2a binding at ALX loci in wild-type embryos (Fig. S19A-K). Regarding the first, we analyzed zebrafish embryos using *in situ* hybridization at 48 h post-fertilization, a stage approximating when ALX expression was disrupted in the mouse (e.g. E10.5 and E11.5). Given our previous findings of Alx3 function in zebrafish midfacial development (Mitchell et al., 2021), we focused on *alx3* as a potential Tfap2a target. We found *alx3* expression was enriched in *fli1a:EGFP*-labelled CNCCs of the developing roof of the mouth, as well as those ventral and medial to the nares in control embryos (Fig. 6A-A″). In contrast, *alx3* expression patterns were altered in *tfap2a* mutant embryos, although changes were regionally specific. For example, *alx3* expression was significantly reduced at the midline and expanded at the lateral domains residing under the nares, whereas expression in domains at the nares was unchanged (Fig. 6B-C, Table S3). Regarding the second, we conducted anti-Tfap2a CUT&RUN in 24 h post-fertilization wild-type embryos. To limit our analysis of Tfap2a binding to only CNCC-relevant regulatory elements, we intersected Tfap2a CUT&RUN ‘peaks’ with open chromatin associated with CNCCs, as previously detected by single-nuclei ATAC-seq (Fig. S19A-H) (Fabian et al., 2022). Indeed, Tfap2a was found to bind several regions associated with accessible chromatin at the *alx1*, *alx3* and *alx4a* loci (Fig. S19I-K). Overall, these findings suggest that *alx3* expression is directly sensitive to Tfap2a function.

The hypothetical regulatory axis between *tfap2a* and *alx3* predicts that these genes interact during midfacial development. Analysis of craniofacial skeletons in all mutant combinations clearly supports this prediction (Fig. 6D-J,D′-G′, Table S4). Consistent with previous studies, we identified the posterior pharyngeal arches as being particularly sensitive to the loss of *tfap2a*, while the pharyngeal arch 1 skeleton is less affected (Knight et al., 2003; Neuhauss et al., 1996; Schilling et al., 1996; Wang et al., 2011). We also confirmed that the parasphenoid bone extended anteriorly (Fig. 6E′) (Schilling et al., 1996). However, our *tfap2a* mutants did not show the previously reported ethmoid plate loss (Barrallo-Gimeno et al., 2004). Intriguingly, we discovered two incompletely penetrant *tfap2a* mutant-associated skeletal phenotypes that have not been previously described (Fig. 6H, boxed in yellow). Specifically, *tfap2a* mutant larvae exhibited cartilaginous ectopias adjacent to the eyes (Fig. 6E, red arrowhead, Fig. 6H) as well as ectopic cartilage rods projecting dorsally from the ethmoid plate (quantified in Fig. 6H). As *alx3* gene dose was reduced in *tfap2a* mutants, the penetrance for both phenotypes increased (Fig. 6G, red and black arrowheads, quantified in Fig. 6H). In addition, a new phenotype never seen in single mutants appeared: ectopic parasphenoid bone (Fig. 6G′,H, bold)*.* A selection of these skeletal abnormalities, the split ethmoid and ectopic cartilage, are reminiscent of those observed in our mouse models (see Fig. 3D,H for comparisons).

Reciprocally, we next documented how reducing *tfap2a* gene dose impacts *alx3* mutant-associated phenotypes, which include anterior ethmoid plate cell morphology defects, anterior parasphenoid bone loss and ethmoid plate ectopic midline cartilage (Fig. 6F′,I, boxed in purple) (Mitchell et al., 2021). In *alx3* mutant homozygotes with reduced *tfap2a* dose, the ethmoid plate and parasphenoid were severely impacted (Fig. 6I), precluding us from scoring *tfap2a*-dependent changes in double homozygous mutants*.* Therefore, we examined changes in these phenotypes in *alx3* heterozygotes when wild-type copies of *tfap2a* were removed. Like the enhancement of *tfap2a*-associated phenotypes upon *alx3* removal, we found that removing *tfap2a* from *alx3* heterozygotes increased penetrance of *alx3*-associated phenotypes (Fig. 6J). These findings demonstrate that *tfap2a* and *alx3* genetically interact during zebrafish midfacial development. Taken together with our observations in mice, these results raise a model that the TFAP2-ALX axis is a prominent, conserved node in the midface GRN.

## DISCUSSION

Positional gene regulatory networks (GRNs) endow CNCCs with their ability to form unique facial structures during embryogenesis. Despite the clear existence of GRN components directing midfacial development, our understanding of how they connect into transcription circuits is poorly understood. Integrating mouse and zebrafish genetic, transcriptomic and epigenomic studies, we show that TFAP2 transcription factors play an essential role in CNCCs during midfacial development, in part through activation of a conserved ALX genetic pathway. Given the spectrum of human midfacial anomalies associated with disrupted functions of *TFAP2A* (branchio-oculo-facial syndrome), *TFAP2B* (Char syndrome), and *ALX1*, *ALX3* and *ALX4* (frontonasal dysplasia), these findings link features of previously disparate disorders into a shared genetic hierarchy.

### TFAP2 and ALX paralogs in vertebrate midfacial morphology and evolution

Our examination in CNCCs alongside previous studies indicate that TFAP2A and TFAP2B reinforce unique GRN components specifying each subpopulation. Although *Dlx* (Knight et al., 2003; Van Otterloo et al., 2018) and *Hox* (Maconochie et al., 1999) homeobox genes are pharyngeal arch outputs of TFAP2, we have shown in this study that these transcription factors regulate *Pax3*, *Pax7*, *Msx1* and, of particular interest, *Alx1*, *Alx3* and *Alx4* in the midface. Intraspecies comparisons demonstrate the role of both *TFAP2* [stickleback (Erickson et al., 2018)] and *ALX* [Darwin's finches (Lamichhaney et al., 2015)] in species-specific facial variation. Furthermore, studies leveraging induced pluripotent stem-cell derived neural crest models (Feng et al., 2021; Prescott et al., 2015; Rada-Iglesias et al., 2012) suggest that TFAP2 and ALX contribute to craniofacial differences between primates and humans.

Our mouse and zebrafish data recontextualize these earlier studies and strengthen the notion that vertebrate midfacial shape variation is a direct output of these transcription factors. These cumulative findings also raise questions on the evolutionary origin of the TFAP2-ALX axis. TFAP2 (Meulemans and Bronner-Fraser, 2002) and ALX (Cattell et al., 2011) orthologs appear to be co-expressed in the lamprey facial complex. Thus, out of the recently reported TFAP2 target genes that contributed to CNCC evolution (Martik et al., 2019; Van Otterloo et al., 2012), the developmental pathway marked by ALX transcription factors merits testing in this context.

### Potential mechanisms of TFAP2- and ALX-mediated midface morphogenesis

How loss of the TFAP2-ALX axis disrupts midface morphogenesis remains unclear but may relate to the regulation of differentiation, cell survival and expression of other patterning genes. In sea urchin, ALX directly targets biomineralization and extracellular matrix genes (Khor and Ettensohn, 2020). In line with these observations, our previous cellular and skeletal analyses in zebrafish implicate Alx3 as a crucial determinant of midfacial chondrocyte differentiation timing (Mitchell et al., 2021). Similarly, here we observed in *Tfap2*^NCKO^ mouse embryos the increased expression of multiple collagen genes, which are a major readout of bone and cartilage formation (Dash and Trainor, 2020). Conceivably, aberrant cartilage differentiation in such embryos could prohibit appropriate midfacial growth and subsequent fusion in an ALX-dependent manner. In addition, *Alx1*, *Alx3* and *Alx3/Alx4* mutant mice exhibited elevated apoptosis in midface CNCCs (Beverdam et al., 2001; Lakhwani et al., 2010; Zhao et al., 1996), including the periocular mesenchyme (Iyyanar et al., 2022). Moreover, there is elevated cell death in neural crest lines derived from an *ALX1*-deficient individual versus those derived from controls (Pini et al., 2020). However, we did not observe cell death in midfacial CNCCs upon TFAP2 gene loss. Furthermore, *Alx1* and *Alx1/Alx4* mutant mice exhibited a reduction in *Pax7* and expanded *Lhx6* and *Lhx8* expression beyond the jaw domains and into the midface (Iyyanar et al., 2022), suggesting a role for these transcription factors in patterning. Our expression profiling of CNCCs in *Tfap2*^Δ/NCKO^ and *Tfap2*^NCKO^ embryos revealed a reduction in *Pax7* (and *Pax3*) expression, but not a concomitant expansion of *Lhx6* and *Lhx8* relative to CNCC from controls. How gene expression changes in TFAP2 and ALX paralog mutants translate to cellular defects will require follow-up studies.

### Potential TFAP2 interplay with other midface positional factors

In addition to ALX paralogs, signaling pathways are affected by loss of TFAP2. For example, we observed decreased levels of various retinoic acid-related genes, which is of interest because disruption of retinoic acid signaling also results in a midfacial cleft (Gao et al., 2021; Kennedy and Dickinson, 2012; Lohnes et al., 1994; Wu et al., 2022). As retinoic acid can modulate *TFAP2A* gene expression and activity *in vitro* (Luscher et al., 1989), TFAP2 may function in a regulatory feedback loop within this signaling cascade. Hedgehog signaling also appears to be dysregulated upon loss of TFAP2 genes. Although our expression profiling did not reveal significant changes in major hedgehog signaling pathway genes (e.g. *Shh*, *Ptch1, Ptch2*, *Gli1*, *Gli2*, *Gli3* and *Smo*), members in the Fox transcription factor family were upregulated. Fox factors are known downstream targets of hedgehog, and their transcription factor products function during pharyngeal arch CNCC patterning and cartilage differentiation (Jeong et al., 2004; Xu et al., 2022, 2018, 2021). Consistently, hyperactivation of Hedgehog activity is strongly linked with midfacial clefting (Brugmann et al., 2010; Wu et al., 2022). Thus, it is possible that the TFAP2-ALX axis modulates hedgehog signaling-mediated transcriptional responses.

Finally, TFAP2 may also function in parallel with other midface transcription factors. One such example is SIX2, the gene encoding which is expressed together with TFAP2 genes in the midfacial CNCCs and is largely unaffected by loss of TFAP2 genes. Loss of human *SIX2* is associated with frontonasal dysplasia (Hufnagel et al., 2016), while mice harboring a similar locus deletion exhibit a nasal cleft (Fogelgren et al., 2008)*.* Mouse genetic analyses suggest SIX paralogs contribute to ALX gene expression (Liu et al., 2019), thereby hinting at a transcriptional circuit running alongside TFAP2. Learning how TFAP2 integrates with other midface signaling pathways and GRN components will improve our understanding of how these transcription factors contributes to facial shape variation, vertebrate facial evolution and the pathogenesis of midfacial disorders.

## MATERIALS AND METHODS

### Animal husbandry and procedures

#### Mice

Experiments were all conducted in accordance with all applicable guidelines and regulations, following the ‘Guide for the Care and Use of Laboratory Animals of the National Institutes of Health’. The animal protocols used were approved by the Institutional Animal Care and Use Committee of the University of Iowa (protocols 9012197 and 2032197) and the University of Colorado (protocol #4). Noon on the day of an observable copulatory plug was denoted as embryonic day 0.5 (E0.5). Additionally, all dissections were performed in 1×PBS (Fisher Scientific) treated with diethyl pyrocarbonate (Fisher Scientific) (i.e. DEPC-PBS). Yolk sacs or tails clips were used to extract DNA for all PCR genotyping as previously described, with the listed primers (Table S5) (Van Otterloo et al., 2018). During single-cell RNA sequencing (scRNA-seq), we deployed a rapid lysis protocol using reagents from the SYBR Green Extract-N-Amp Tissue PCR Kit (Sigma) (Doan and Monuki, 2008). Yolk sacs were incubated in a 4:1 mixture of Extraction and Tissue Preparation Solutions first at room temperature for 10 min and subsequently at 95°C for 3 min. Neutralization Solution B was then added to the lysate, which was briefly vortexed before immediate PCR genotyping.

Embryonic lethality was denoted for a subset of E18.5 embryos in the *Wnt1:CRE* model (see the section ‘Mouse alleles and breeding schemes’, Table S6), which was a consequence of reduced norepinephrine and compromised survival of tissues in the sympathetic nervous system (Schmidt et al., 2011). To prevent this lethality, pregnant dams received a water supplement to drink *ad libitum* (Hendershot et al., 2008; Lim et al., 2000). USP grade L-phenylephrine (a β-adrenergic receptor agonist), isoproterenol (an α-adrenergic receptor agonist) and L-ascorbic acid (an agonist preservative) were added to their drinking water from E8.5 to E18.5, which was replaced every 3-5 days. As in previous studies (Abe et al., 2021), the supplementation did not alter craniofacial phenotypes in control or mutant embryos compared with those without supplementation.

#### Zebrafish

All work with zebrafish was approved by the University of Colorado Institutional Animal Care and Use Committee (protocol 00188). All zebrafish experiments were performed on the AB strain. Animals were maintained, staged and genotyped as previously described (Knight et al., 2003; Mitchell et al., 2021).

### Mouse alleles and breeding schemes

*Wnt1:CRE* (Danielian et al., 1998), *Sox10:CRE* (Matsuoka et al., 2005), *Gt(ROSA)26Sortm1Sor/J* [*r26r-lacZ;* (Soriano, 1999)] and *Gt(ROSA)26Sortm4(ACTB-tdTomato,-EGFP)Luo/J* [*mTmG;* (Muzumdar et al., 2007)] mice were obtained from the Jackson Laboratory. Based on published lineage-tracing studies (Hari et al., 2012; Jacques-Fricke et al., 2012; Stottmann et al., 2004), we note that *Wnt1:CRE* recombines in pre-migratory CNCCs (∼E8.0), whereas *Sox10:CRE* recombines when most CNCCs have finished migrating into the embryonic face (∼E9.0). The *Tfap2a* and *Tfap2b* alleles used in this study include a *Tfap2a* null (Zhang et al., 1996), *Tfap2atm2Will/J* [a *Tfap2a* floxed conditional (Brewer et al., 2004)], a *Tfap2b* null (Martino et al., 2016) and *Tfap2btm2Will* [a *Tfap2b* floxed conditional (Van Otterloo et al., 2018)]. All mice were kept on a mixed background.

*Tfap2a*^flox/flox^; *Tfap2b*^flox/flox^ females were used in this study. For lineage tracing and fluorescence-activated cell sorting, the dams were also homozygous for *r26r-lacZ* and *mTmG* alleles, respectively. Using *CRE*-positive, *Tfap2a*^+/null^; *Tfap2b*^+/null^ males previously used (Van Otterloo et al., 2018), we acquired *CRE*-negative controls (any *Tfap2a*/*Tfap2b* allelic combination) alongside littermates harboring any of the following *CRE*-positive genotypes: *Tfap2*^Δ/HET^ (i.e. *Tfap2a*^+/flox^; *Tfap2b*^+/flox^), *Tfap2a*^Δ/NCKO^ (i.e. *Tfap2a*^null/flox^; *Tfap2b*^+/flox^), *Tfap2b*^Δ/NCKO^ (i.e. *Tfap2a*^+/flox^; *Tfap2b*^null/flox^) and *Tfap2*^Δ/NCKO^ (i.e. *Tfap2a*^null/flox^; *Tfap2b*^null/flox^) (Fig. S1A,C). Embryos generated from this male genotype include ^Δ/NCKO^ in the shorthand nomenclature. Alternatively, using *Tfap2a*^+/flox^; *Tfap2b*^+/flox^ breeder males without the null allele, we acquired *CRE*-negative controls (any *Tfap2a*/*Tfap2b* allelic combination) alongside littermates harboring any of the following *CRE*-positive genotypes: *Tfap2*^HET^ (i.e. *Tfap2a*^+/flox^; *Tfap2b*^+/flox^), *Tfap2a*^NCKO^ (i.e. *Tfap2a*^flox/flox^; *Tfap2b*^+/flox^), *Tfap2b*^NCKO^ (i.e. *Tfap2a*^+/flox^; *Tfap2b*^flox/flox^) and *Tfap2*^NCKO^ (i.e. *Tfap2a*^flox/flox^; *Tfap2b*^flox/flox^) (Fig. S1B,C). For lineage tracing and scRNA-seq experiments, we used the *Tfap2*^HET^ condition for controls. A minimum of three independent biological replicates were produced for each experiment unless specified otherwise. For visualization of the breeding scheme and extended details on genotypes and nomenclature, see Fig. S1.

### Zebrafish alleles

The transgenic line *Tg(fli1a:EGFP)y1* (Lawson and Weinstein, 2002) and the germline mutant lines for *tfap2a low*^ts213^ and *alx3*^co3003^ have been previously reported (Knight et al., 2003; Mitchell et al., 2021).

### Scanning electron microscopy

Preparation of E11.5 *CRE*-negative control, *Tfap2*^HET^ and *Tfap2*^NCKO^ littermate heads and imaging was performed as previously described (Van Otterloo et al., 2022). Briefly, samples were fixed in 4% paraformaldehyde (PFA) and then rinsed in 1×PBS. Subsequently, samples were rinsed in deionized H_2_O, air-dried for at least 3 days, mounted on a scanning electron microscopy (SEM) stub, and sputter coated for 4.5 min at 5 mA using a gold/palladium target in an Emitech K550X sputter coater. Scanning electron micrographs were acquired using a Hitachi S4800 electron microscope operated in high-vacuum mode at 1.8 kV. Imaging was performed through the University of Iowa Central Microscopy Research Facility workflow.

### β-Galactosidase staining

E10.5 littermates with *Wnt1:CRE*-mediated recombination of the *r26r-lacZ* reporter allele were subject to β-galactosidase staining as previously described (Seberg et al., 2017). Embryos, microdissected from pharyngeal arch 2 and above, were gently rocked in 0.2% glutaraldehyde (in PBS) for 30 min at room temperature. After removing fixative, samples were washed thrice, for 30 min each, and then developed in a β-galactosidase staining solution comprising 1 mg/ml X-gal (Research Products International) in the dark at 37°C for ∼30-40 min. Once desired staining was acquired, samples were rinsed in 1×PBS at least twice and then fixed in 4% PFA overnight at 4°C, while protected from light.

### Tissue section analyses

#### Tissue sectioning

Sectioning of frozen tissue was performed as previously described (Van Otterloo et al., 2016), with minor modifications. In brief, E11.5 wild-type CD-1 mouse embryos or those of a mixed background were fixed overnight in 4% PFA at 4°C. Fixed embryos were then transferred through increasing concentrations of Tissue Tek OCT compound (Sakura Finetek USA), embedded in 100% OCT in the desired orientation, snap frozen on dry ice and stored at −80°C. Embryonic heads were sectioned in a horizontal fashion (10 µm slices) using a Microm HM525 NX Cryostat Microtome (ThermoFisher) housed within the University of Iowa Central Microscopy Research Facility, mounted onto positively charged glass slides and were left in a closed container at room temperature overnight to further adhere. We used the eMouseAtlas database (Armit et al., 2017) to identify the horizontal section angle.

#### Immunofluorescence

Tissue sections were blocked for 1.5 h in 5% milk or 3% BSA dissolved in 0.1% Triton-X/PBS (PBSTx). Wild-type sections were used to examine protein expression of TFAP2A (3B5-supernatant, Santa Cruz Biotechnology; 1:25 dilution) and TFAP2B (2509S, Cell Signaling Technology; 1:25 dilution). E11.5 control and *Tfap2*^NCKO^ midface sections were stained with phosphorylated Histone H3 (9701S, Cell Signaling Technology; 1:400 dilution) to assess cell proliferation. After washing with PBSTx, samples were incubated with Alexa Fluor 488/568 secondary antibodies (A-11017, A-11070 and A-21069, Invitrogen; 1:500 dilution) for 1.5 h. We used DAPI to counterstain nuclei. Stains were imaged via Zen software on a Zeiss 700 LSM confocal microscope. Images were exported as Z-stacked, maximum intensity projection TIFF images.

#### Cell proliferation analysis

pHH3-positive cells were manually counted in a broad region in the medial or lateral domains of the frontonasal prominence and normalized to manually counted DAPI-stained cells in the same region. Ectoderm tissue proliferation and autofluorescence from blood cells were not considered in the quantification. An unpaired Student's *t*-test was performed on GraphPad Prism to determine cell proliferation changes between controls and *Tfap2*^NCKO^ conditions, with *P*<0.05 being considered statistically significant.

#### Cell death analysis

Terminal deoxynucleotidyl transferase dUTP nick-end labeling (TUNEL) on frozen tissue sections was performed using the DeadEnd Fluorometric System (Promega) as previously described (Cui and Li, 2013), with modifications. All steps were performed at room temperature, in the dark from labelling step onward. 5-10 min washes were performed after each step using 1×PBS. Sections were fixed with 4% PFA for 10 min and then permeabilized in PBSTx for 5 min. To generate a positive control that reads out the success of the TUNEL assay, permeabilized tissue samples were subsequently incubated with DNase I (10 units/ml) for 5 min and washed twice. Note that positive controls were physically separated from other samples to prevent contamination of any residual DNase I. Next, labelling reactions were performed at 37°C for 1 h in a humidified chamber and quenched thereafter in 2×SSC for 15 min. Finally, tissue sections were counterstained with DAPI, embedded in mounting solution and imaged on a Nikon Eclipse 80i fluorescent microscope. Fluorescent particles smaller than nuclei are artifacts.

### Micro-computed tomography

The University of Iowa Small Animal Imaging Core Facility provided scanning services and software. Control-*Tfap2*^NCKO^ littermates were prepared as previously described (Hsu et al., 2016). In brief, embryos were fixed in 4% PFA at 4°C (overnight for E12.5, 3-7 days for E18.5), washed thrice and stored in 1×PBS until the day of scanning was determined. For E18.5 embryos, after fixation, hydrogel-based stabilization (Wong et al., 2013) was implemented to minimize tissue shrinkage. Before scanning, embryos were incubated in 1 N (v/v) iodine solution (Sigma) at 4°C (3 days for E12.5, 7 days for E18.5). Samples were then held in place by 1% agarose in a 15 ml conical tube and the remainder of the tube was gently flushed with 1×PBS, and immediately imaged on a Zeiss Xradia 520 Versa system. E12.5 samples were scanned at 5 or 6 µm resolution, whereas E18.5 samples were scanned at 11 µm resolution.

Three-dimensional reconstruction, sectional analysis and measurements of the µCT scans were performed using Dragonfly software (Object Research Systems). E12.5 embryo scans were oriented in a (1) frontal plane to examine nasal morphologies or (2) horizontal fashion, spanning from the nasal pits to the hindbrain to quantify the distance between the anterior tips of the medial domain of the frontonasal prominence. To quantify the length of the E18.5 snouts, linear measurements were taken in the horizontal plane from the anterior tip of the nares to the back of the head. The forelimb stylopod, measured from tendon to tendon, was used as an internal control as its development should not be affected by our genetic perturbations. Images for control-mutant pairs were displayed using similar contrast parameters. An unpaired Student's *t*-test was performed on GraphPad Prism, with *P*<0.05 being considered statistically significant.

### Concurrent Alizarin Red and Alcian Blue staining preparation

Concurrent Alizarin Red/Alcian Blue staining of E18.5 embryos and E15.5 Alcian Blue staining were performed as previously described (Van Otterloo et al., 2016). We did not examine nasal capsules in detail because staining was highly variable.

### Bulk RNA-sequencing

#### Tissue preparation, cDNA library generation and sequencing

E10.5 bulk frontonasal and maxillary prominence tissue from three control and three *Tfap2*^Δ/NCKO^ littermates were processed for RNA-seq as previously described (Van Otterloo et al., 2016). After PCR-genotyping, samples stored in RNAlater (ThermoFisher) were processed for RNA (Norgen Biotek) and further purified with the RNAeasy kit (Qiagen). mRNA quality was determined with DNA Analysis ScreenTape (Agilent Technologies), and cDNA libraries were generated using the Illumina TruSeq Stranded mRNA Sample Prep Kit (Illumina). Samples were sequenced on the Illumina HiSeq2500 platform as 150 bp single-end reads. Library construction and sequencing was carried out by the Genomics and Microarray Core on the University of Colorado Anschutz Medical Campus.

#### RNA-seq dataset processing and analyses

Following sequencing, reads were demultiplexed and FASTQ files processed using two distinct bioinformatic pipelines. Two pipelines were used to help reduce false positives. In the first approach, FASTQ files were mapped to the mm10 genome using STAR Aligner (Dobin et al., 2013) with default settings. Gene expression was then quantified using StringTie (Pertea et al., 2016, 2015), with the following settings: -e (expression estimation mode), -G (mm10 reference annotation file) and -A (gene abundances). After quantification, count matrices were produced using the *prepDE.py* script, available from StringTie, and used as input for DESeq2 (Love et al., 2014). Differential gene expression analysis was conducted in DESeq2 using the *DESeqDataSetFromMatrix* function. For the second approach, FASTQ files were pseudo-aligned to the mm10 genome using the kallisto (Bray et al., 2016) *quant* function with the following settings: -b 50, --single, -l 100 and -s 20. After alignment, differential gene expression analysis was conducted using sleuth (Pimentel et al., 2017), along with the *sleuth_prep* function and the following settings: full_model=genotype, gene_model=TRUE, read_bootstrap_tpm=TRUE, extra_bootstrap_summary=TRUE, transformation_function=function(x) log2(x+0.05). The resulting ‘sleuth object’ was then subjected to the *sleuth_fit* function, followed by the *sleuth_wt* function to calculate differentially expressed genes between genotypes. The kallisto-sleuth pipeline output was used, along with ggplot2 (Wickham, 2016) and ggrepel, to generate and visualize differentially expressed genes by volcano plot. Genes that had both (1) a fold-change greater or less than +1.25 or −1.25, respectively, and an adjusted *P*-value less than 0.05 (pipeline 1), and (2) a q-value less than 0.1 (pipeline 2) (Table S1) were used for ontology, enrichment and pathway analysis using the Enrichr platform (Chen et al., 2013; Kuleshov et al., 2016; Xie et al., 2021).

#### Re-analysis and visualization of published RNA-seq datasets

To directly compare the E11.5 mandibular and frontonasal prominence mesenchyme (e.g. Fig. 2), a procedure like ‘pipeline 2′ above (kallisto and sleuth) was used. Specifically, triplicate bam files were downloaded from the Facebase.org repository (FB00000867) for both E11.5 mandibular prominence mesenchyme (Biosample 30H6; RID 33SA, 33SE and 33SJ) and E11.5 frontonasal prominence mesenchyme (Biosample 2Y6P; RID: 33TT, 33TY and 33V2). Next, bam files were converted to FASTQ files using the *bam2fq* function in samtools (Li et al., 2009), Generated FASTQ files were then used along with kallisto and sleuth for differential gene expression analysis. Data were plotted using ggplot2 and ggrepel.

### Mouse single-cell RNA-sequencing

#### Single-cell dissociation, cell sorting and sequencing

To isolate CNCCs, E11.5 embryonic heads were cut immediately below pharyngeal arch 2 and the otic vesicle by microdissection. Using *Wnt1:CRE*-mediated recombination of the *mTmG* reporter allele (Muzumdar et al., 2007), *CRE*-positive embryos were selected for further processing. Tissue samples were then subjected to a cold protease single-cell dissociation protocol (Adam et al., 2017), with modifications. Embryo heads were incubated in a protease cocktail comprising *Bacillus licheniformis* Subtilisin A (Creative Enzymes), Accumax, and Accutase (Fisher Scientific) suspended in DEPC-PBS as previously described (Sekiguchi and Hauser, 2019). The tissue was then carefully disrupted using a disposable Eppendorf pellet pestle and placed on a rotator at 4°C for 30 min. During this step, samples were gently triturated every 10 min using a wide-bore 1 ml pipette tip. The enzymatic reaction was quenched using an equal volume of 10% heat-inactivated FBS (Fisher Scientific) in 1× Phenol Red-free DMEM (ThermoFisher). Dissociated cells were washed thrice in DEPC-PBS by centrifugation at 600 ***g*** at 4°C for 5 min and then transferred, through Bel Art SP Scienceware Flowmi 40 μm strainers (ThermoFisher), into chilled flow tubes pre-coated with DEPC-PBS. After GFP-positive sorting on the University of Iowa Flow Cytometry Facility's ARIA II system (Becton Dickinson), CNCCs were re-suspended in DEPC-PBS containing 0.04% non-acetylated BSA (New England Biolabs). Cell viability was determined to be >95% by Trypan Blue staining. During cell sorting, genotypes were confirmed by rapid genotyping, allowing isolation of GFP-positive cells from two *Tfap2*^HET^ control and two sibling-matched *Tfap2*^NCKO^ mutant embryos. Cells from each ‘biological replicate’ were combined to generate a single control and mutant sample.

Approximately 6000 GFP-positive CNCCs from each condition were subject to library preparation by the University of Iowa Genomics Division on the 3′ expression scRNA-seq 10X Chromium v3.1 pipeline. Libraries were sequenced on an Illumina NovaSeq 6000 platform as 100 bp paired-end reads. Over 20,000 reads per cell were acquired, resulting in ∼1.5×10^8^ reads per condition.

#### Quality control, integration, and UMAP visualization

Sequencing results were demultiplexed and converted to a FASTQ format using the Illumina bcl2fastq software. Reads were processed and aligned to the mm10 reference genome assembly with the Cell Ranger *Count* function. Seurat v4.3.0 was used for quality control, filtering out cells with >10% mitochondrial counts, less than 200 features and more than 7500 features. We then integrated *Tfap2*^HET^ and *Tfap2*^NCKO^ conditions (Hao et al., 2021), where RNA counts were normalized and *FindVariableFeatures* was run with the following parameters: selection.method=“vst”, nfeatures=2000. Cell clustering by Uniform Manifold Approximation and Projection (UMAP) plots was deployed by *RunPCA* and then *RunUMAP* (dims 1:30). The UMAP visualizing all CNCCs was generated with *FindClusters* using a resolution of 0.40. Major CNCC derivatives were annotated based on published immunofluorescence and scRNA-seq profiling (Soldatov et al., 2019). Gene expression was plotted on UMAP plots by *FeaturePlot*. Gene expression mapping of CNCC lineage markers was done using the *FeaturePlot* function.

#### Gene expression-based cell cycle analysis

The *CellCycleScoring* function was performed as previously described, with minor modifications (Hao et al., 2021). The UMAP resolution was changed to 0.05 to segregate the major CNCC lineages as individual clusters (i.e. mesenchyme, neuron and glia). Bioconductor BiomaRt v2.46.3 (Durinck et al., 2005) was used to convert a published list of cell cycle genes (Kowalczyk et al., 2015) from human to mouse gene nomenclature. Cell cycle scores were then mapped directly onto the UMAP, while GraphPad Prism was used to graph the scores as a percentage relative to each cluster.

#### Cellular distribution analysis

To examine genotype cellular distributions in each cluster, each condition was expressed as a percentage per cluster. These analyses were visualized in GraphPad Prism. For the initial UMAP containing all sorted cells, a resolution of 0.05 was used. For the MAGIC-clustered UMAP, a resolution of 0.10 was used (see the section ‘Annotation of mesenchyme positional identities’).

#### Annotation of mesenchyme positional identities

To better define the facial prominence subpopulations, we subset and re-clustered the ‘Mesenchyme’ clusters using MAGIC v2.0.3 (van Dijk et al., 2018) with *RunUMAP* dims 1:20. Major mesenchyme populations were then annotated based on known gene markers, which were plotted on the MAGIC-clustered UMAP with DefaultAssay=“MAGIC_RNA”. A cluster resolution of 0.10 was chosen to best visualize each prominence population and the second pharyngeal arch as individual clusters based on published *in situ* data, ChIP-seq, ATAC-seq and transcriptomic datasets (Gu et al., 2022; Hooper et al., 2020; Minoux et al., 2017). To visualize co-expression of two genes on the UMAP, we used *FeaturePlot* (flag *blend=“TRUE”*).

#### Gene-set and gene expression analyses

The UMAP resolution was set to 0.05 to distinguish the major cell groupings, to which we then subset the ‘pseudobulked’ mesenchyme group. We then used the *FindMarkers* function with ident.1=*Tfap2*^HET^ and ident.2=*Tfap2*^NCKO^ on CNCCs, to generate a list of differentially expressed genes (adjusted *P*-value<0.05 and a log fold-change threshold of 0.10) (Table S2). For gene-set enrichment, ontology and pathway analysis, lists of either up- or downregulated genes, generated from the mesenchyme ‘pseudobulk’ approach, in the *Tfap2*^NCKO^ condition were inputted in the Enrichr pipeline. Control-versus-mutant expression analysis of select genes was visualized as a violin plot using the *VlnPlot* function on the MAGIC-clustered UMAP, with DefaultAssay=“RNA”. Differential gene expression was validated for a select set of genes of interest by targeted approaches (see real-time PCR).

#### Gene-set overlap with bulk RNA-seq datasets

First, to determine overlapping genes between the E11.5 scRNA-seq and E10.5 bulk RNA-seq datasets, gene lists of differentially expressed genes were compiled (Table S1). Datasets used included the following outputs: E11.5 scRNA-seq mesenchyme ‘pseudobulk’ (Table S1), E10.5 bulk frontonasal/maxillary prominence (i.e. ‘upper-face’) pipeline 1 (Table S1) and pipeline 2 (Table S1), and E10.5 bulk MdP (i.e. ‘lower-face’) pipeline 1 and pipeline 2 (Van Otterloo et al., 2018). Second, the VLOOKUP function in Excel was used to intersect all four E10.5 gene lists with the E11.5 pseudobulk gene list and generate a comprehensive overlap table (Table S1).

### Real-time PCR

E10.5 bulk frontonasal prominence tissue, divided by medial and lateral domains, was acquired from *CRE*-negative controls and *Tfap2*^Δ/NCKO^ mutants by micro-dissection. Similarly, E11.5 *Tfap2*^HET^ and *Tfap2*^NCKO^ CNCCs were collected from frontonasal and maxillary prominence tissue by the *Wnt1:CRE-mTmG* sorting strategy. For the latter, single-cell dissociation was conducted using 0.25% trypsin (ThermoFisher) for 15 min at 37°C. Samples were either stored in RNAlater (ThermoFisher) at −20°C or immediately processed for RNA using the RNeasy Mini Kit as per the manufacturer's protocol (Qiagen). Real-time analyses were performed as previously described (Van Otterloo et al., 2018). After PCR-genotyping, cDNA was prepared with SuperScript III First-Strand Synthesis Kit (Invitrogen/ThermoFisher), where equal amounts of RNA template were processed from each condition. Real-time reactions were performed on a CFX Connect instrument (Bio-Rad) with either SYBR Green or Select Master Mixes (Applied Biosystems, ThermoFisher). Primers targeting *Alx1*, *Alx3* and *Alx4* transcripts were designed to target exons flanking intronic sequences. Relative mRNA expression levels were normalized to *B2m* or *Actb* transcripts. Real-time PCR primers are listed in Table S5.

### Whole-mount *in situ* hybridization

Control and *Tfap2*^Δ/NCKO^ embryos at E10.5 were subjected to whole-mount *in situ* hybridization with *Alx3* and *Alx4* RNA probes as previously described (Simmons et al., 2014). Primers used to generate the probes are listed in Table S5.

### *In vitro* transcription/translation followed by western blotting

*In vitro* transcription/translation followed by western blotting was performed as previously described (Li et al., 2013). In brief, 1 µg of T7βSal plasmids, containing cDNA for TFAP2A, TFAP2B, TFAP2C or TFAP2E, were used for TNT T7 Quick Coupled Transcription/Translation System (Promega) reactions. After a 90 min incubation at 30°C, reactions were terminated with a 10 min incubation with RNase A at 30°C. 2 µl of each reaction mixture was loaded on a 10% SDS-PAGE gel and followed by western blotting. Anti-TFAP2A (sc-184, Santa Cruz Biotechnology; 1:2000 dilution) was used as the primary antibody, with anti-rabbit IgG (NA943, Amersham Bioscience; 1:50,000 dilution) used as the secondary antibody. Signal detection was performed via Western Lightning Plus Enhanced Chemiluminescence Substrate (PerkinElmer).

### Chromatin immunoprecipitation followed by sequencing

#### Tissue preparation, library construction and sequencing

Chromatin immunoprecipitation (ChIP) was conducted, essentially as previously described (Van Otterloo et al., 2022), with the exception that full facial prominences were used, rather than only isolated ectoderm. Further modifications included the use of an anti-TFAP2A antibody (5 µg of sc-184, Santa Cruz Biotechnology) and Dynabeads (ThermoFisher Scientific). Following precipitation and subsequent quality control analysis (ScreenTape analysis, Agilent), samples (i.e. sc-184 IP and input DNA) were sequenced on the Illumina HiSeq 2500 platform as 150 bp single-end reads. Library construction and sequencing was carried out by the Genomics and Microarray Core at the University of Colorado Anschutz Medical Campus.

#### Peak calling and analyses

After sequencing and demultiplexing, FASTQ files were mapped to the mm10 genome using BWA (Li and Durbin, 2009), followed by sam to bam file conversion using Samtools (Li et al., 2009). To identify ‘enriched’ regions (i.e. regions with significantly more reads in the TFAP2 IP versus input), MACS2 (Zhang et al., 2008) was used with the following settings: -f BAM, -g mm, --keep-dup 2 (Table S2). Motif and peak annotation were completed using the *findMotifsGenome.pl* and the *annotatePeaks.pl* functions in HOMER (Heinz et al., 2010). Heatmap visualization of enriched regions was completed using the *bamCoverage*, *computeMatrix* and *plotHeatmap* function in deepTools (Ramirez et al., 2016), with the following settings: -of ‘bigwig’, --ignoreDuplicates, -bs 25, --normalizeUsing RPKM (for *bamCoverage*); reference-point, -b 3000, -a 3000 –referencePoint center (for *computeMatrix*); --whatToShow ‘heatmap and colorbar’, --missingDataColor 1, --kmeans 7, --refPointLabel “peak” (for *plotHeatmap*). For *computeMatrix* (Fig. 5E), the following scaled bigwig datasets were downloaded from GEO (Barrett et al., 2013) and used (along with the MACS2-generated ‘TFAP2-bound’ bed file): GSM2371708, E10.5 frontonasal prominence H3K4me2; GSM2371717, E10.5 frontonasal prominence H3K27ac; GSM2371732, E10.5 frontonasal prominence ATAC-seq; GSM2371713, E10.5 frontonasal prominence H3K27me3 (peak-cluster annotations in Table S2) (Minoux et al., 2017). Additional datasets used for analysis included: GSM2371710, E10.5 mandibular prominence H3K4me2; GSM2371719, E10.5 mandibular prominence H3K27ac; GSM2371734, E10.5 mandibular prominence ATAC-seq; GSM2371715, E10.5 mandibular prominence H3K27me3. The Integrative Genomics Viewer (Robinson et al., 2011) was used for gene level visualization of these datasets at *Alx1/3/4* loci. Pathway analysis was conducted using GREAT (McLean et al., 2010) with the following setting: ‘*Basal plus extension*’, ‘Proximal 5 kb upstream, 1 kb downstream, plus Distal: up to 100 kb’.

To intersect peak coordinates with dysregulated genes, a data matrix was generated (Table S2) that included all genes in the GREAT database (mm10, *n*=20,510), their TFAP2 peak association (yes/no) using the criteria outlined above, along with gene lists from the E10.5 bulk RNA-seq analysis and the E11.5 mesenchyme ‘pseudobulk’ analysis. The E10.5 bulk RNA-seq datasets included the ‘upper-face’ pipeline 1, pipeline 2 and their overlap (this study) along with the ‘lower-face’ pipeline 1, pipeline 2 and their overlap (Van Otterloo et al., 2018). Total genes with or without peaks (excluding those that did not have a 1:1 match between datasets) were summed and summarized (Table S2). To correlate peak status with gene expression in the midface, genes with and without an associated TFAP2 peak were paired with previously calculated FPKM values from the E11.5 frontonasal prominence mesenchyme (Hooper et al., 2020) (Table S2). Data were plotted and visualized using ggplot2 and statistical significance calculated using ggpubr.

### Cleavage under targets and release using nuclease (CUT&RUN) followed by sequencing

#### Sample preparation, sequencing and peak calling

Cleavage under targets and release using nuclease (CUT&RUN) was performed as previously described (Kenny et al., 2022; Meers et al., 2019; Skene and Henikoff, 2017), with modifications to include *in vivo* single-cell dissociation. In summary, 24 h post-fertilizaiton zebrafish embryos were dissociated in calcium-free wash buffer [20 mM HEPES (pH 7.5), 150 mM NaCl, 0.5 mM spermidine and Roche cOmplete Mini, EDTA-free protease inhibitor cocktail], centrifuged at 300 ***g*** and washed twice in wash buffer. Cells were bound to activated Concanavalin A beads (Bangs Laboratories) and incubated in antibody overnight at 4°C. Herein, we used anti-Tfap2a (GTX128757, GeneTex; 1:100) and anti-rabbit IgG (EMD Millipore, 12-370, 1:213). Cells were then incubated in pAG-MNase (700 µg/ml), the cleavage reactions initiated with the addition of CaCl_2_ and quenched after 30 min in a stop buffer containing 2 pg/ml sonicated yeast spike-in control. DNA fragments were then released at 37°C for 30 min, extracted via phenyl/chloroform and precipitated with ethanol. DNA concentrations were quantified using a Qubit fluorometer (ThermoFisher), and fragment sizes were analyzed using a 2100 Bioanalyzer (Agilent). CUT&RUN libraries were prepared using the KAPA Hyper Prep Kit (Roche), pooled, brought to equimolar concentrations and sequenced on the NovaSeq platform to obtain 25 million reads per sample. After sequencing demultiplexing of sequencing data, as previously described (Kenny et al., 2022), paired-end FASTQ files were processed using FastQC (Babraham Bioinformatics). Reads were trimmed using Trim Galore v0.6.3 (Felix Kruger, Babraham Institute, Cambridge, UK), mapped against the danRer11 genome assembly via Bowtie2 v2.1.0 (Langmead and Salzberg, 2012), and peak called against their respective IgG samples using MACS2 (Zhang et al., 2008).

#### Integration of CUT&RUN with re-analyzed zebrafish CNCC single-nuclei ATAC-seq datasets

FASTQ files (SRR14924014, SRR14924015, SRR14924016 and SRR14924017) were downloaded from GEO (Barrett et al., 2013) using the SRA-Toolkit v2.11.0 *fastq-dump* function (flags --split files, --gzip). Before FASTQ genome mapping, a zebrafish reference genome was generated using the Danio rerio GRCz11 primary assembly (Ensemble, release 110), a filtered GRCz11.110 gtf file was generated using the *mkgtf* function in Cell Ranger ATAC v2.1.0 (10X Genomics) (flag --attribute=gene_biotype:protein_coding) and a transcription factor motif file in JASPAR format (2022 CORE non-redundant pfms) (Castro-Mondragon et al., 2022) was used for motif identification. Subsequently, FASTQ files were mapped to the GRCz11 genome using the count function in Cell Ranger ATAC, along with the assembled reference genome and default settings. Generated ‘count’ files were then aggregated into a single file using the Cell Ranger ATAC *aggr* function, with default settings.

The generated aggregated count output was next loaded into Signac v1.10.0 (Stuart et al., 2021). Briefly, steps included reading in the filtered peak matrix file (e.g. filtered_peak_bc_matrix.h5) and an associated metadata file (e.g. singlecell.csv). Next, the *CreateChromatinAssay* function (min.cells=10, min.features=200) in Signac was used, along with the ‘filtered peak matrix file’ and a ‘fragments file’ (generated by the *count* function in Cell Ranger ATAC) to generate a ‘Chromatin Assay’ file. Next, a ‘Seurat Object’ was generated using the *CreateSeuratObject* function, along with the ‘Chromatin Assay file’ and the associated metadata file. Genes within the Seurat Object were annotated using the GRCz11.110 gft file. The Seurat Object was subsequently subset (nCount_peaks>3000 & nCount_peaks<30000 & pct_reads_in_peaks>15 & blacklist_ratio<0.05 & nucleosome_signal<4 & TSS.enrichment>3) and processed using the following functions: *RunTFIDF*, *FindTopFeatures* (min.cutoff=‘q0’), *RunSVD*, *RunUMAP* (reduction=‘lsi’, dims=2:30), *FindNeighbors* (reduction=‘lsi’, dims=2:30) and *FindClusters* (algorithm=3, resolution=0.08), resulting in the generation of 11 clusters. Two approaches were used to identify clusters. First, the *GeneActivity* function (default settings) was used to infer gene expression levels based on local chromatin accessibility and values added as a new assay in the Seurat Object using the *CreateAssayObject* function. Values were normalized using the *NormalizeData* function (normalization.method=‘LogNormalize’, scale.factor=median) and visualized using the *FeaturePlot* function (max.cutoff=‘q95’). Second, differentially accessible peaks between clusters were identified using the *FindMarkers* function (only.pos=TRUE, test.use=‘LR’, min.pct=0.05) in Signac. The top 1000 peaks for each cluster (after removing unannotated contigs) were then used for motif enrichment analysis using the *FindMotifs* function with default settings. Using a combination of ‘Gene activity’ and ‘Motif enrichment’, we subsequently inferred ectomesenchyme and ‘upper-face’ clusters.

Finally, CUT&RUN bed files were imported using the *import.bed* function of rtracklayer v1.58.0 (Lawrence et al., 2009), with *alx1*, *alx3* and *alx4a* loci visualized using the *CoveragePlot* function in Signac. Given the absence of *alx3* from the current zebrafish genome build, a separate bed file demarcating the four *alx3* exons (i.e. chr8 24796486 24800056, chr8 24801510 24801635, chr8 24802326 24802600 and chr8 24807613 24808038) was also imported and used to generate a ‘gene track’ for *alx3*.

### Zebrafish single-cell RNA sequencing analysis

Expression mapping on UMAPs of published zebrafish CNCC scRNA-seq datasets at 24 h post-fertilization (GEO accession number GSE220081) were performed as previously described (Stenzel et al., 2022).

### *In situ* hybridization chain reaction

We ordered oligos from Molecular Instruments using the XM_005167111.4 *alx3* transcript and targeting the same 649-nucleotide 5′ UTR sequence previously used for *in situ* hybridization (Mitchell et al., 2021). We then followed the manufacturer's protocol for whole-mount zebrafish embryos and larvae using the B2 amplifier (Choi et al., 2016) and probe set size of 20. High salt content in buffers used during the hybridization chain reaction (HCR) v3.0 protocol made genotyping difficult. To circumvent this, we used Promega GoTaq Flexi (M8295) in ‘M’ buffer [2 mM MgCl_2_, 14 mM Tris-HCl (pH 8.4), 68.25 mM KCl, 0.0013% gelatin, 1.8 mg/ml BSA and 140 µM each dNTP]. Zebrafish embryos were then embedded in 0.2% agarose and imaged with an Andor Dragonfly 301 spinning disk confocal system. Acquisition parameters and fluorescence adjustments were applied linearly and equally to all samples.

Digital mRNA absolute quantitation (Table S3) was performed using dot detection methods (Choi et al., 2018). For each embryo, the total numbers of transcripts (spots) were captured from three regions: the midline (area between the eyes, below the nares and above the mouth); the lateral region (corresponding to the area outside of the midline region under the nares at the same level of the mouth); and the medial nares (corresponding to the medial edge of both nares along the dorsoventral axis) (see Fig. 6C). Comparison of means from five embryos in each genotype was carried out using a Brown-Forsythe ANOVA test coupled with Dunnett's T3 test for multiple comparisons using GraphPad Prism.

### Genetic interaction analyses

Zebrafish larvae 6 days post-fertilization were subject to concurrent Alizarin Red/Alcian Blue staining preparations as previously described (Walker and Kimmel, 2007). Nomarski imaging, genotype-blinded phenotype scoring and statistical analysis were performed as previously described (Bailon-Zambrano et al., 2022; Mitchell et al., 2021; Sucharov et al., 2019). In brief, larval skeletons were dissected and flat mounted for imaging on a Leica DMi8 inverted microscope equipped with a Leica DMC2900. For penetrance scores, we performed the Fisher's exact test using GraphPad (Table S4). All sample sizes, raw scoring and *P*-values are included in Table S4.

## Supplementary Material

10.1242/develop.202095_sup1 Supplementary information

Table S1.

Table S2.

Table S3.

Table S5.

Table S6.
