# Supplementary material for: TFAP2 paralogs regulate midfacial development in part through a conserved ALX genetic pathway
Source: Development. 2024 Jan 2;151(1):dev202095. doi: 10.1242/dev.202095 (PMC10820886; doi:10.1242/dev.202095)
Supplement: Supplementary information [file develop-151-202095-s1.pdf]

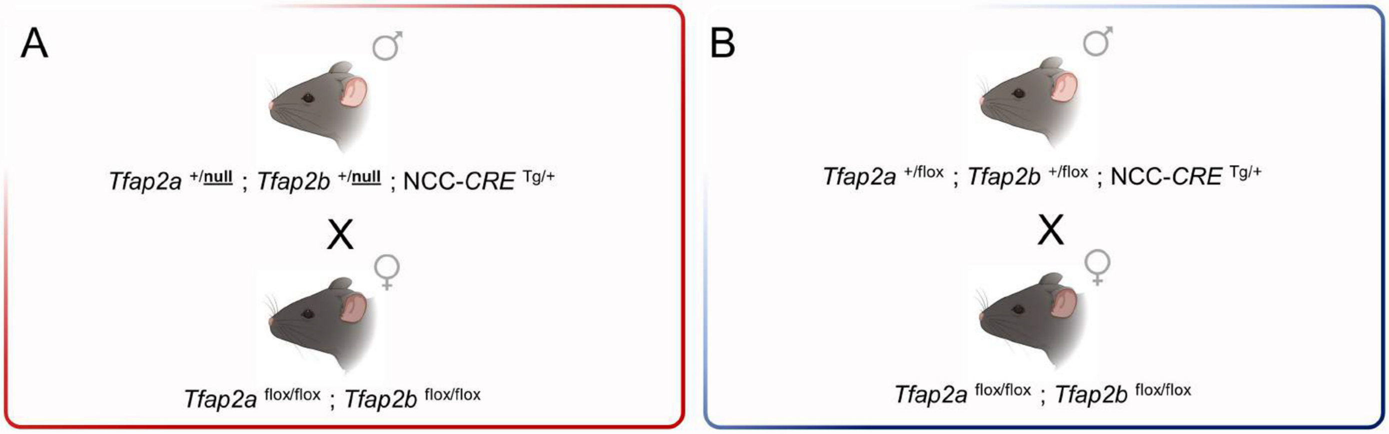

C

	Nomenclature in this Study	*Tfap2a*	*Tfap2b*	NCC-*CRE*	Previous Nomenclature (Van Otterloo et al., 2018)
null/flox	CTRL	any combination		negative	CTRL
	Tfap2$^{\Delta/HET}$	+ / flox	+ / flox	positive	
	Tfap2a$^{\Delta/NCKO}$	**null** / flox	+ / flox		SCM-A
	Tfap2b$^{\Delta/NCKO}$	+ / flox	**null** / flox		SCM-B
	Tfap2$^{\Delta/NCKO}$	**null** / flox	**null** / flox		DCM
flox/flox	CTRL	any combination		negative	not applicable
	Tfap2^{HET}	+ / flox	+ / flox	positive	
	Tfap2a^{NCKO}	flox / flox	+ / flox		
	Tfap2b^{NCKO}	+ / flox	flox / flox		
	Tfap2^{NCKO}	flox / flox	flox / flox		

Fig. S1. Mouse genetic approaches to investigate TFAP2A and TFAP2B function in CNCCs during midfacial development. (**A, B**) Graphics depicting the null/flox breeding scheme (A), previously described (Van Otterloo et al., 2018), and the flox/flox breeding scheme (B) used primarily in this study. Note that while females used between the two schemes are identical, the difference lies in the sire's genotype. Cartoons are adapted from BioRender. (**C**) Table summarizing key *Tfap2a* and *Tfap2b* allelic combinations obtained using the breeding scheme highlighted in panels A or B, along with current and previous shorthand nomenclature. Note, mice acquired from the sire harboring the null alleles are denoted with a "Δ/NCKO" superscript in their shorthand nomenclature. Abbreviation: NCC, neural crest cell.

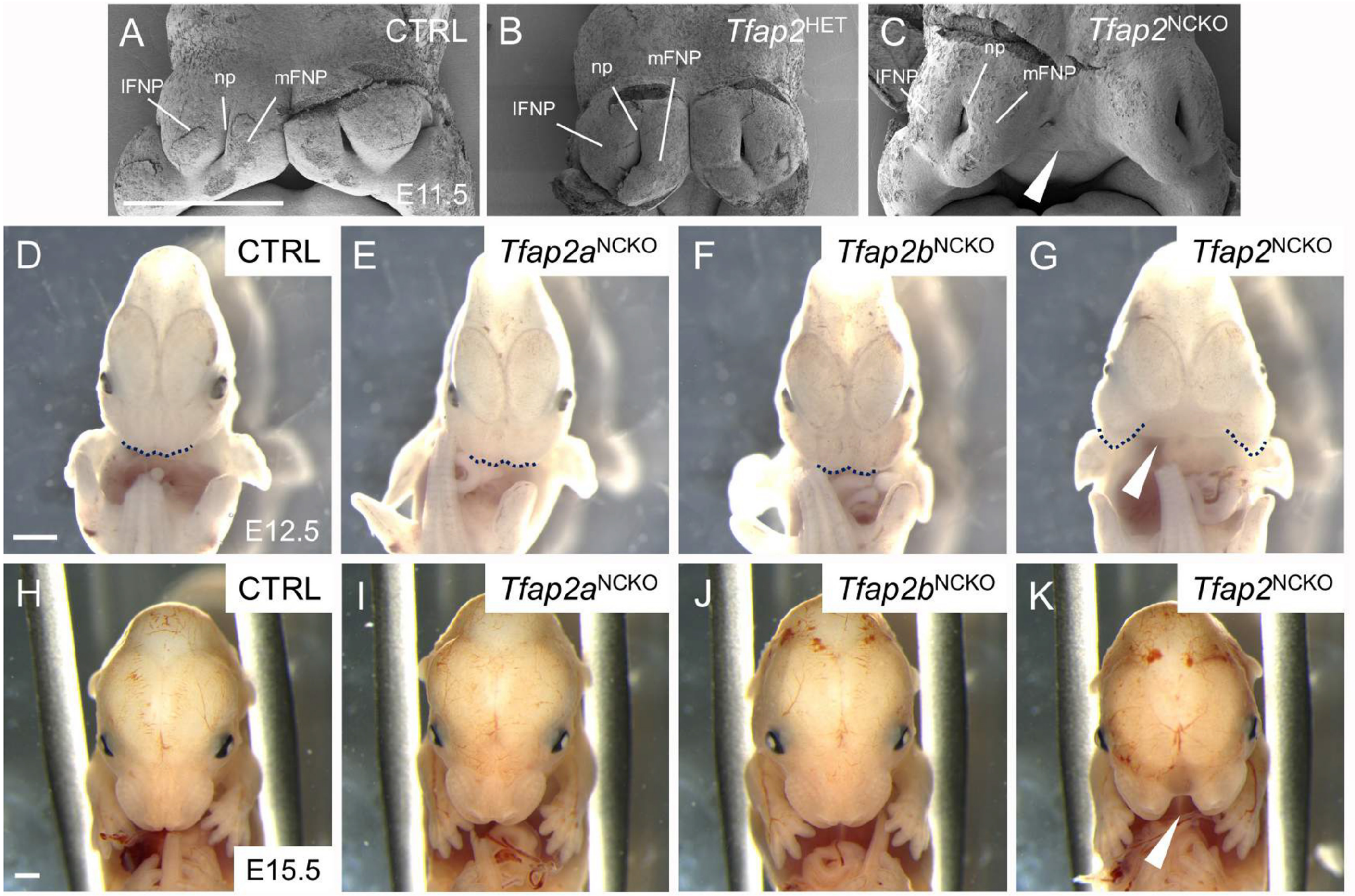

Fig. S2. Midfacial clefting arises from combined loss of *Tfap2a* and *Tfap2b* in CNCCs. (**A-K**) Gross craniofacial morphology, as detected by scanning electron microscopy (A-C) or brightfield imaging (B-K) of E11.5 (A-C), E12.5 (D-G), or E15.5 (H-K) embryonic midfaces, as indicated by genotype. Panels A-C is ventral views, whereas panels D-K are top-down views of the cranium and midface. Black dotted lines in the E12.5 images highlight the frontonasal prominences. White arrowheads point to the midface cleft. N = 3 per genotype. Scale bar = 1 mm. Abbreviations: FNP, frontonasal prominence; lFNP, lateral FNP; mFNP, medial FNP; np, nasal pit.

Development • Supplementary information

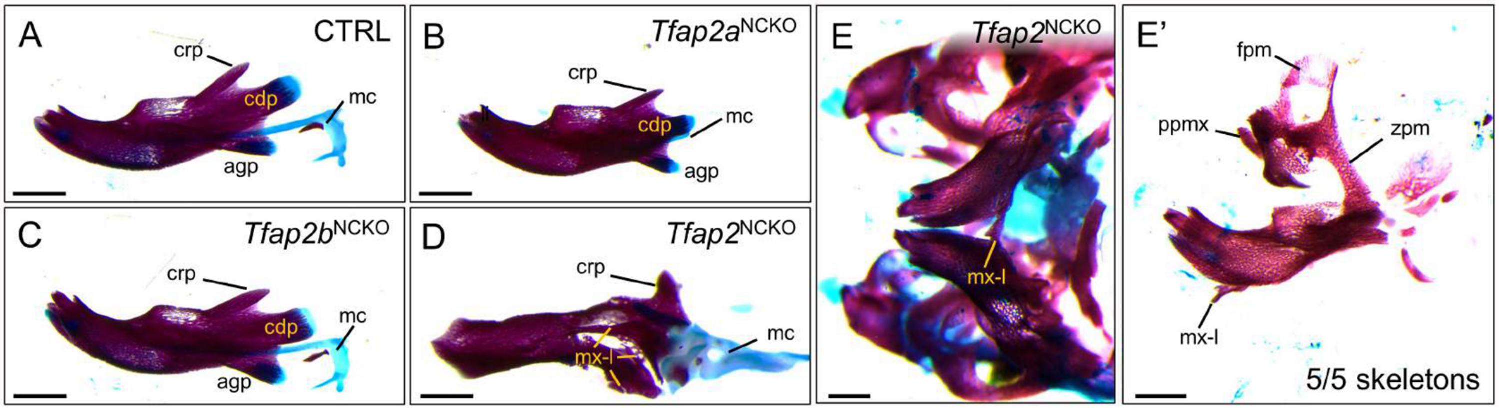

Fig. S3. *Tfap2*$^{\mathbf{NCKO}}$ mutant skeletal phenotypes recapitulate those observed in *Tfap2*$^{\mathbf{\Delta/NCKO}}$ mutants. (**A-D**) Isolated left mandible from E18.5 *Tfap2*^{NCKO} embryos, with indicated genotypes. These phenotypes match those previously described in null/flox embryos (Fig. S1) (Van Otterloo et al., 2018). Scale bar = 1 mm. N = 5 per genotype. **(E)** Inferior view of an E18.5 *Tfap2*^{NCKO} skeletal preparation showing 'maxilla-like' medial projections on the mandible that meet at the midline (left) along with (**E'**) syngnathia of the left jaw viewed laterally in isolation. Note that the isolated mandible in Panel D was also fused to the maxilla but isolated for imaging. Abbreviations: agp, angular process; cdp, condylar process; crp, coronoid process; fpm, frontal process of the maxilla; mc, Meckel's cartilage; mx-l, maxilla-like; ppmx, palatal process of the maxilla; zpm, zygomatic process of the maxilla.

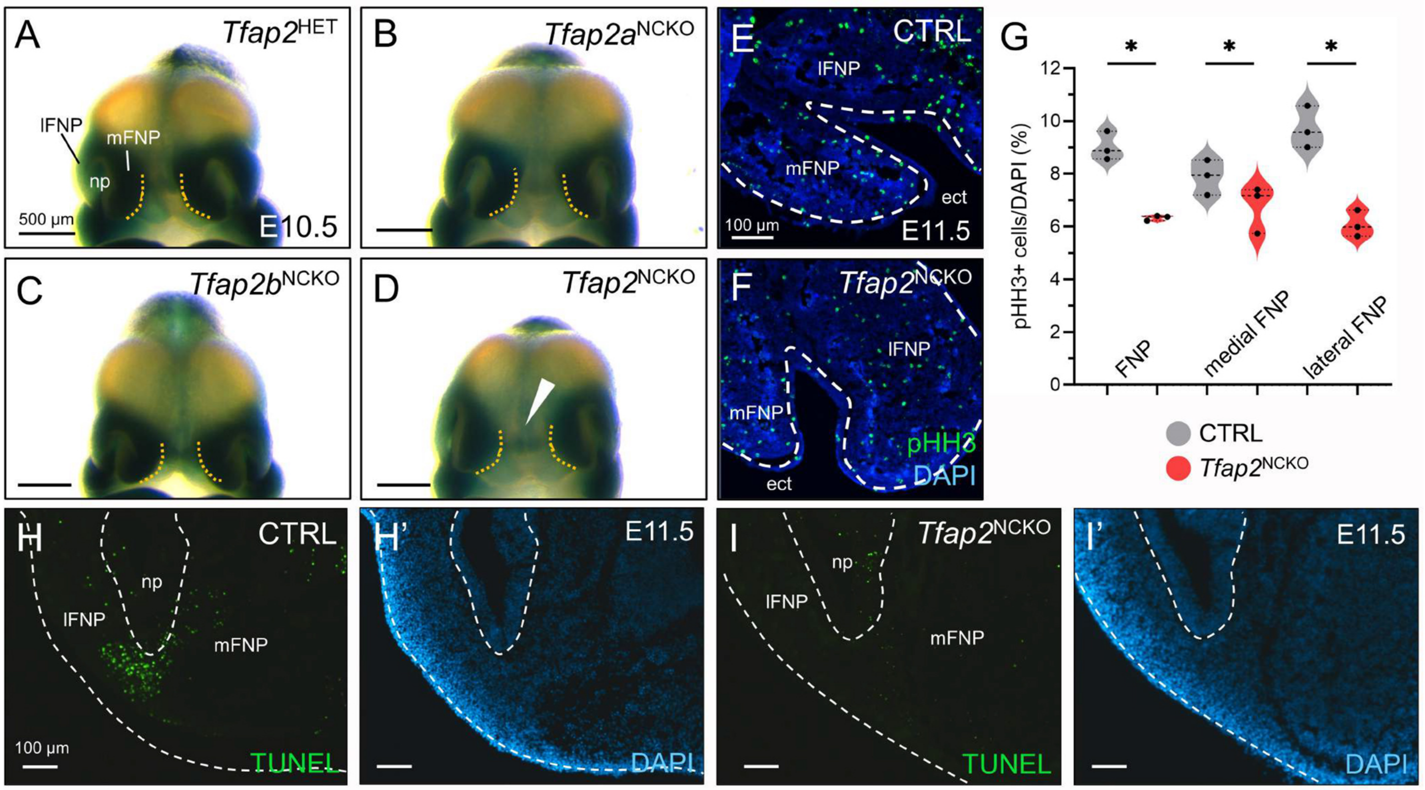

Fig. S4. Cellular analyses suggest a post-migratory role for TFAP2A and TFAP2B during midfacial development. (**A-D**) Front view of E10.5 embryos, with indicated genotypes. Cranial neural crest cells are β-galactosidase-stained from *Wnt1:CRE*-mediated recombination of the *r26r-lacZ* reporter allele. Dashed yellow lines mark the midline-proximal edges of the medial domains of the frontonasal prominences (mFNP). White arrowhead in panel D points to the increased gap between the mFNP. (**E, F**) Phospho-Histone H3 (pHH3) staining and DAPI nuclei counterstaining in E11.5 control (E) and mutant (F) conditions. White dashed lines indicate boundaries between mesenchyme and ectoderm. (**G**) Percentage of pHH3-positive cells, counted from tissue sections like those from panels E and F, normalized by DAPI staining. Percentages were calculated from sections for the whole FNP, mFNP alone, and lateral domain of the FNP (lFNP) alone. N = 3 per genotype. Student's t-test, $^*p < 0.05$. (**H, I**) TUNEL staining (H, I) and DAPI nuclei counterstaining (H', I') in E11.5 control (H) and mutant (I) conditions. Note that fluorescent puncta are smaller than nuclei and considered artifacts. Additional abbreviations: d, dorsal; ect, ectoderm; v, ventral.

Development • Supplementary information

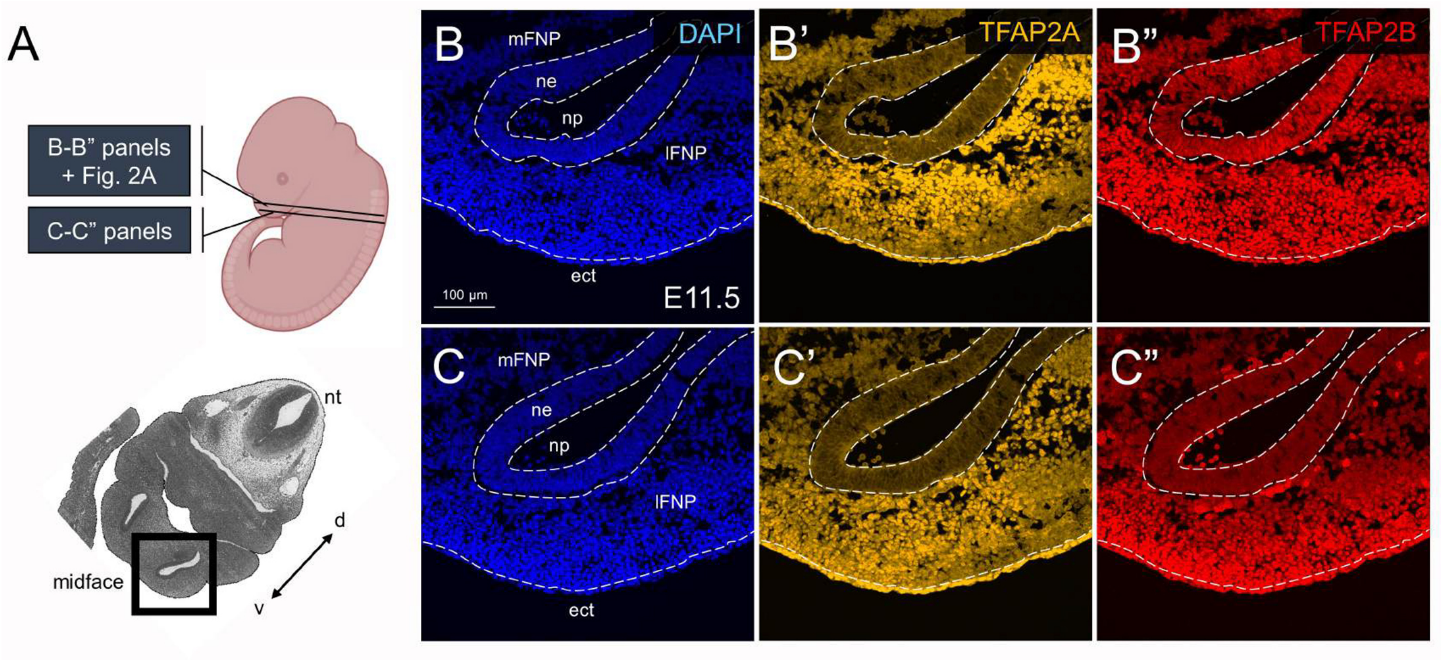

Fig. S5. TFAP2A and TFAP2B overlapping expression domains during midfacial development. (**A**) The top image, derived from BioRender, indicates the plane of each section that wild-type TFAP2 co-staining was performed in panels B and C as well as Fig. 2A. The bottom-left image, derived from the eMouse atlas (Armit et al., 2017), is a representative orientation of the tissue section. Boxed is the region examined in the immunofluorescent images. (**B-B", C-C"**) Co-staining of DAPI (B, C), TFAP2A (B', C'), and TFAP2B (B", C") in wild-type midface tissue in two different planes. White dashed lines indicate boundaries between mesenchyme and epithelium. Additional abbreviations: d, dorsal; ect, ectoderm; ne, nasal epithelium; np, nasal pit; v, ventral.

Development • Supplementary information

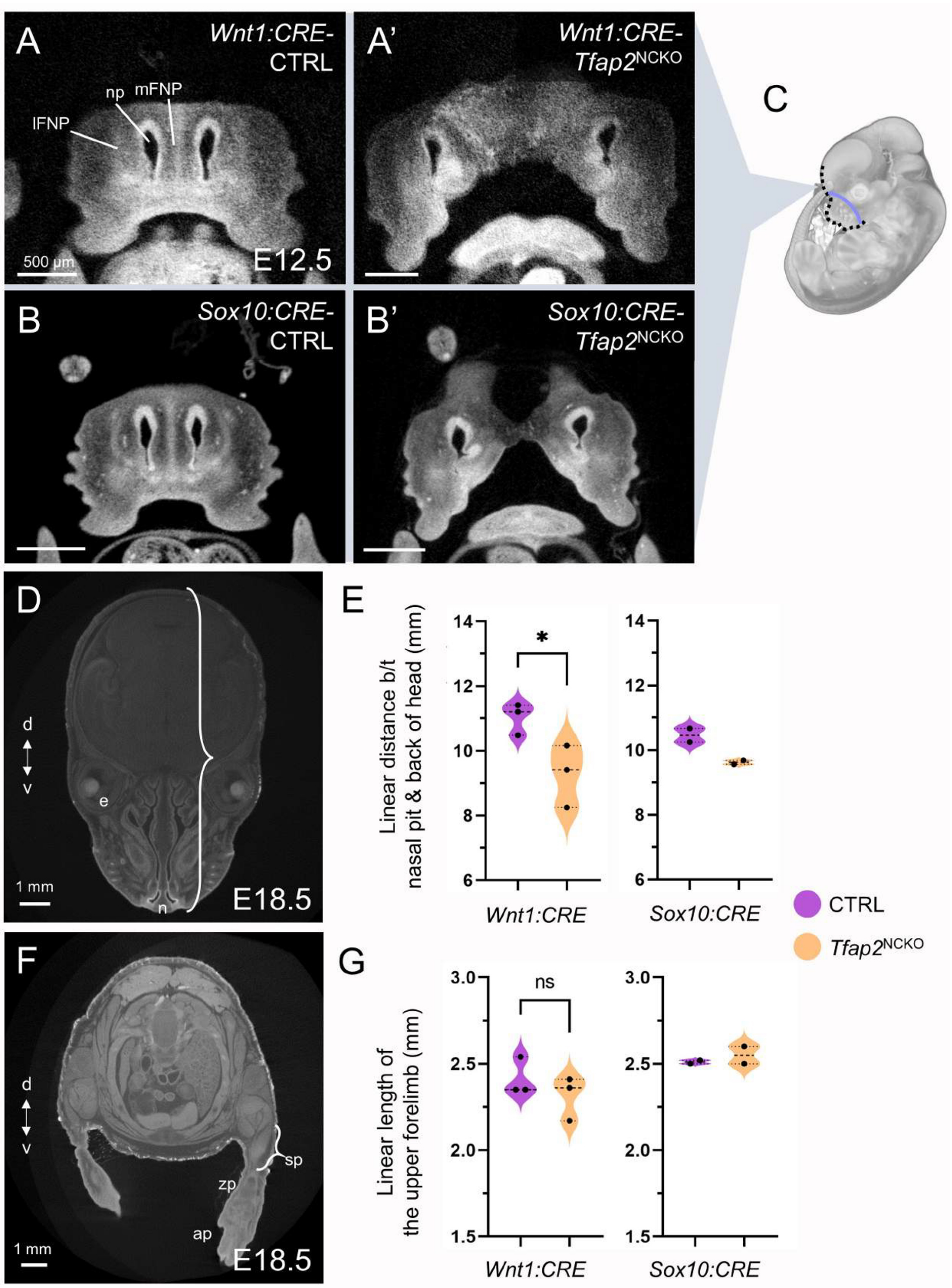

Fig. S6. Additional micro-computed tomography analyses of midfacial defects between *Wnt1:CRE*- and *Sox10:CRE-Tfap2*$^{\mathbf{NCKO}}$ mutants. (A, B, A', B') Micro-computed tomography (µCT) sections (frontal plane) of E12.5 (A, A') *Wnt1:CRE* and (B, B') *Sox10:CRE* frontonasal prominence, with indicated genotype. N = 3 per genotype. **(C)** Representative three-dimensional recostruction of an embryo, with the purple line indicating the plane viewed in panels A, B, A', and B'. **(D)** Representative µCT section horizontal plane through an E18.5 embryo head used to quantify midfacial growth, from the anterior tip of the nares to the

Development • Supplementary information

back of the head. **(E)** Violin plots of quantified head lengths between controls (violet) and mutants (orange) littermates from *Wnt1:CRE* (left, N = 3 per genotype) and *Sox10:CRE* (right, N = 2 per genotype) breeding schemes. **(F)** Representative μCT section horizontal plane through an E18.5 forelimb. The length of the stylopod (sp), from tendon to tendon, was used as an internal control. Note that both forelimbs could not be fully captured in the same plane. **(G)** Violin plots of quantified sp lengths between controls (violet) and mutants (orange) littermates from *Wnt1:CRE* (left, N = 3 per genotype) and *Sox10:CRE* (right, N = 2 per genotype) breeding schemes. Student's t-test, $^{*}p < 0.05$. Additional abbreviations: ap, autopod; d, dorsal; FNP, frontonasal prominence; lFNP, lateral domains of the FNP; mFNP, medial domains of the FNP; n, nares; np, nasal pit; v, ventral; zp, zeugopod.

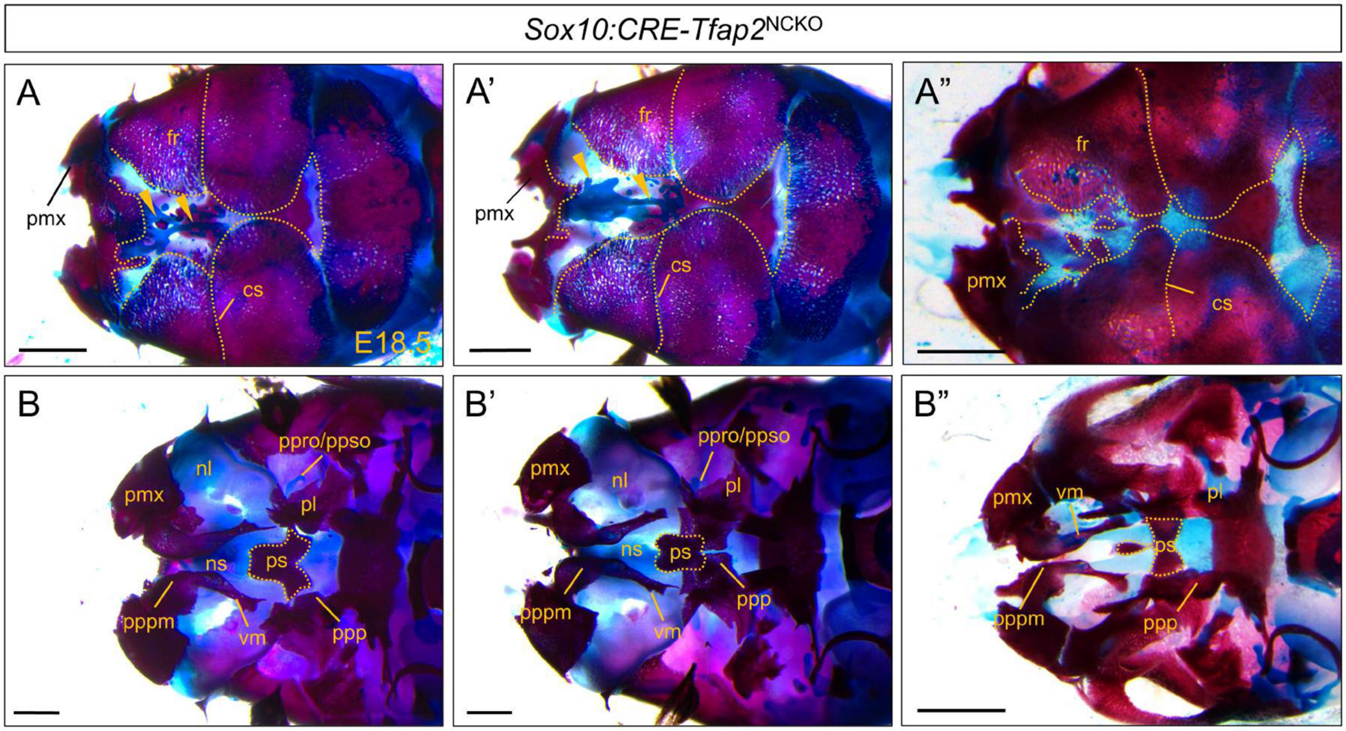

Fig. S7. Defects in the midfacial skeleton of *Sox10:CRE-Tfap2*^{NCKO} mutants. (**A-A", B-B"**) Three individual E18.5 *Sox10:CRE-Tfap2*^{NCKO} skeletal preparations in top-down (A) or bottom-up (B) view. Anterior is to the left. Yellow dashed lines in the top-down view outline the peripheral edges of the calvarial bones while those in the bottom-up view outline the presphenoid bone. Yellow arrowheads point to the cartilaginous ectopias stemming from the nasal septum. Note, in contrast to the first two embryos, the maxillary bone has been removed from the third. Further, the third exhibits isolated Alizarin red stained tissue (i.e., bony 'islands', individually outlined) on the calvaria instead of the cartilaginous ectopias. Scale bar = 1 mm. Abbreviations: cs, coronal suture; fr, frontal bone; nl, nasal/ethmoid labyrinth; ns, nasal septum; pl, palatine; pmx, premaxilla; ppp, palatal process of the palatine; pppm, palatal process fo the premaxilla; ppro, pila preoptica; ppso, pila postoptica; ps, presphenoid; vm, vomer bone.

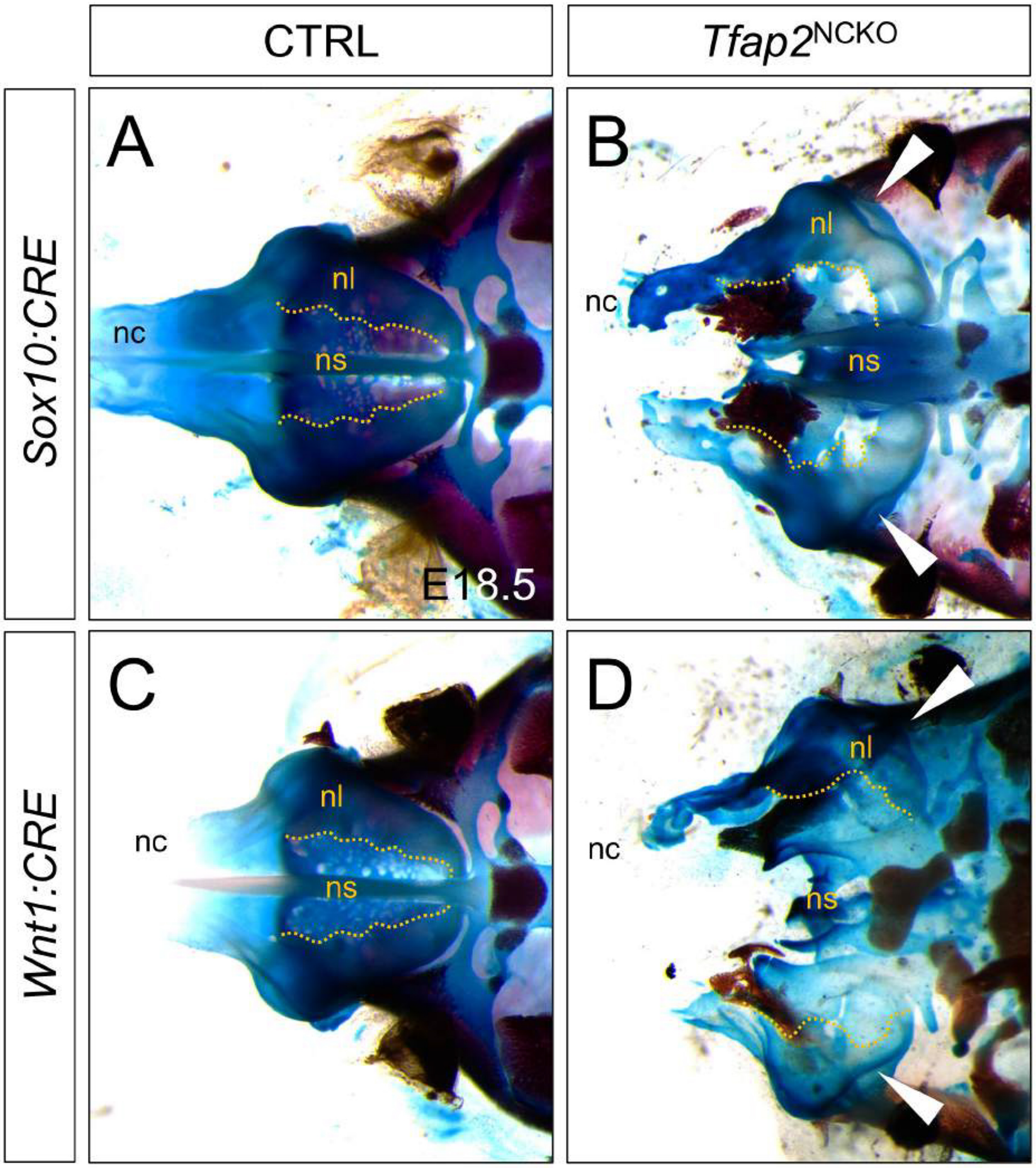

Fig. S8. ***Sox10:CRE-Tfap2*^{NCKO} and *Wnt1:CRE-Tfap2*^{NCKO} nasal labyrinths exhibit inflated morphology. (A-D)** Magnified, bottom-up views of the nasal capsules (nc) and nasal/ethmoid labyrinth (nl) of *Sox10:CRE* (A, B) and *Wnt1:CRE* (C, D) animals, with indicated genotypes. Dashed lines trace the nasal labyrinth periphery, while white arrowheads point to inflated regions in the posterior end. Additional abbreviations: ns, nasal septum.

Development • Supplementary information

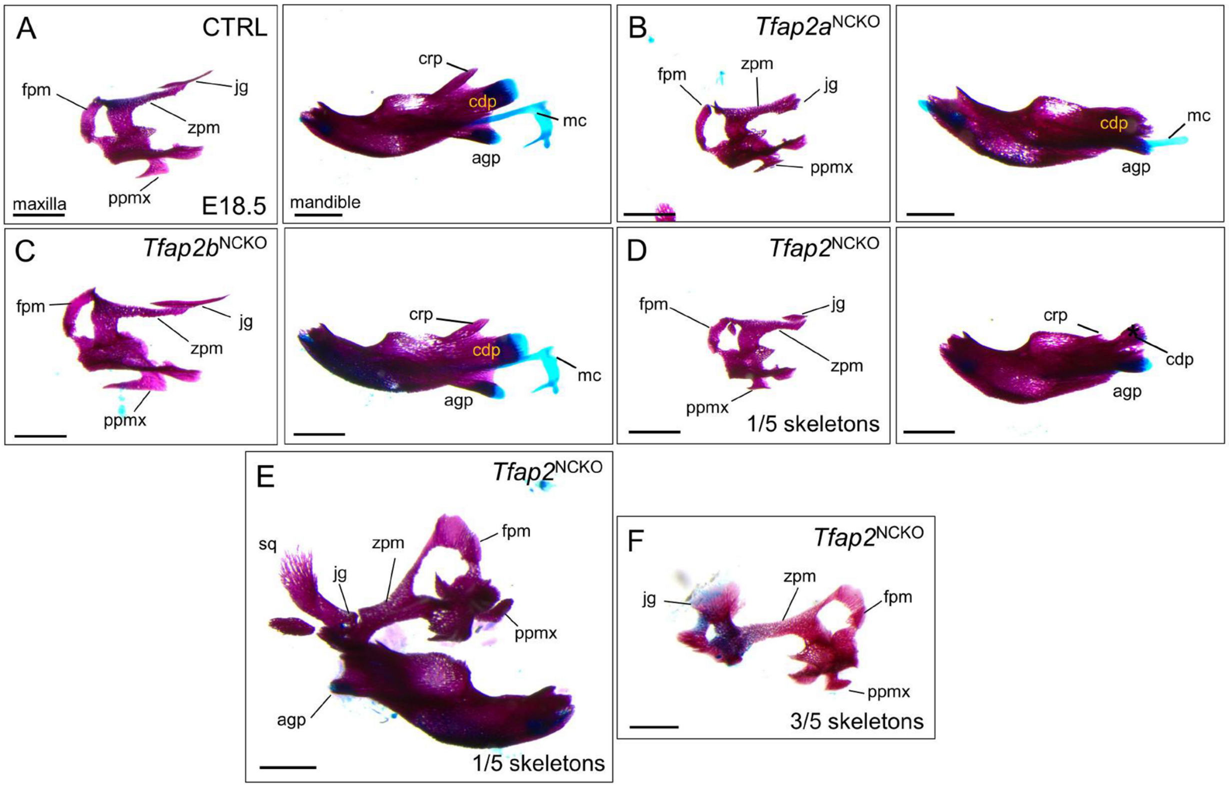

Fig. S9. Jaw phenotypes in *Sox10:CRE-Tfap2*^{NCKO} mutants partially recapitulate those observed in *Wnt1:CRE-Tfap2*^{NCKO} and *Tfap2*$^{\Delta/NCKO}$ mutants. (A-F) E18.5 maxillary and mandibular bones in isolation, with indicated genotypes. In panels A through D, the left-side elements are presented; panels E and F show the right-side elements. In panel B, circled is the missing jugal bone and thicked zygomatic process of the maxilla. In panel E, the sygnanthia is circled. In panel F, fusion between the jugal and thickened zygomatic process of the maxilla is circled. N = 5 per genotype. Scale bar = 1 mm. Abbreviations: agp, angular process; cdp, condylar process; crp, coronoid process; fpm, frontal process of the maxilla; jg, jugal bone; mc, Meckel's cartilage; ppmx, palatal process of the maxilla; sq, squamosal bone; zpm, zygomatic process of the maxilla.

Development • Supplementary information

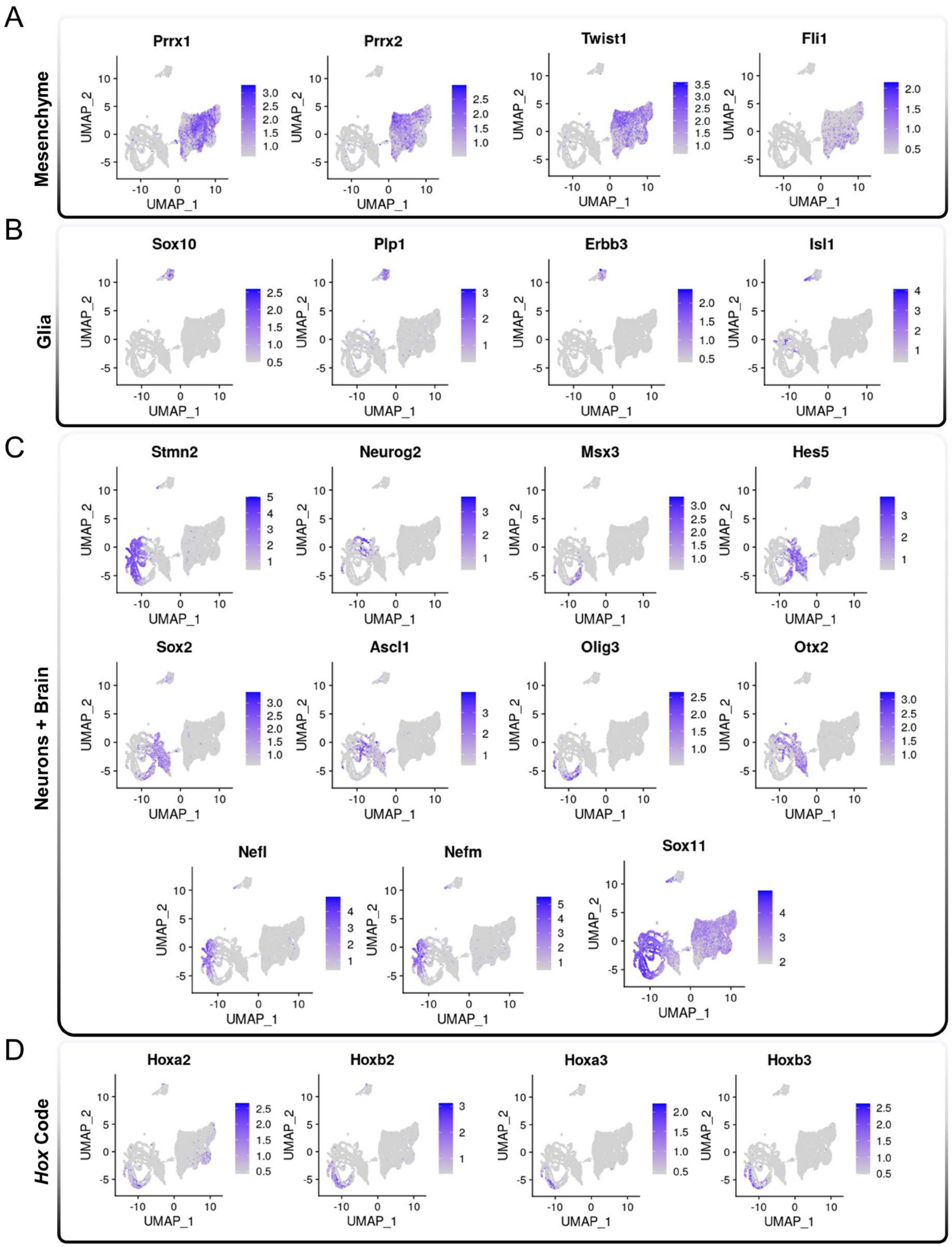

Fig. S10. Gene expression-based scRNA-seq annotation of the major CNCC lineages. (**A-D**) Gene expression of select genes mapped onto the Uniform Manifold Approximation and Projection (UMAP) plot in Fig. 4C. These genes were selected based on published scRNA-seq datasets (Soldatov et al., 2019). Displayed are gene signatures for (A) mesenchyme, (B) glia, (C) neurons and parts of the brain based on *Wnt1:CRE* labelling, and (D) *Hox* genes.

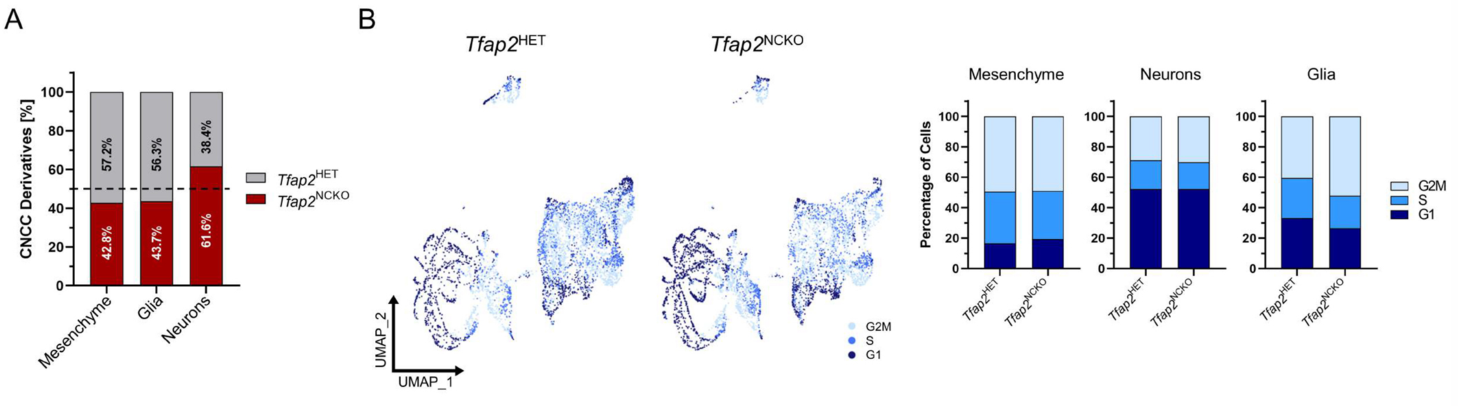

Fig. S11. Cellular profiling of the three major CNCC lineages in the developing head. (**A**) *Tfap2*^{HET} and *Tfap2*^{NCKO} representation in the integrated dataset, divided by the three major groupings and expressed as a percentage. The dashed line marks the fifty-percent threshold. (**B**) Gene expression-based cell cycle scoring for indiivdual *Tfap2*^{HET} and *Tfap2*^{NCKO} conditions. Cell cycle scores are mapped on the Uniform Manifold Approximation and Projection (UMAP) plots of individual conditions (left) and visualized as percentages per CNCC lineage.

A

Terms from Mesenchyme (Downregulated Genes)

MGI Mammalian Phenotype

Index	Name	P-value	Adjusted p-value	Odds Ratio	Combined score
1	small supraoccipital bone MP:0004444	0.000007995	0.0008806	123.17	1445.65
2	abnormal mandibular angle morphology MP:0004596	4.491e-7	0.0001034	94.61	1382.89
3	abnormal pterygoid process morphology MP:0004452	6.704e-7	0.0001034	82.78	1176.80
4	abnormal tongue muscle morphology MP:0004136	6.704e-7	0.0001034	82.78	1176.80
5	abnormal nasal capsule morphology MP:0004726	4.924e-8	0.00003796	69.55	1170.32
6	decreased tympanic ring size MP:0006020	0.000001343	0.0001725	66.22	895.35
7	abnormal alisphenoid bone morphology MP:0003235	0.00003701	0.002594	61.57	628.32
8	palatal shelf hypoplasia MP:0009883	0.00003701	0.002594	61.57	628.32
9	short endolymphatic duct MP:0008065	0.0005627	0.01735	81.44	609.41
10	abnormal mandibular condyloid process morphology MP:0004595	0.0005627	0.01735	81.44	609.41

InterPro Domains

Index	Name	P-value	Adjusted p-value	Odds Ratio	Combined score
1	Homeobox domain	0.00002387	0.001814	6.46	68.73
2	OAR domain	0.0001228	0.003760	37.88	341.13
3	Cytosolic fatty-acid binding	0.0001484	0.003760	35.17	310.08
4	LSM domain, eukaryotic/archaea-type	0.0002455	0.004664	28.96	240.75
5	Histone H2A, C-terminal domain	0.0003762	0.005719	24.61	194.10
6	Lactate/malate dehydrogenase, C-terminal	0.001042	0.01127	54.29	372.79
7	Lactate/malate dehydrogenase, N-terminal	0.001042	0.01127	54.29	372.79
8	Paired domain	0.001334	0.01127	46.53	308.01
9	Lipocalin/cytosolic fatty-acid binding domain	0.001210	0.01127	15.87	106.62
10	Ribosomal protein L7Ae/L30e/S12e/Gadd45	0.002022	0.01537	36.19	224.50

GO Molecular Function

Index	Name	P-value	Adjusted p-value	Odds Ratio	Combined score
1	RNA binding (GO:0003723)	7.524e-17	1.392e-14	6.45	239.56
2	double-stranded DNA binding (GO:0003690)	0.000002751	0.0002544	4.49	57.48
3	telomerase RNA binding (GO:0070034)	0.000009443	0.0005823	36.77	425.49
4	snoRNA binding (GO:0030515)	0.00003405	0.001203	25.45	261.81
5	mRNA binding (GO:0003729)	0.00003901	0.001203	6.05	61.37
6	sequence-specific double-stranded DNA binding (GO:1990837)	0.00003522	0.001203	3.79	38.83
7	sequence-specific DNA binding (GO:0043565)	0.0001256	0.003320	3.52	31.64
8	damaged DNA binding (GO:0003684)	0.0001577	0.003647	16.53	144.72
9	U4 snRNA binding (GO:0030621)	0.0005627	0.01157	81.44	609.41
10	DNA N-glycosylase activity (GO:0019104)	0.001042	0.01928	54.29	372.79

GO Cellular Component

Index	Name	P-value	Adjusted p-value	Odds Ratio	Combined score
1	nucleus (GO:0005634)	3.438e-7	0.00004160	2.60	38.73
2	box C/D RNP complex (GO:0031428)	0.000004589	0.0001388	164.24	2018.80
3	nucleolus (GO:0005730)	0.000002857	0.0001388	4.25	54.27
4	nuclear lumen (GO:0031981)	0.000003550	0.0001388	4.18	52.44
5	U7 snRNP (GO:0005683)	0.000007995	0.0001935	123.17	1445.65
6	U4 snRNP (GO:0005687)	0.00001901	0.0003287	82.11	892.54
7	microtubule (GO:0005874)	0.00001807	0.0003287	7.81	85.28
8	cytosolic large ribosomal subunit (GO:0022625)	0.00002293	0.0003468	16.66	177.98
9	spliceosomal tri-snRNP complex (GO:0097526)	0.00004425	0.0004118	23.63	236.89
10	U4/U6 x U5 tri-snRNP complex (GO:0046540)	0.00004425	0.0004118	23.63	236.89

B

Terms from Mesenchyme (Upregulated Genes)

GO Biological Process

Index	Name	P-value	Adjusted p-value	Odds Ratio	Combined score
1	extracellular matrix organization (GO:0030198)	3.552e-19	6.965e-16	9.84	418.11
2	supramolecular fiber organization (GO:0097435)	3.309e-17	3.245e-14	8.25	313.13
3	collagen fibril organization (GO:0030199)	2.986e-14	1.952e-11	17.74	552.46
4	extracellular structure organization (GO:0043062)	8.101e-13	3.478e-10	8.84	246.24
5	external encapsulating structure organization (GO:0045229)	8.867e-13	3.478e-10	8.80	244.18
6	pyruvate metabolic process (GO:0006090)	1.954e-9	6.387e-7	17.58	352.49
7	glycolytic process (GO:0006096)	2.408e-9	6.746e-7	29.93	593.94
8	canonical glycolysis (GO:0061621)	1.615e-8	0.000003519	32.23	578.23
9	glucose catabolic process to pyruvate (GO:0061718)	1.615e-8	0.000003519	32.23	578.23
10	glycolytic process through glucose-6-phosphate (GO:0061620)	2.218e-8	0.000004350	30.44	536.41

InterPro Domains

Index	Name	P-value	Adjusted p-value	Odds Ratio	Combined score
1	Follistatin/Osteonectin EGF domain	0.00002106	0.003812	115.66	1245.43
2	Follistatin-like, N-terminal	0.0001702	0.01540	38.54	334.51
3	Fibrillar collagen, C-terminal	0.0003280	0.01899	28.91	231.90
4	Fork head domain	0.0004196	0.01899	8.81	68.53
5	EMI domain	0.001061	0.03841	17.78	121.79
6	Immunoglobulin subtype 2	0.001273	0.03841	3.38	22.51
7	Immunoglobulin I-set	0.001833	0.04738	4.29	27.04
8	SPARC/Testican, calcium-binding domain	0.003361	0.05359	30.72	174.95
9	Tubulin/FtsZ, 2-layer sandwich domain	0.002402	0.05359	12.84	77.44
10	Coagulation factor 5/8 C-terminal domain	0.003139	0.05359	11.56	66.60

GO Molecular Function

Index	Name	P-value	Adjusted p-value	Odds Ratio	Combined score
1	platelet-derived growth factor binding (GO:0048407)	1.519e-7	0.00003691	64.75	1016.53
2	cadherin binding (GO:0045296)	0.0003108	0.03777	3.32	26.84
3	ER retention sequence binding (GO:0046923)	0.001628	0.04526	51.20	328.73
4	procollagen-proline 4-dioxygenase activity (GO:0004656)	0.001628	0.04526	51.20	328.73
5	MHC class II protein binding (GO:0042289)	0.002421	0.04526	38.40	231.29
6	dihydropyrimidinase activity (GO:0004157)	0.002421	0.04526	38.40	231.29
7	fructose binding (GO:0070061)	0.002421	0.04526	38.40	231.29
8	DNA binding, bending (GO:0008301)	0.001061	0.04526	17.78	121.79
9	cell-cell adhesion mediator activity (GO:0098632)	0.002090	0.04526	8.13	50.19
10	GTP binding (GO:0005525)	0.0008249	0.04526	3.91	27.78

GO Cellular Component

Index	Name	P-value	Adjusted p-value	Odds Ratio	Combined score
1	endoplasmic reticulum lumen (GO:0005788)	8.135e-18	1.643e-15	9.60	377.64
2	collagen-containing extracellular matrix (GO:0062023)	3.970e-17	4.010e-15	7.86	296.67
3	intracellular organelle lumen (GO:0070013)	6.315e-14	4.252e-12	4.55	138.20
4	focal adhesion (GO:0005925)	7.638e-12	3.857e-10	5.99	153.34
5	cell-substrate junction (GO:0030055)	1.141e-11	4.611e-10	5.87	148.01
6	COPI-coated vesicle (GO:0030137)	9.328e-7	0.00003140	103.21	1433.02
7	polymeric cytoskeletal fiber (GO:0099513)	0.000006737	0.0001944	4.60	54.83
8	actin filament (GO:0005884)	0.00004049	0.001022	8.41	85.05
9	supramolecular fiber (GO:0099512)	0.00009138	0.002051	20.63	191.86
10	neuron projection (GO:0043005)	0.0001243	0.002283	2.83	25.46

Fig. S12. Enrichment analyses of down-regulated genes in the scRNA-seq mesenchyme group. Terms from the Enrichr pipeline (Kuleshov et al., 2016), based on (**A**) down-regulated or (**B**) up-regulated genes identified through the mesenchyme pseudobulk analysis (Table S2, Tab 9).

Development • Supplementary information

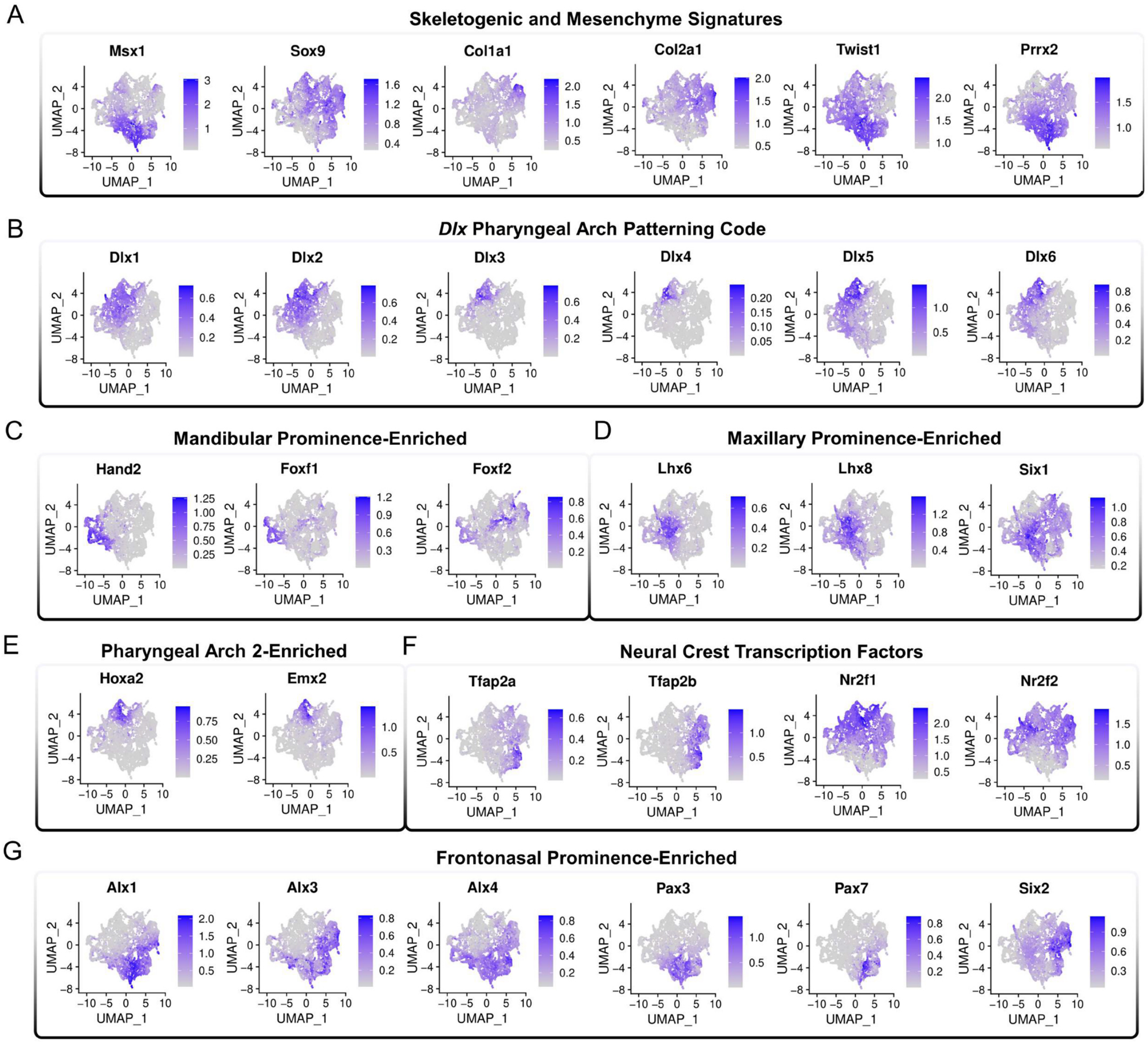

Fig. S13. Gene expression-based scRNA-seq annotation of the major CNCC mesenchymal populations. (**A-G**) MAGIC-based (van Dijk et al., 2018) gene expression of select genes mapped onto the Uniform Manifold Approximation and Projection (UMAP) plot in Fig. 4D. These genes were selected based on published transcriptomic datasets (Gu et al., 2022; Hooper et al., 2020), epigenome datasets (Minoux et al., 2017), and published *in situ* data. Displayed are (A) skeletogenic/mesenchyme markers, (B) *Dlx* paralogs patterning the pharyngeal arches, additional (C) mandibular and (D) maxillary markers, (E) pharyngeal arch 2 markers, (F) neural crest transcription factor genes, and (G) frontonasal markers.

Development • Supplementary information

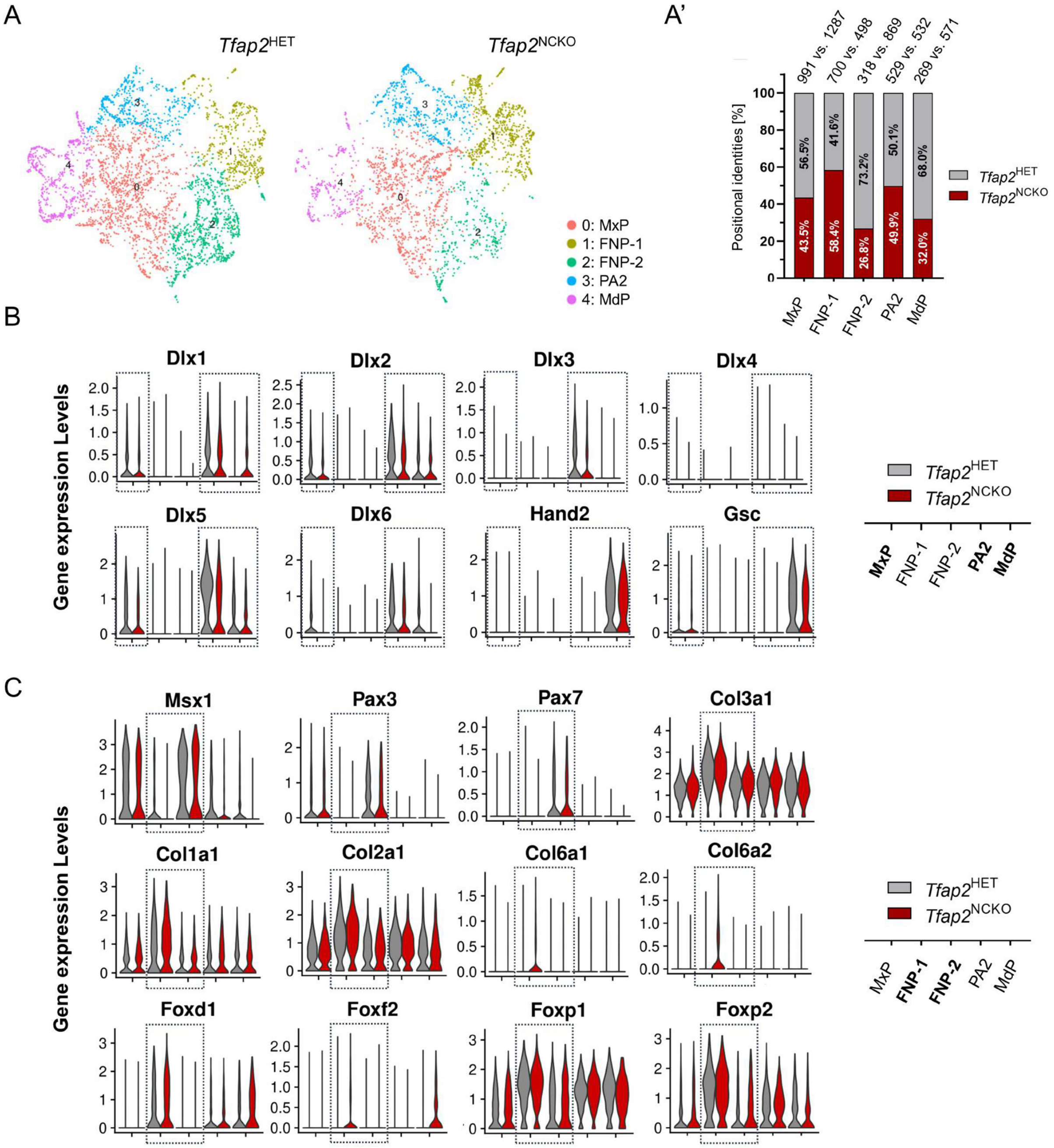

Fig. S14. Cellular and gene expression profiling in the CNCC-derived mesenchyme. (**A**) Cellular distributions of *Tfap2*^{HET} and *Tfap2*^{NCKO} conditions as Uniform Manifold Approximation and Projection (UMAP) plots (A) or as a percentage in each cluster (A'). (**B**) Violin expression plots of pharyngeal arch (PA)-enriched genes previously identified (Van Otterloo et al., 2018). Boxed are clusters for the maxillary prominence (MxP), mandibular prominence (MdP), and PA2. (**C**) Violin expression plots for genes dysregulated in the midface. Boxed are the frontonasal prominence (FNP) clusters.

Development • Supplementary information

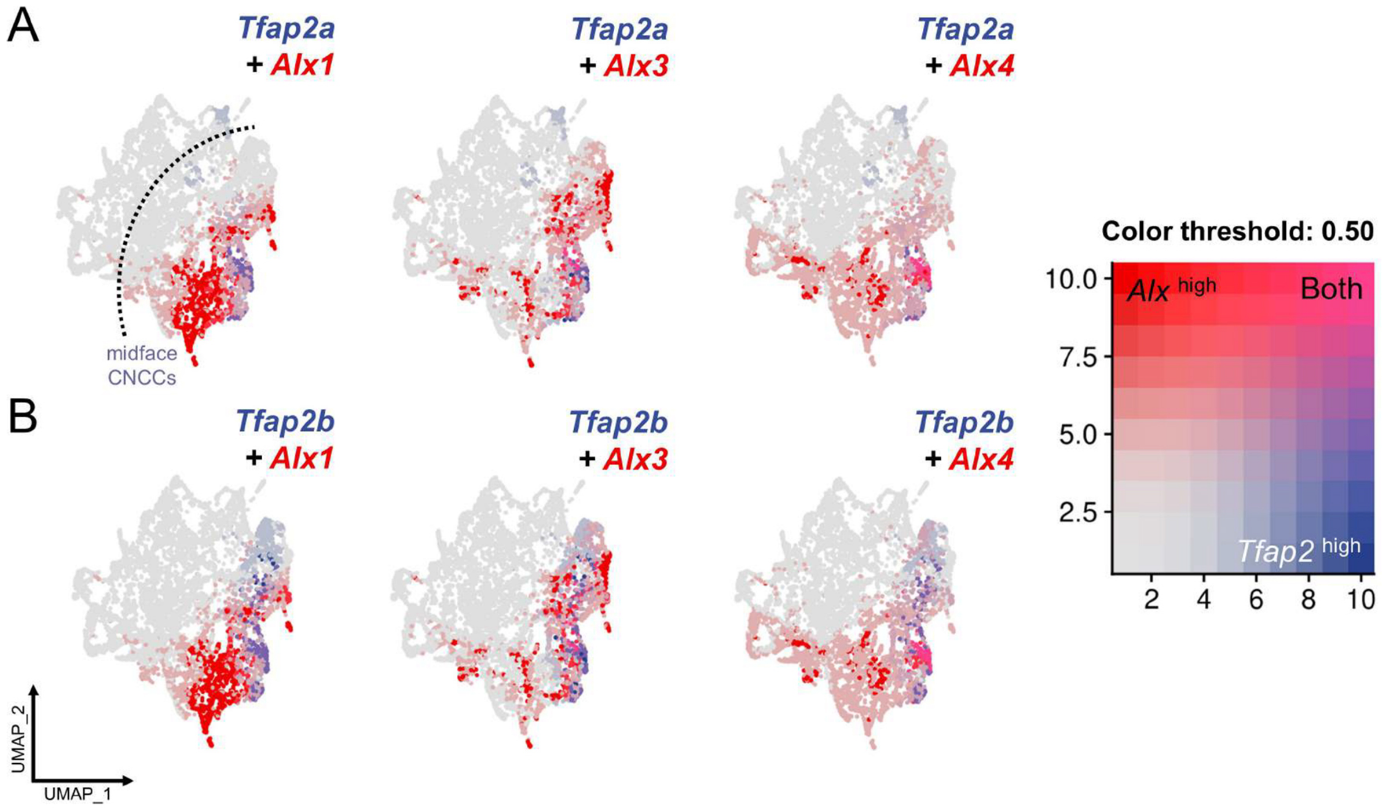

Fig. S15. *Tfap2* and *Alx* paralog transcripts are enriched in the midfacial CNCCs. (**A, B**) Gene expression of (A) *Tfap2a* and (B) *Tfap2b* (both in blue) overlaid with individual *Alx1/3/4* paralogs (red) onto the Uniform Manifold Approximation and Projection (UMAP) plot**.** Color saturation correlates to gene expression levels, while degree of co-expression is read out as a blending of the two colors.

Development • Supplementary information

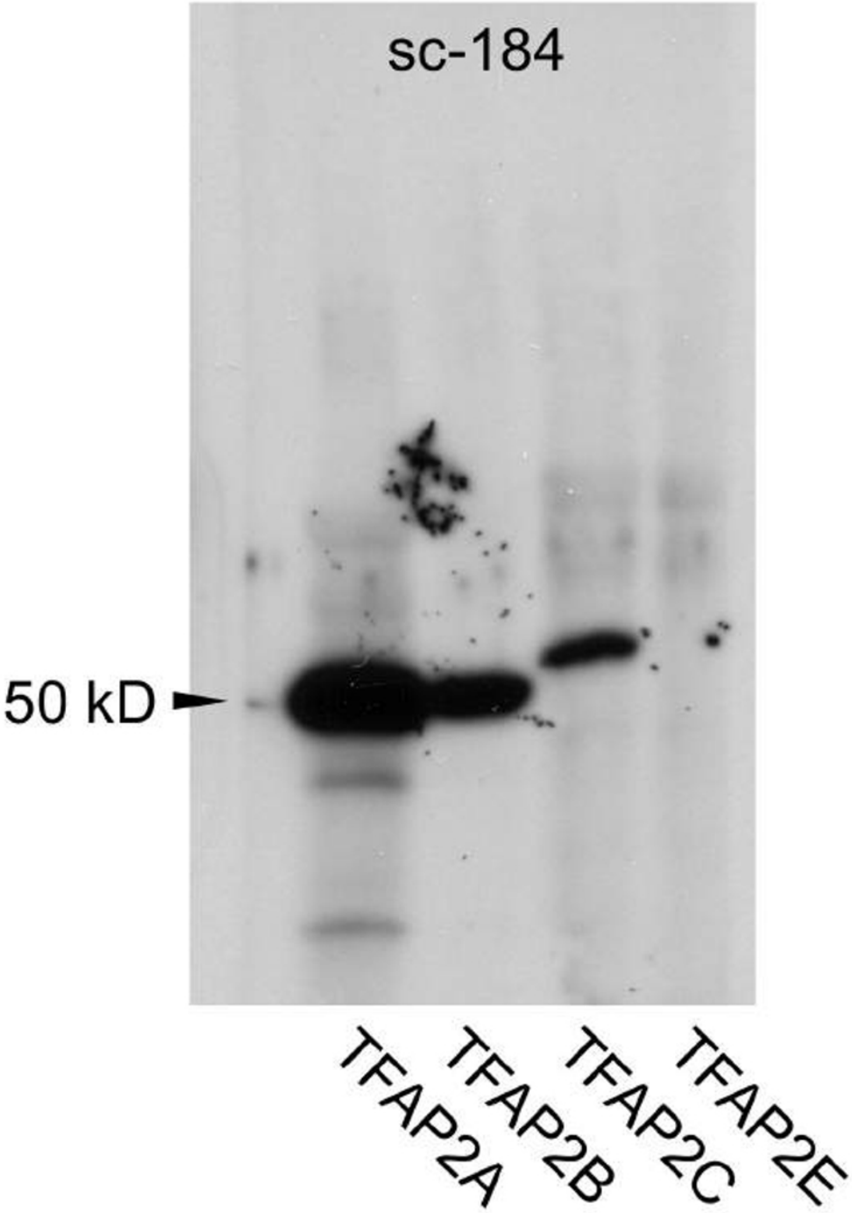

Fig. S16. The sc-184 antibody recognizes TFAP2A, TFAP2B, and TFAP2C protein. A western blot of *in vitro* transcribed and translated TFAP2A, TFAP2B, TFAP2C, or TFAP2E protein, with the first three detected by the sc-184 antibody.

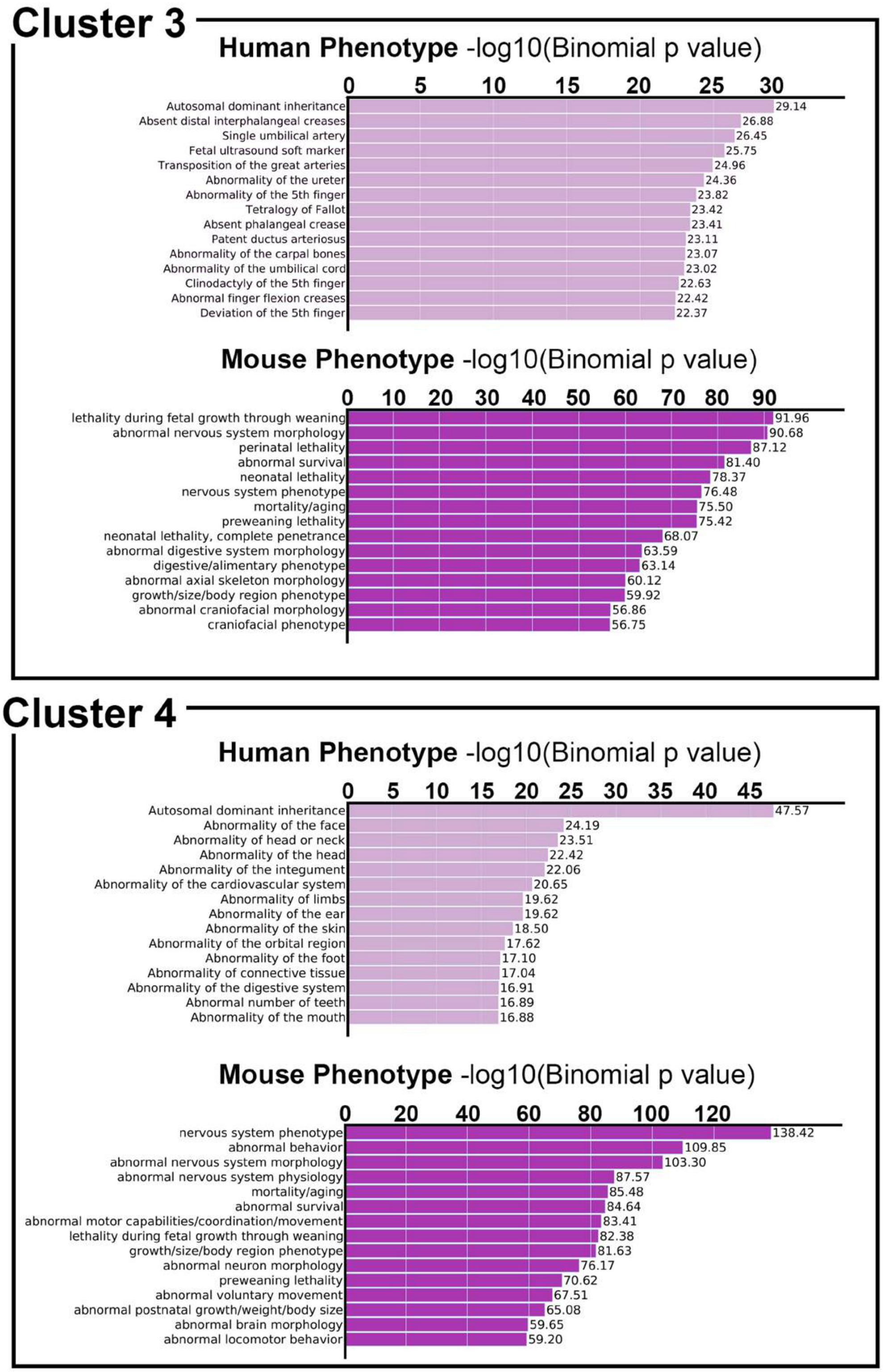

Fig. S17. Additional GREAT pathway analysis on TFAP2 genomic binding. Terms enriched in cluster 3 and 4 peaks that contained positive TFAP2 ChIP-seq signal. Note, peaks in these clusters are significantly enriched for skin (cluster 4), neuronal (clusters 3, 4), and cardiac (cluster 3) terms.

Development • Supplementary information

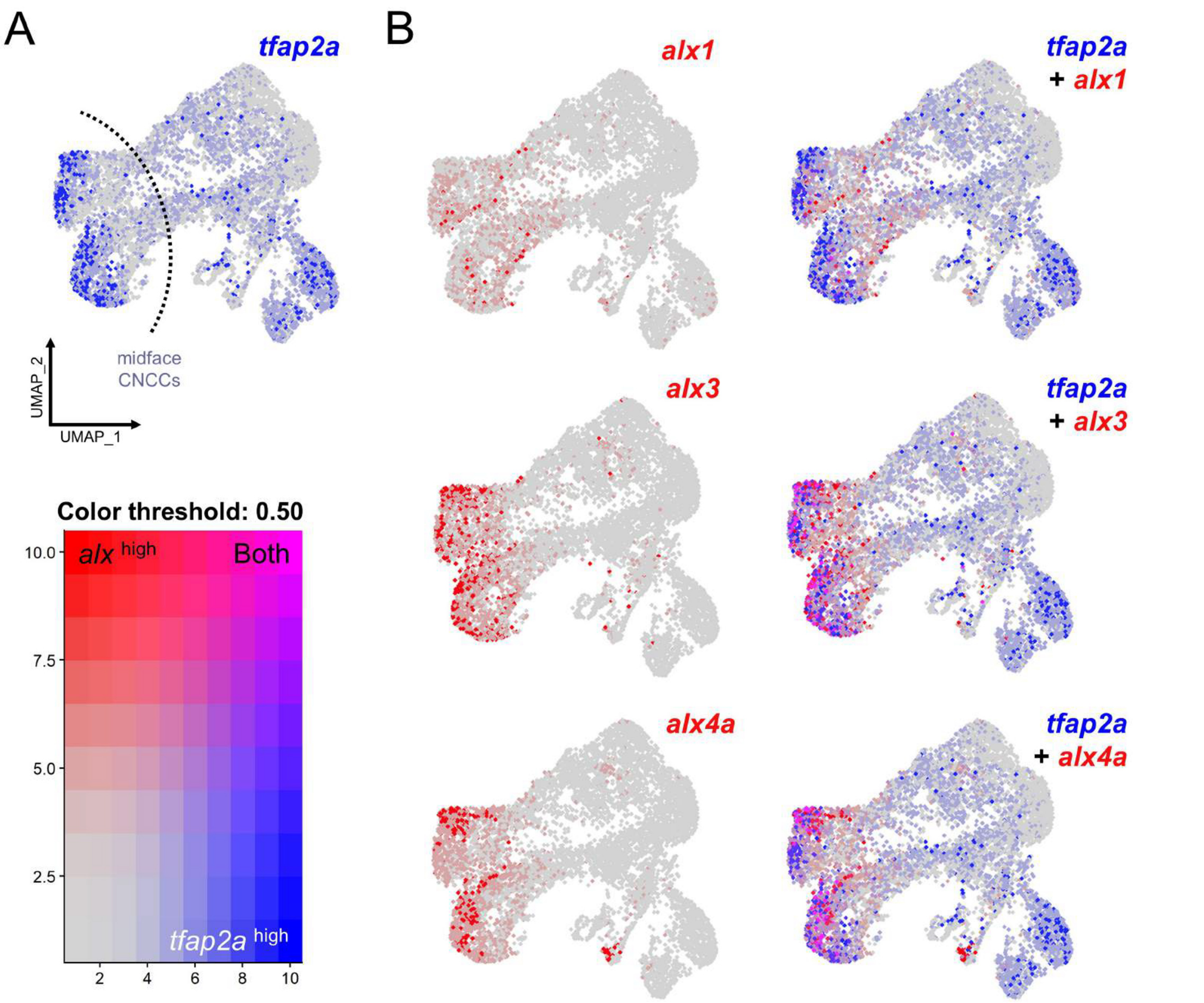

Fig. S18. *tfap2a* and *alx* transcripts are enriched in the zebrafish frontonasal CNCCs. Gene expression of *tfap2a* and *alx1*, *alx3*, or *alx4a* overlaid together on a Uniform Manifold Approximation and Projection (UMAP) plot of a published single-cell RNA-seq dataset generated from sorted zebrafish cranial neural crest cells (Stenzel et al., 2022)**.** Color saturation correlates to gene expression levels, while degree of co-expression is read out as a blending of the two colors.

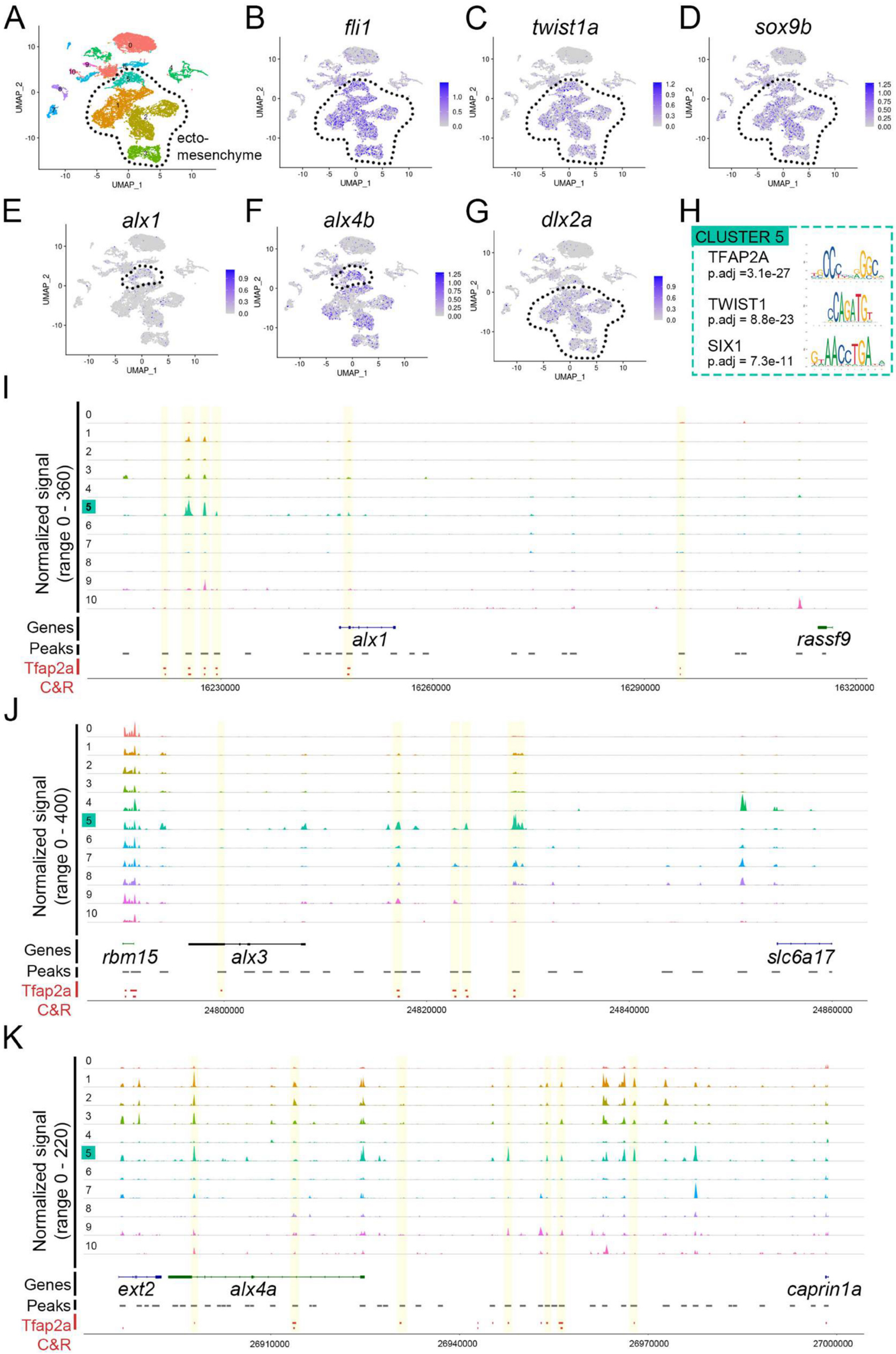

Fig. S19. Analysis of Tfap2a binding at *alx* loci in zebrafish. (A-H) Reanalysis of single nuclei ATAC-seq conducted on CNCCs zebrafish embryos at 1.5 days post-fertilization (Fabian et al., 2022). A)

Development • Supplementary information

Table S1.

Available for download at
https://journals.biologists.com/dev/article-lookup/doi/10.1242/dev.202095#supplementary-data

Table S2.

Available for download at
https://journals.biologists.com/dev/article-lookup/doi/10.1242/dev.202095#supplementary-data

Table S3.

Available for download at
https://journals.biologists.com/dev/article-lookup/doi/10.1242/dev.202095#supplementary-data

Table S4. Zebrafish epistasis ana lyses with p-values

		tfap2a-/-			*p-values*		
		***alx3+/+* (n=11)**	***alx3+/-* (n=11)**	***alx3-/-* (n=11)**	**WT vs H**	**WT vs M***	**H vs M^**
tfap2a-/- associated phenotypes	ethmoid plate midline division	9%	27%	91%*^	0.5864	0.0003	0.0075
	ethmoid plate ectopic cartilage rods	27%	64%	91%*	0.1984	0.0075	0.3108
	cartilaginous eye ectopias	36%	73%	100%*	0.1984	0.0039	0.2143
***tfap2a-/-; alx3* new phenotype**	**ectopic parasphenoid**	0%	18%	64%*	0.4762	0.0039	0.0805

		alx3-/-			*p-values*		
		***tfap2a+/+* (n=14)**	***tfap2a+/-* (n=19)**	***tfap2a-/-* (n=15)**	**WT vs H**	**WT vs M***	**H vs M^**
alx3-/- associated phenotypes	ethmoid plate cell morphology defect	100%	95%	ND	1	ND	ND
	ethmoid plate ectopic midline cartilage	57%	79%	ND	0.2569	ND	ND
	parasphenoid bone loss	90%	74%	ND	0.6328	ND	ND

		alx3+/-			*p-values*		
		***tfap2a+/+* (n=13)**	***tfap2a+/-* (n=30)**	***tfap2a-/-* (n=11)**	**WT vs H**	**WT vs M***	**H vs M^**
alx3+/- associated phenotypes	ethmoid plate cell morphology defect	15%	20%	82%*^	0.6992	0.0031	0.0005
	ethmoid plate ectopic midline cartilage	38%	20%	82%*^	0.2619	0.0472	0.0005
	parasphenoid bone loss	0%	7%	64%*^	1	0.001	0.0004

Table S5.

Available for download at
https://journals.biologists.com/dev/article-lookup/doi/10.1242/dev.202095#supplementary-data

Table S6.

Available for download at
https://journals.biologists.com/dev/article-lookup/doi/10.1242/dev.202095#supplementary-data

Development • Supplementary information

REFERENCES

Armit, C., Richardson, L., Venkataraman, S., Graham, L., Burton, N., Hill, B., Yang, Y. and Baldock, R. A. (2017). eMouseAtlas: An atlas-based resource for understanding mammalian embryogenesis. *Dev Biol* **423**, 1-11.

Fabian, P., Tseng, K. C., Thiruppathy, M., Arata, C., Chen, H. J., Smeeton, J., Nelson, N. and Crump, J. G. (2022). Lifelong single-cell profiling of cranial neural crest diversification in zebrafish. *Nat Commun* **13**, 13.

Gu, R., Zhang, S., Saha, S. K., Ji, Y., Reynolds, K., McMahon, M., Sun, B., Islam, M., Trainor, P. A., Chen, Y., et al. (2022). Single-cell transcriptomic signatures and gene regulatory networks modulated by Wls in mammalian midline facial formation and clefts. *Development* **149**.

Hooper, J. E., Jones, K. L., Smith, F. J., Williams, T. and Li, H. (2020). An Alternative Splicing Program for Mouse Craniofacial Development. *Front Physiol* **11**, 1099.

Kuleshov, M. V., Jones, M. R., Rouillard, A. D., Fernandez, N. F., Duan, Q., Wang, Z., Koplev, S., Jenkins, S. L., Jagodnik, K. M., Lachmann, A., et al. (2016). Enrichr: a comprehensive gene set enrichment analysis web server 2016 update. *Nucleic Acids Res* **44**, W90-97.

McLean, C. Y., Bristor, D., Hiller, M., Clarke, S. L., Schaar, B. T., Lowe, C. B., Wenger, A. M. and Bejerano, G. (2010). GREAT improves functional interpretation of cis-regulatory regions. *Nat Biotechnol* **28**, 495-501.

Minoux, M., Holwerda, S., Vitobello, A., Kitazawa, T., Kohler, H., Stadler, M. B. and Rijli, F. M. (2017). Gene bivalency at Polycomb domains regulates cranial neural crest positional identity. *Science* **355**.

Soldatov, R., Kaucka, M., Kastriti, M. E., Petersen, J., Chontorotzea, T., Englmaier, L., Akkuratova, N., Yang, Y., Haring, M., Dyachuk, V., et al. (2019). Spatiotemporal structure of cell fate decisions in murine neural crest. *Science* **364**.

Stenzel, A., Mumme-Monheit, A., Sucharov, J., Walker, M., Mitchell, J. M., Appel, B. and Nichols, J. T. (2022). Distinct and redundant roles for zebrafish her genes during mineralization and craniofacial patterning. *Front Endocrinol (Lausanne)* **13**, 1033843.

van Dijk, D., Sharma, R., Nainys, J., Yim, K., Kathail, P., Carr, A. J., Burdziak, C., Moon, K. R., Chaffer, C. L., Pattabiraman, D., et al. (2018). Recovering Gene Interactions from Single-Cell Data Using Data Diffusion. *Cell* **174**, 716-729 e727.

Van Otterloo, E., Li, H., Jones, K. L. and Williams, T. (2018). AP-2α and AP-2β cooperatively orchestrate homeobox gene expression during branchial arch patterning. *Development* **145**.
