# Supplementary material for: TFAP2 paralogs regulate midfacial development in part through a conserved ALX genetic pathway
Source: Development. 2024 Jan 2;151(1):dev202095. doi: 10.1242/dev.202095 (PMC10820886; doi:10.1242/dev.202095)
Supplement: Supplementary file 1 [file develop-151-202095_review_history.pdf]

TFAP2 paralogs regulate midfacial development in part through a conserved *ALX* genetic pathway.

Timothy T. Nguyen, Jennyfer M. Mitchell, Michaela D. Kiel, Colin P. Kenny, Hong Li, Kenneth L. Jones, Robert A. Cornell, Trevor J. Williams, James T. Nichols and Eric Van Otterloo
DOI: 10.1242/dev.202095

Editor: Benoit Bruneau

Review timeline

Original submission:	16 June 2023
Editorial decision:	25 July 2023
First revision received:	6 November 2023
Accepted:	27 November 2023

Original submission

First decision letter

MS ID#: DEVELOP/2023/202095

MS TITLE: TFAP2 paralogs regulate midfacial development in part through a conserved *ALX* genetic pathway.

AUTHORS: Timothy T. Nguyen, Jennyfer M. Mitchell, Michaela D. Kiel, Kenneth L. Jones, Trevor J. Williams, James T. Nichols, and Eric Van Otterloo

I have now received all the referees reports on the above manuscript, and have reached a decision. The referees' comments are appended below, or you can access them online: please go to BenchPress and click on the 'Manuscripts with Decisions' queue in the Author Area.

The overall evaluation is positive and we would like to publish a revised manuscript in Development, provided that the referees' comments can be satisfactorily addressed. Please attend to all of the reviewers' comments in your revised manuscript and detail them in your point-by-point response. If you do not agree with any of their criticisms or suggestions explain clearly why this is so. If it would be helpful, you are welcome to contact us to discuss your revision in greater detail. Please send us a point-by-point response indicating your plans for addressing the referees' comments, and we will look over this and provide further guidance.

Reviewer 1

Advance summary and potential significance to field

The manuscript by Nguyen et al investigates TFAP- gene regulatory elements members in the cranial neural crest during early midfacial morphogenesis. Using mouse conditional knockouts, they demonstrate phenotypic outcomes resulting from TFAP2 inactivation at different stages of cNC development. They then provide RNA-seq and ChIP-seq evidence that TFAP2 directly regulates downstream Alx family genes.
Finally, they present findings from zebrafish studies to argue that the Tfap2-Alx GRN is conserved across vertebrates. The presented findings are largely novel and supported by high quality data,

© 2023. Published by The Company of Biologists under the terms of the Creative Commons Attribution License (https://creativecommons.org/licenses/by/4.0/).

and would be of significant interest to the developmental biology community. The manuscript could be improved by addressing the comments below.

Comments for the author

Major comments

1. The authors integrate findings from mouse and zebrafish studies to support the contention that TFAP2 regulation of midfacial development through ALX transcription factor is conserved among vertebrates. The data presented support this contention in the broadest sense. However, the weight of evidence is far stronger for the mouse than for zebrafish, and some species specific differences suggested by the data are not directly addressed. For example mouse data demonstrate TFAP occupancy of Alx regulatory elements and reduced Alx (including Alx3) expression in cNCCs from TFAP cKO embryos. In zebrafish tfap mutants, the alx3 expression domain appears dramatically expanded and quantitative expression data are not provided. While phenotypic analysis of compound zebrafish mutants suggests some genetic interaction, no evidence is provided that tfap directly regulates alx expression in this model. This is important because the abstract states that "TFAP2 directly and positively regulates Alx gene expression" and that their overall evidence "implies conservation of this regulatory axis across vertebrates". The authors should discuss the differences observed between mouse and zebrafish experiments provide relevant context, offer plausible explanations. The conclusory statement regarding conservation across species in the abstract should be modified accordingly.

2. The authors describe their investigation and results within the framework of "midface fusion". This is a bit misleading given the approach of neural crest-specific genetic deletion. Moreover, the phenotype of widely spaced medial nasal processes suggests that midface fusion is not even plausible in the resultant mutants. The authors may then consider whether "midfacial patterning/morphogenesis" is a more appropriate development context in which to frame this study.

Minor points

1. The authors use Wnt1cre and Sox10cre to target Tfap2 gene deletion and generate data demonstrating that TFAP activity, particularly in post-migratory cNCCs, is critical for midface patterning. This point would be strengthened if the temporal expression of Wnt1 versus Sox10 in cNCC biology was more clearly described. If possible, the authors should also provide whole mount images of the midfacial phenotypes in Sox10cre cKOs that are directly comparable to those of Wnt1Cre cKOs presented in Figure 1.

2. Cellular mechanisms are not addressed in this study. While it is understandable to focus on GRNs, this leaves a major knowledge gap for how these molecular changes are translated into the documented phenotypes.

3. The introduction describes frequency of "typical" orofacial clefts of the secondary palate and lateral clefts of the upper lip. This should be more clearly differentiated from the median clefts relevant to the study, which typically present as features of specific syndromes, and much less common.

<u>Reviewer 2</u>

Advance summary and potential significance to field

This work explores the targets downstream of the TFAP2 family members during mid-craniofacial development. These factors are known to be important for craniofacial development. This work further determines the consequences of losing TFAP2 paralogs, both at the phenotypic level and at the deep genomic level. The key insight to the gene regulatory network is a direct role in regulating ALX factors which are also crucial for craniofacial development.

Comments for the author

© 2023. Published by The Company of Biologists under the terms of the Creative Commons Attribution License (https://creativecommons.org/licenses/by/4.0/).

In this manuscript, Nguyen and colleagues present convincing evidence that TFAP2 factors are redundant and have effects on DNA accessibility. Furthermore, ALX factors are demonstrated to be downstream of TFAP2 factors and genetic data is presented to support this finding. Overall, the data are of high quality and support the conclusions drawn. My comments are almost entirely at ideas to improve the flow of the information presented and I am not sure any additional experiments will markedly improve the story being told in this paper.

It is very clear to this reviewer how much this follows on from the 2018 van otterloo et al., paper. However, I would suggest that this be written as more of an independent work. The integration of the 2018 work is quite confusing as currently presented. Would it perhaps be easier and more clear to definitively summarize what was shown in that paper and move to the new experiments with the new models?

The non-standard genetic nomenclature as presented is very tough for the reader to internalize to then move on to really appreciate the data. Might actually be clearer to write out the actual alleles - i.e., Cre; Tfap2a null/flox;Tfap2b wt/ flox vs SCM-A or Cre;Tfap2a flox/flox;Tfap2b flox/flox vs NCKO. While these abbreviations have likely become second hand to the authors, they are a barrier to full understanding for the first time reader (in the opinion of this reviewer). If the null/conditional results are under-emphasized in a revised version of this manuscript, this may not be as necessary.

Why would the clefting phenotypes lead to fewer vibrissae – death of tissue? Can/ should this be examined with a couple of choice in situs?

The data shown (e.g., S4A) look like there might be reduced cranial neural crest cells in the Tfap2 NCKO. Can the authors comment or provide data better supporting the hypothesis.

I wonder if the Tfap2 NCKO phenotypes are even more striking if shown in whole mount embryos in Fig. 2 vs buried in supplement?

Fig S1 might not be necessary given the 2018 Dev paper.

I think several abbreviations are used for terms which are referenced infrequently enough, I would suggest removing these abbreviations totally: FND, BOFS, FNP, MxP MdP.

Line 200-205. I feel a stronger/clearer set of statements stating the conclusions about the Wnt1 vs Sox10 Cre experiments would be helpful

The bulk seq experiment is perhaps a bit of an outlier. It has the feel of “old data” that is looking for a home and was added to this story. This is especially true given the pseudobulk approach taken with the scRNA Seq data. Maybe if the initial bulk data is mentioned more as a backup of the RNASeq?? It does not look like the main conclusions would be different, or any less strongly supported.

Line 296/ Fig. 5. Should the background filter for the Hooper data set be higher than FPKM>0?

What do you make of the fact the alx3 expression in the fish ap2 mutants seems clearly enriched? I appreciate the reduction around the periphery of the nasal placodes but the overall conclusion would seem to be different than that seen in the mouse. The genetic interaction data in the fish is clear but the molecular results shown do not necessarily make a “neat” and complementary set of experiments.

Line 64 – what is meant by “encoded”?
BA1 – used in the literature along with PA1 often enough, I wonder if it is worth writing out branchial arch Line 67 – illuminate or elucidate?
Line 142 – which comprise of – word choice?
Line 149 – sectional?

Methods are very well written and comprehensive. There are a number of references to van Otterloo 2018 but at least some of those in turn cite van Otterloo 2016 (e.g. skeletal preps). I

© 2023. Published by The Company of Biologists under the terms of the Creative Commons Attribution License (https://creativecommons.org/licenses/by/4.0/).

would recommend avoiding the serial citations and either writing a brief summary of the methods, or citing a more original source.

Reviewer 3

Advance summary and potential significance to field

In their study, Nguyen and colleagues show that midfacial clefting in Tfap2 cranial neural crest (CNCC-specific) knockouts in mice is likely due primarily to post-migratory requirements for Tfap2a/b. They perform bulk and single cell RNA-sequencing (scRNA-seq) analysis of frontonasal (FNP) and maxillary (MxP) processes as well as sorted CNCCs to identify genes dysregulated in response to loss of Tfap2, finding Alx genes along with other genes known to function in midfacial development. Their ChIP-seq data indicate that Tfap2s directly regulate Alx transcription. They also show genetic interactions between tfap2 and alx3 mutations in zebrafish, suggesting that the GRN involving these factors is conserved.

Comments for the author

Mutations in both human TFAP2 and ALX genes cause midfacial birth defects and the most significant result in the paper is the discovery of a midfacial GRN driven by Tfap2 that regulates these and other homebox-containing genes distinct from previously described regulation in the mandibular arch. However, much of the study appears to recapitulate their previous work using Tfap2 null/floxed animals (Van Otterloo et al., 2018). Comparisons between Wnt1:CRE and Sox10:CRE driven conditional knockouts are used to test if Tfap2 function is required early in CNCC migration or later in post-migratory CNCCs with regard to the midfacial clefting phenotype. However, in their previous study, the authors showed that mutants had “no distinct changes in CNCC migration into the facial complex” and concluded that “Tfap2a and Tfap2b are largely dispensable for early NCC development” (Van Otterloo et al., 2018). Thus the justification and rationale for performing the Wnt1:CRE vs. Sox10:CRE experiments is weak. In addition, the data provided and referenced by the authors are insufficient to rule out functions for Tfap2 in early midfacial CNCCs, particularly in light of the more severe clefting observed in Tfap2 null/floxed animals for which they provide no explanation.
Major comments
1) The authors argue that their data suggest “that the midface cleft does not arise from impaired TFAP2 activity during migration” (line 164). However, the comparatively more severe phenotype of Wnt1:CRE conditional knockouts as compared to Sox10:CRE suggests that there are earlier TFAP2 contributions to midfacial clefting, or alternatively that these two CRE lines are not expressed in the same midfacial subpopulations of CNCCs at later stages (e.g. E12.5) (Debbache et al., 2018), a possibility that they acknowledge (lines 201-203) but which undermines several of their interpretations. While CNCCs clearly populate the arches in single and double conditional KOs, as shown in their previous study, the pattern of LacZ staining in these embryos compared to controls at E10.5 would seem to suggest some CNCC migration defects. Without a more direct assay of cell migration, the authors cannot definitively conclude that there is no migratory contribution to the phenotype they observe. It may be more appropriate to say that the postmigratory loss of TFAP2 activity has an outsized effect on midfacial clefting or that midfacial clefting is primarily due to postmigratory loss of TFAP2 activity.
2) The authors state that “further co-expression analyses indicate that Tfap2a-positive cells also express Alx3 (Fig. S16), suggesting that TFAP2A regulation of ALX gene expression is a conserved transcriptional axis in vertebrates.” However the observation that two genes are co-expressed (here, based on RNA-seq data) does not necessarily suggest that one regulates the other. Further, they attempt to test this relationship by performing in situ hybridization for Alx3 in Tfap2a mutants. However contrary to their interpretation these appear to show increased and expanded domains of Alx3 signal. Their HCR in situ methodology should allow the authors to quantify some of their expression data to support their claims.
3) There is a general lack of quantification and rigor in many experiments. For example, referring to phenotypes as “Mildly shortened,” with no measurements (lines 117-118). Other examples include a lack of n #s for described phenotypes merely stating that one was “overall, more common” (line 191).
4) In Fig S4 the genotypes shown in the fluorescent antibody staining panels are unclear. Each panel or set of panels should be appropriately labeled.

© 2023. Published by The Company of Biologists under the terms of the Creative Commons Attribution License (https://creativecommons.org/licenses/by/4.0/).

Additionally, the figure legend describes “absent TFAP2B protein expression in the nasal epithelium, in contrast to the top panel” however, there clearly appears to be staining, though with reduced intensity, in the nasal epithelium, similar to the reduced intensity in mesenchyme in the top half of the image panel. Could the authors explain this discrepancy?
5) In several places the authors refer to “data not shown” and it is unclear to which data they refer (lines 287, 320-321, 327). Development also strongly discourages this usage.

6) For the zebrafish data the interpretation “that the transcriptional circuit identified in post-migratory CNCCs is conserved between mice and zebrafish” should be toned-down (lines 353-354).
7) Also for the zebrafish data and new tfap2a mutant partially penetrant phenotypes, the authors describe “ectopic cartilage rods projecting dorsally from the ethmoid plate (Fig. 6J)” and while “ethmoid plate anterior dorsal ectopic cartilage” is listed in the table, no example is shown or indicated in the figure panels.
General minor comments:
The ChIPseq experiment utilizes an antibody that recognizes TFAP2A/B/C proteins. A comparison of ChIPseq results between Tfap2a/b null would reveal more specifically which sites are occupied by TFAP2A or B versus TFAP2C, though this is minor.
Supplementary figure panel labeling is confusing and inconsistent. Each figure panel should be labelled with a letter for easy and specific referral in the text and legend, similar to the main figures.
With so many similar abbreviations throughout, the authors should be particularly careful in verifying that they are consistent across the main text, figure legends, and supplementary materials (see specific minor comments below). For example, Figure S7 legend/Line 944: Abbreviation “ppmx” is used for “palatal process of the maxilla” but in Figure S6 legend, “ppmx” is used for “palatal process of the premaxilla” and in Figure 3 “pppm” is used for “palatal process of the premaxilla.”
Specific minor comments:
- Fig 4 legend “the dysregulated Alx3/4 staining in the MdP that was previously characterized (Van Otterloo et al., 2018).” I think this statement should be in the main text and not figure legend (lines 846-847:.
-Line 143 “…a mandibular prominences (MdP)” to “…and mandibular prominences (MdP)”.
-Figure S6: There is some type of graphical error in the “2” of “Sox10:CRE-Tfap2” at top of figure.
-Figure S6 legend: “ps” is not listed among abbreviations.
-Figure S6: In panels B and B’, abbreviation “ppmx” is used but in panel B” and Figure 3, “pppm” is used.
-Line 935: Both “ppro” & “ppso” abbreviations are erroneously listed for “pila postoptica” while “pila preoptica” is omitted. Also, “fo” should be corrected to “of.”
-Line 941: Extra “the” in “fusion between the the jugal…”
-Line 186: It is difficult to appreciate the “less compacted nasal labyrinths” the authors describe in Figure 3 or S6. They appear similar to control.
-Line 188: Specifically, what does the “both” in “both Wnt1:CRE animals” refer to?
-Figure S6: The authors’ use of “jg?” suggests they are unsure of the identity of the labelled element though the manuscript text and Figure S6 legend unquestionably refer to the jg bone. Are or are not the authors confident in this?
If not, then the text should better convey this uncertainty.
-Figure S8 and legend. SS and CS abbreviations are capitalized in the figure but lowercase in the legend.
-Figure 6: The figure would be more appealing if panels A-D” were in the same box rather than separated A, B and C, D. Similarly, panels F-I should not be separated from their companion panels F’-I’.
-The authors should provide more information in their methods regarding HCR: e.g. the NCBI reference sequence provided to Molecular Instruments for probe design how many probes were in the gene set, what amplifier (B1, B2, B3…).

© 2023. Published by The Company of Biologists under the terms of the Creative Commons Attribution License (https://creativecommons.org/licenses/by/4.0/).

First revision

Author response to reviewers' comments

Reviewer 1

- *The manuscript by Nguyen et al investigates TFAP- gene regulatory elements members in the cranial neural crest during early midfacial morphogenesis. Using mouse conditional knockouts, they demonstrate phenotypic outcomes resulting from TFAP2 inactivation at different stages of cNC development. They then provide RNA-seq and ChIP-seq evidence that TFAP2 directly regulates downstream Alx family genes. Finally, they present findings from zebrafish studies to argue that the Tfap2-Alx GRN is conserved across vertebrates. The presented findings are largely novel and supported by high quality data, and would be of significant interest to the developmental biology community. The manuscript could be improved by addressing the comments below.*

  We appreciate Reviewer 1's praise and their thoughtful comments below.

- *The authors integrate findings from mouse and zebrafish studies to support the contention that TFAP2 regulation of midfacial development through ALX transcription factor is conserved among vertebrates. The data presented support this contention in the broadest sense. However, the weight of evidence is far stronger for the mouse than for zebrafish, and some species specific differences suggested by the data are not directly addressed. For example, mouse data demonstrate TFAP occupancy of Alx regulatory elements and reduced Alx (including Alx3) expression in cNCCs from TFAP cKO embryos. In zebrafish tfap mutants, the alx3 expression domain appears dramatically expanded and quantitative expression data are not provided. While phenotypic analysis of compound zebrafish mutants suggests some genetic interaction, no evidence is provided that tfap directly regulates alx expression in this model. This is important because the abstract states that "TFAP2 directly and positively regulates Alx gene expression" and that their overall evidence "implies conservation of this regulatory axis across vertebrates". The authors should discuss the differences observed between mouse and zebrafish experiments, provide relevant context, offer plausible explanations. The conclusory statement regarding conservation across species in the abstract should be modified accordingly.*

  We thank the reviewer for pointing out these critical details. We agree that species-specific differences in the spatiotemporal execution of this network likely exist and may underlie differences in craniofacial morphology between species. However, to assess the basic conservation of this network more thoroughly, we have provided additional data suggesting that 1) as in mouse, zebrafish Tfap2a binds *alx* loci, and 2) although nuanced in how domains respond, expression of *alx3* is decreased within medial domains of *tfap2a* zebrafish mutants.

  Regarding the first point, we have carried out anti-Tfap2a CUT&RUN experiments on 24-hour post fertilization wild-type embryos. While conducted on whole embryo samples, we have coupled this data with a single-nuclei ATAC-seq dataset of cranial neural crest cells, previously published by Fabian et al., 2022 (PMID: 35013168). By combining both datasets, we show that Tfap2a-bound regions surrounding *alx* loci (*alx1*, *alx3*, and *alx4a*) overlap with elements that are specific to the presumptive 'upper face' neural crest cells. These data have now been included as a supplement (new Fig. S19).

  Regarding the latter, we have carried out a more thorough quantification of *alx3* expression domains in *tfap2a* zebrafish mutants (new Fig. 6C). Consistent with a role for Tfap2a in regulating *alx3* expression, we find a statistically significant decrease of *alx3* in medial domains of *tfap2a* mutants, relative to controls. Like our single-cell RNA-seq analysis in the mouse, not all expression domains are equally impacted, suggesting additional levels of gene regulation beyond TFAP2 in both species. Further, given the level of evolutionary 'distance' between zebrafish and mouse and the manipulation of one paralog (versus two paralogs in the mouse), it is not surprising to find differences between models. Nevertheless, both

© 2023. Published by The Company of Biologists under the terms of the Creative Commons Attribution License (https://creativecommons.org/licenses/by/4.0/).

direct binding and localized changes in expression patterns are consistent with a level of regulatory logic shared between species. We have modified the zebrafish sections to reflect these findings.

- *The authors describe their investigation and results within the framework of "midface fusion". This is a bit misleading given the approach of neural crest-specific genetic deletion. Moreover, the phenotype of widely spaced medial nasal processes suggests that midface fusion is not even plausible in the resultant mutants. The authors may then consider whether "midfacial patterning/morphogenesis" is a more appropriate development context in which to frame this study.*

We thank the reviewer for this careful distinction and have made changes throughout the Results section to reflect our investigations on midface "development", "morphogenesis", or "patterning"— rather than "fusion".

- *The authors use Wnt1cre and Sox10cre to target Tfap2 gene deletion and generate data demonstrating that TFAP activity, particularly in post-migratory cNCCs, is critical for midface patterning. This point would be strengthened if the temporal expression of Wnt1 versus Sox10 in cNCC biology was more clearly described.*

Thank you for this suggestion. Unfortunately, because we no longer have *Sox10:CRE* animals in our colonies, this precludes us from generating new samples to directly compare the *CRE* drivers in greater detail. That said, *Tfap2a/b* deletion in neural crest was rationalized based on already published reports that directly compared *Wnt1:CRE* and *Sox10:CRE* expression patterns via a ß-gal reporter allele:
Consistent with the initial characterization made by Danielian et al., 1998 (PMID: 9843687), Stottmann et al. 2004 (PMID: 15073157, their Fig 1) and Hari et al., 2012 (PMID: 22573620, their Fig 2A) showed *Wnt1:CRE* animals labelled pre-migratory neural crest at E8.0. By contrast, no apparent staining of neural crest was detected in E8.0 *Sox10:CRE* animals (PMID: 22573620, their Fig 2B).
Further refining the stage by which *Sox10:CRE* begins labeling neural crest, Jacques-Fricke et al., 2012 (PMID: 23094090) detected ß-gal staining at 13 somites (~E9.0) (their Fig 3E). Based on the staining pattern, most cranial neural crest cells have reached the face (both frontonasal mass and first arch) while the remaining cells follow close behind. Thus, we reasoned that *Sox10:CRE* is a late- migratory neural crest driver.
We have modified the Methods when describing the alleles, summarizing the rationale:
"Based on published lineage tracing studies (Hari et al., 2012; Jacques-Fricke et al., 2012; Stottmann et al., 2004), we note that *Wnt1:CRE* recombines in pre-migratory CNCCs (~E8.0), whereas *Sox10:CRE* recombines when most CNCCs have finished migrating into the embryonic face (~E9.0)."

- *If possible, the authors should also provide whole mount images of the midfacial phenotypes in Sox10cre cKOs that are directly comparable to those of Wnt1Cre cKOs presented in Figure 1.*

We initially placed images of E18.5 *Sox10:CRE* embryos of various *Tfap2* allelic combinations in supplement. We unfortunately did not image lateral views of these E18.5 embryos by brightfield and no longer have the animals to generate more embryos. To more cleanly accommodate this suggestion, we added μCT 3D reconstruction images of E18.5 control and mutants from both *CRE* lines to Fig. 2. The previous E12.5 microCT images viewed in a ventral plane were moved to supplement to accommodate this change (new Fig. S6).

- *Cellular mechanisms are not addressed in this study. While it is understandable to focus on GRNs, this leaves a major knowledge gap for how these molecular changes are translated into the documented phenotypes.*

We agree that cellular mechanisms underlying the midfacial phenotypes in these animals remain a critical knowledge gap. To assess this, we have conducted proliferation and death analysis via phospho-Histone H3 and TUNEL staining at E11.5, respectively. Herein, we found

© 2023. Published by The Company of Biologists under the terms of the Creative Commons Attribution License (https://creativecommons.org/licenses/by/4.0/).

statistically significant decreases in pHH3+ CNCCs in both medial and lateral domains of the FNP—albeit differences were subtle. Further, TUNEL staining did not detect any differences in cell death between mutant and control embryos. Collectively, these data suggest that post-migratory proliferation deficiencies are likely a contributing factor to midface defects in mutant embryos. We have stated such now in the manuscript.

"Compared to controls, *Tfap2*^{NCKO} mutants presented reduced cell proliferation in the E11.5 midfacial CNCCs as marked by phospho-Histone H3 immunofluorescence (Fig. S4E-G), while no cell death was observed by TUNEL staining (Fig. S4H, H', I, I'). In summary, lineage tracing revealed subtle changes in midfacial CNCC distribution in *Tfap2*^{NCKO} embryos relative to controls, possibly due to reduced rates of proliferation."

- *The introduction describes frequency of "typical" orofacial clefts of the secondary palate and lateral clefts of the upper lip. This should be more clearly differentiated from the median clefts relevant to the study, which typically present as features of specific syndromes, and much less common.*

Thank you for pointing this out. We have corrected this in the Introduction:

"Although much less prevalent, clefts can also occur at the midline of the medial- and upper-face region forming the forehead, nose, and cheeks (Tessier, 1976), generating a spectrum of midface pathologies that includes Frontonasal Dysplasia (MIMs: 136760, 613451, 613456) (Farlie et al., 2016; Vargel et al., 2021)."

Reviewer 2

- *In this manuscript, Nguyen and colleagues present convincing evidence that TFAP2 factors are redundant and have effects on DNA accessibility. Furthermore, ALX factors are demonstrated to be downstream of TFAP2 factors and genetic data is presented to support this finding. Overall, the data are of high quality and support the conclusions drawn. My comments are almost entirely at ideas to improve the flow of the information presented and I am not sure any additional experiments will markedly improve the story being told in this paper.*

We are grateful for Reviewer 2's supportive comments and suggestions for improving the manuscript.

- *It is very clear to this reviewer how much this follows on from the 2018 van otterloo et al., paper. However, I would suggest that this be written as more of an independent work. The integration of the 2018 work is quite confusing as currently presented. Would it perhaps be easier and more clear to definitively summarize what was shown in that paper and move to the new experiments with the new models?*

We agree that, as previously written, the 'compare and contrast' format of findings from the new model (i.e., flox/flox, previously called conditional/conditional) with the previous model (i.e., null/flox, previously called null/conditional) presented confusion. Therefore, numerous adjustments were made across the Results and Methods. We have now minimized comparisons mainly to the first section, including the analysis of pharyngeal arch 1 derivatives. These comparisons are now briefly summarized in the new Fig. 1E and skeletal phenotypes included in the new Fig. S3. Further, since gene expression profiles of the pharyngeal arches in the null/flox (Van Otterloo et al. 2018) and flox/flox models (new Fig. S14) are very similar (summarized in Table S1), we now minimize such statements in the transcriptomics sections and refer to the supplementary tables/figures should readers be interested in such details regarding their comparisons. Please find two examples of the adjusted text, from the Results section, below:

Section 1 (gross morphology): "Gross examination and skeletal analyses of *Tfap2*^{NCKO} embryos revealed that they recapitulate the major jaw abnormalities we documented in the null allele (Fig. 1D, asterisk, Fig. S3A-E, E') (Van Otterloo et al., 2018), with the exception

© 2023. Published by The Company of Biologists under the terms of the Creative Commons Attribution License (https://creativecommons.org/licenses/by/4.0/).

that a mandibular cleft was not observed. Thus, we surmised that while the midface cleft and jaw defects can be attributed solely to loss of *Tfap2a* and *Tfap2b* in the neural crest, overt mandibular clefting is caused by reduced gene dosage elsewhere—presumably the ectoderm (Fig. 1E). Note, that while the remainder of our study primarily focused on embryos acquired from the flox/flox scheme (Fig. S1B), embryos acquired from the scheme containing both null and floxed alleles are denoted with "Δ" in the superscript (Fig. S1C)."
Section 4 (transcriptomics): "Consistent with our previous findings and other past studies (Knight et al., 2003; Maconochie et al., 1999; Van Otterloo et al., 2018), mutant lower face CNCC populations showed reduced cell numbers and gene expression changes (Fig. S14A, A', B, Table S1)."

- *The non-standard genetic nomenclature as presented is very tough for the reader to internalize to then move on to really appreciate the data. Might actually be clearer to write out the actual alleles - i.e., Cre; Tfap2a null/flox;Tfap2b wt/ flox vs SCM-A or Cre;Tfap2a flox/flox;Tfap2b flox/flox vs NCKO. While these abbreviations have likely become second hand to the authors, they are a barrier to full understanding for the first time reader (in the opinion of this reviewer). If the null/conditional results are under-emphasized in a revised version of this manuscript, this may not be as necessary.*
Thank you for pointing out this deficiency that made the first submission less accessible to first-time readers and the broader *Development* readership. Towards addressing this, we have spelled out the full genotypes directly at the onset of their mentioning in the results, figure legends, and methods section—before subsequently shifting towards their shorthand nomenclature. We have also now moved details of alleles and breeding schemes (originally in Fig. 1) to a new Fig. S1.

- *Why would the clefting phenotypes lead to fewer vibrissae - death of tissue? Can/should this be examined with a couple of choice in situs?*

We apologize for the confusion, as the current text does not appropriately reflect our interpretations. In the initial submission, the wording was as follows for the *Tfap2a/b* double mutants:

"The misplaced vibrissae present by loss of a single paralog were not observed in *Tfap2*^{NCKO} embryos, but we surmised this was confounded by the facial cleft."

Indeed, compared to controls, single loss of either *Tfap2a* or *Tfap2b* in neural crest resulted in no cleft but led to misplaced vibrissae atop the nares. However, it was difficult to identify any misplaced vibrissae in *Tfap2a/b* double mutants due to the midfacial cleft by gross morphology. We have altered the text accordingly to prevent confusion:

"Note that because of the cleft, we could not identify by gross morphology whether *Tfap2*^{NCKO} embryos still harbored misplaced vibrissae atop the nares (Fig. 1D')."

- *The data shown (e.g., S4A) look like there might be reduced cranial neural crest cells in the Tfap2 NCKO. Can the authors comment or provide data better supporting the hypothesis.*

Thank you for raising this point. It is true that while CNCCs appear to have no trouble migrating into the face, their distribution, as well as the overall frontonasal prominence morphology, appears mildly altered. To test the possibilities that cell proliferation or death defects may contribute to this observation, we performed phospho-Histone H3 and TUNEL analysis, respectively, in E11.5 post- migratory CNCCs. Herein, we found that *Tfap2* mutants showed significant reduction (albeit mild) in pHH3 staining and no observation of cell death compared to control littermates (see new Fig. S4). An excerpt of our text edits describing these findings is directly below:

"Compared to controls, *Tfap2*^{NCKO} mutants presented reduced cell proliferation in the E11.5 midfacial CNCCs as marked by phospho-Histone H3 immunofluorescence (Fig. S4E-G), while no cell death was observed by TUNEL staining (Fig. S4H, H', I, I'). In summary, lineage tracing

© 2023. Published by The Company of Biologists under the terms of the Creative Commons Attribution License (https://creativecommons.org/licenses/by/4.0/).

revealed subtle changes in midfacial CNCC distribution in *Tfap2*^{NCKO} embryos relative to controls, possibly due to reduced rates of proliferation."

- *I wonder if the Tfap2 NCKO phenotypes are even more striking if shown in whole mount embryos in Fig. 2 vs buried in supplement?*

Reviewer 1 also commented on this. We unfortunately did not capture lateral views of the *Sox10:CRE* embryos by brightfield (previous Fig. S5). Further, we no longer have the *Sox10:CRE* allele to generate more embryos. To accommodate this suggestion for directly comparing gross morphology of *Wnt1:CRE* and *Sox10:CRE* mutants, we replaced the ventral sections of E12.5 µCT images previously shown in Fig. 2 with E18.5 µCT images of controls and mutants from both *CRE* models (see new Fig. 2E-H).

- *Fig S1 might not be necessary given the 2018 Dev paper.*

We have removed the old Fig. S1.

- *I think several abbreviations are used for terms which are referenced infrequently enough, I would suggest removing these abbreviations totally: FND, BOFS, FNP, MxP, MdP.*

We have removed FND and BOFS abbreviations from the manuscript text, although given their frequent use we have kept abbreviations for the facial prominences (e.g., FNP, MxP, MdP).

- *Line 200-205. I feel a stronger/clearer set of statements stating the conclusions about the Wnt1 vsSox10 Cre experiments would be helpful*

Thank you for this suggestion. Also taking Reviewer 3's comments into consideration, we have modified the conclusions accordingly:

"In sum, while we suspect that minor phenotypic distinctions between *Sox10:CRE* and *Wnt1:CRE* skeletons are due to timing or small CNCC subpopulations uniquely labeled by each *CRE* (Debbache et al., 2018), remarkable similarities existed. The overlap in major defects between *CRE* models further emphasizes a critical dependence on post-migratory CNCC-specific *Tfap2a* and *Tfap2b* gene dosage during skeletal differentiation and patterning events."

- *The bulk seq experiment is perhaps a bit of an outlier. It has the feel of "old data" that is looking for ahome and was added to this story. This is especially true given the pseudobulk approach taken with the scRNA Seq data. Maybe if the initial bulk data is mentioned more as a backup of the RNASeq?? It does not look like the main conclusions would be different, or any less strongly supported.*

While we acknowledge the reviewer's appreciation for the pseudobulk approach, we believe both transcriptomic experiments are relevant to this study for two reasons.

First, from a biological perspective, the E10.5 bulk RNA-seq and E11.5 scRNA-seq analyses capture two critical and distinct embryonic time points. As such, comparisons between datasets allow for the inference of gene expression changes through the course of midfacial patterning events. One such example was the increase in *Fox* and collagen genes observed more profoundly at E11.5 compared to E10.5.

Second, from a technical perspective, both datasets complement the other's weakness. For example, bulk RNA-seq provides substantially more sequencing depth than current scRNA-seq. The increased sequencing depth, and three biological replicates per condition, provides additional sensitivity for lowly expressed genes. Conversely, scRNA-seq adds cellular resolution to transcriptomic differences between controls and mutants. Moreover, because we sorted all CNCCs from controls and mutants for scRNA-seq analysis, we were able to use

© 2023. Published by The Company of Biologists under the terms of the Creative Commons Attribution License (https://creativecommons.org/licenses/by/4.0/).

known TFAP2 targets in the pharyngeal arches (Knight et al., 2004; Knight et al., 2003; Van Otterloo et al., 2018) as internal positive controls (see Table S1 for more details comparisons between the TFAP2 craniofacial datasets).
Altogether, we reasoned that both approaches provided complementary assessment of the findings and conclusions made.

- *Line 296/ Fig. 5. Should the background filter for the Hooper data set be higher than FPKM>0?*

For this analysis we chose any gene with a registered FPKM value as considered a gene with a detected transcript. While we acknowledge this likely included some 'noise' due to false calls, we felt that it ensured all potentially expressed genes were included. We note that whether FPKM cut off was set at 5 or 10, the differences between those with a peak and those without were still highly significant (adj p-value of 1.8e-09 and 7.6e-05, respectively).

- *What do you make of the fact the alx3 expression in the fish ap2 mutants seems clearly enriched? I appreciate the reduction around the periphery of the nasal placodes but the overall conclusion would seem to be different than that seen in the mouse. The genetic interaction data in the fish is clear but the molecular results shown do not necessarily make a "neat" and complementary set of experiments.*

Addressing a similar point raised by Reviewer 1 and to assess the basic conservation of this network more thoroughly, we re-analyzed current data and performed new experiments.

First, to complement our mouse TFAP2 ChIP-seq data, we performed anti-Tfap2a CUT&RUN experiments on 24-hour post fertilization (hpf) wild-type embryos and integrated these data with single- nuclei ATAC-seq dataset of CNCCs published by Fabian et al., 2022 (PMID: 35013168). Herein, we show that Tfap2a-bound regions surrounding *alx* loci (*alx1*, *alx3*, and *alx4a*) overlap with non-coding elements that are specific to the presumptive 'upper face' neural crest (new Fig. S19).

Second, to complement our mouse gene expression data, we have carried out a more thorough quantification of the *alx3* HCR signal in *tfap2a* zebrafish mutants and controls (both wild-type and *tfap2a* heterozygotes) (see new Fig. 6C, new Table S3). In doing so, we found a statistically significant decrease of *alx3* in midline domains of *tfap2a* mutants, relative to controls. However, as in our single- cell RNA-seq analysis in the mouse, not all frontonasal domains were reduced. In fact, as pointed out by the reviewer, in more lateral domains, *alx3* expression is increased upon loss of *tfap2a*.

Collectively, these data (along with the genetic interaction experiments) support a 'core network' that is utilized in both species, albeit with clear differences between mouse and fish. We speculate that this may be due to changes in the regulatory network itself (which would make sense given the differences in midface morphology between species) or may be due to the paralogs manipulated (e.g., *TFAP2A/TFAP2B*) and the paralogs monitored (e.g., *ALX1*, *ALX3*, and *ALX4*). We have modified the zebrafish sections extensively to reflect our new findings, while also acknowledging the species- specific differences.

- *Line 64 – what is meant by "encoded"?*

This initially meant "Proteins" encoded by the TFAP2 genes. We have modified the sentence accordingly to avoid confusion:

"Members of the TFAP2 transcription factor family are known central determinants of vertebrate CNCC development (de Croze et al., 2011; Knight et al., 2004; Knight et al., 2003; Li and Cornell, 2007; Rothstein and Simoes-Costa, 2020; Schorle et al., 1996; Van Otterloo et al., 2018; Zhang et al., 1996) as well as facial shape variation and evolution (Erickson et al., 2018; Naqvi et al., 2022; Prescott et al., 2015)."

- *BA1 – used in the literature along with PA1 often enough, I wonder if it is worth writing out*

© 2023. Published by The Company of Biologists under the terms of the Creative Commons Attribution License (https://creativecommons.org/licenses/by/4.0/).

branchial arch

We have changed all mentions of “branchial arch” to “pharyngeal arch” to keep consistency across our mouse and zebrafish studies.

- *Line 67 – illuminate or elucidate?*

In contrast to our first submission, which contained “illuminate” in line 97, we have altered the opening statement of the Results section as follows:

“We previously identified that simultaneous loss of *Tfap2a* and *Tfap2b* in the murine neural crest resulted in clefting of midface and jaw elements (Van Otterloo et al., 2018)”

- *Line 142 – which comprise of – word choice?*

The revised text no longer includes “comprise”.

Previous: “Previously assessed by ß-galactosidase staining with the r26r-lacZ reporter (Soriano, 1999), E9.0 and E10.0 *Tfap2*$^{\Delta/NCKO}$ embryos revealed no distinct changes in CNCC migration into the facial complex (Van Otterloo et al. 2018), which comprise of the midface-associated frontonasal prominence (FNP). . .”

Current: “Previous assessment at E9.0 and E10.0 by *r26r-lacZ*-mediated ß-galactosidase staining revealed no overt changes in CNCC migration into the facial prominences between *Tfap2*$^{\Delta/NCKO}$ and control embryos (Van Otterloo et al., 2018).”

- *Line 149 – sectional?*

We initially referred to immunofluorescence using tissue sections to examine TFAP2 protein expression. The sentence has since been changed to the following:

“In addition to *Tfap2a and Tfap2b* gene expression in pre-migratory and migratory CNCCs (Mitchell et al., 1991), we observed TFAP2A and TFAP2B protein expression in the E11.5 midface mesenchyme with overlapping patterns (Fig. 2A; Fig. S5).”

- *Methods are very well written and comprehensive. There are a number of references to van Otterloo 2018 but at least some of those in turn cite van Otterloo 2016 (e.g. skeletal preps). I would recommend avoiding the serial citations and either writing a brief summary of the methods, or citing a more original source.*

This was unintentional and we apologize for this. To our knowledge, we have carefully sifted through the Methods section and placed the correct citations.

Reviewer 3

- *Mutations in both human TFAP2 and ALX genes cause midfacial birth defects and the most significant result in the paper is the discovery of a midfacial GRN driven by Tfap2 that regulates these and other homebox-containing genes distinct from previously described regulation in the mandibular arch. However, much of the study appears to recapitulate their previous work using Tfap2 null/floxed animals (Van Otterloo et al., 2018). Comparisons between Wnt1:CRE and Sox10:CRE driven conditional knockouts are used to test if Tfap2 function is required early in CNCC migration or later in post- migratory CNCCs with regard to the midfacial clefting phenotype. However, in their previous study, the authors showed that mutants had “no distinct changes in CNCC migration into the facial complex” and concluded that “Tfap2a and Tfap2b are largely dispensable for early NCC development” (Van Otterloo et al., 2018). Thus the justification and rationale for performing the Wnt1:CRE vs. Sox10:CRE experiments is weak. In addition, the data provided and referenced by the authors are insufficient to rule out functions for Tfap2 in early midfacial CNCCs, particularly in light of the more severe clefting observed in Tfap2 null/floxed animals for*

© 2023. Published by The Company of Biologists under the terms of the Creative Commons Attribution License (https://creativecommons.org/licenses/by/4.0/).

which they provide no explanation.

We thank the reviewer for raising these observant and critical points. Also, please note, we have broken up various components of our response into several sections, as follows below.

First, regarding why subtle differences in midfacial cleft "severity" (i.e., width of the midface cleft) exist between flox/flox animals in this study and null/flox animals in Van Otterloo et al., 2018:

We suspect there are two non-mutually exclusive possibilities. First, one explanation is that the increased gap in null/flox animals may be exacerbated by the mandibular cleft as facial prominence tissue growth is an integrated process. Alternatively, knowing that TFAP2 in the facial ectoderm plays a critical role in facial patterning (Van Otterloo et al., 2022, PMID: 35333176), it is very feasible the *Tfap2a/b* heterozygosity in the ectoderm, because of the null alleles, could have also exacerbated the cleft width. Importantly, the novel flox/flox studies presented here identify that neural crest-autonomous loss of *Tfap2a/b* is sufficient to give rise to the midface cleft.

We address rationale for *Wnt1:CRE* vs *Sox10:CRE* studies, and other points, below.

- *The authors argue that their data suggest "that the midface cleft does not arise from impaired TFAP2 activity during migration" (line 164). However, the comparatively more severe phenotype of Wnt1:CRE conditional knockouts as compared to Sox10:CRE suggests that there are earlier TFAP2 contributions to midfacial clefting, or alternatively that these two CRE lines are not expressed in the same midfacial subpopulations of CNCCs at later stages (e.g. E12.5) (Debbache et al., 2018), a possibility that they acknowledge (lines 201-203) but which undermines several of their interpretations.*

We agree that the slightly larger midface cleft in *Wnt1:CRE* embryos suggests additional roles for TFAP2 during, or before, CNCC migration. However, the presence of a midface cleft in *Sox10:CR*E mutants, albeit slightly smaller, supports the 'outsized' role of TFAP2 in post-migratory neural crest.

Further, while in full transparency we acknowledged the possibility that the two *CRE* lines may label small, unique, subpopulations of neural crest, we note that published reports indicate they largely overlap (Hari et al., 2012, PMID: 22573620; Jacques-Fricke et al., 2012, PMID: 23094090). Again, the finding that *Sox10:CRE* mutants still recapitulate major midface anomalies (e.g., midfacial clefting, missing nasal bones, widened fontanelle gap, ectopic cartilage) suggests that there is a substantial overlap between the number of neural crest cells recombined by both *CRE* lines.

Thus, we reasoned that our interpretations from these studies are largely justified, suggesting that proper midfacial development depends on TFAP2 activity—in particular, within CNCCs after their migration. However, as recommended by the reviewer, we have modified the text to acknowledge the small contribution of the earlier role in midface development.

- *While CNCCs clearly populate the arches in single and double conditional KOs, as shown in their previous study, the pattern of LacZ staining in these embryos compared to controls at E10.5 would seem to suggest some CNCC migration defects. Without a more direct assay of cell migration, the authors cannot definitively conclude that there is no migratory contribution to the phenotype they observe.*

Although most of the midface is populated by ß-gal positive CNCCs, a feature never reported in the 2018 study, we acknowledge the slight changes in CNCC distribution in the midface of our lineage tracing experiments. Thus, we cannot definitively rule out the possibility that subtle CNCC premigration and/or migration defects are present. Again, we have modified the text to acknowledge the possibility of a small contribution of the earlier role in midface development.

© 2023. Published by The Company of Biologists under the terms of the Creative Commons Attribution License (https://creativecommons.org/licenses/by/4.0/).

- *It may be more appropriate to say that the postmigratory loss of TFAP2 activity has an outsized effect on midfacial clefting or that midfacial clefting is primarily due to postmigratory loss of TFAP2 activity.*

We agree with the reviewer on a more tempered interpretation of our findings. We have modified the text accordingly:

"We noted that pre-migratory loss (i.e., *Wnt1:CRE*) of *Tfap2* had a slightly more pronounced effect on cleft severity and midfacial outgrowth at later stages (Fig. 2C", D", E-H, Fig. S6D-G), consistent with earlier roles for TFAP2 in CNCC development."

- *The authors state that "further co-expression analyses indicate that Tfap2a-positive cells also express Alx3 (Fig. S16), suggesting that TFAP2A regulation of ALX gene expression is a conserved transcriptional axis in vertebrates." However, the observation that two genes are co-expressed (here, based on RNA-seq data) does not necessarily suggest that one regulates the other. Further, they attempt to test this relationship by performing in situ hybridization for Alx3 in Tfap2a mutants. However contrary to their interpretation these appear to show increased and expanded domains of Alx3 signal. Their HCR in situ methodology should allow the authors to quantify some of their expression data to support their claims.*

We agree with the reviewer that changes in *alx3* gene expression in *tfap2a* mutants were more nuanced and required further elaboration. In response to the reviewer's suggestion, we have now quantified *alx3* transcripts in the midfacial regions comprising the medial edges of nares, the roof of the mouth ('medial region' in the text), and the area under the nares that align horizontally with the roof of the mouth ('lateral region' in the text) (new Fig. 6C, new Table S3). Measurements (taken from 5 samples/genotype) confirmed a significant reduction in <u>medial</u> *alx3* transcript signal detected in *tfap2a* mutants. In contrast, measurements of lateral domains identified a significant increase in *alx3* transcripts in *tfap2a* mutants. We have modified the text accordingly to reflect these changes. Moreover, we draw attention to the fact that, although a core of this network is conserved, clear modifications between species have occurred.

We further address this with more details in response to the comment regarding the transcriptional circuit.

- *There is a general lack of quantification and rigor in many experiments. For example, referring to phenotypes as "Mildly shortened," with no measurements (lines 117-118).*

We have added additional E18.5 µCT linear measurements to compare *Wnt1:CRE* and *Sox10:CRE* embryos (new Fig S6D-G) to better conclude that *Tfap2a/b* double mutants present shortened snouts.

- *Other examples include a lack of n #s for described phenotypes, merely stating that one was "overall, more common" (line 191).*

We have added the number of embryos with the shown phenotypes in the methods, figure legends, figures, and text. See one example below:

"Finally, while the jaw phenotypes were largely shared between *CRE* models (Fig. S9A-D), *Sox10:CRE-Tfap2*^{NCKO} mutants exhibited lower penetrance for fusion of the upper and lower jaw (i.e., syngnathia) (1 of 5 skeletons) (Fig. S9E) while fusion between maxillary and jugal bones was more frequent (3 of 5 skeletons) (Fig. S9F) relative to *Wnt1:CRE-Tfap2*^{NCKO} mutants (5 of 5 skeletons for syngnathia, 0 of 5 skeletons for maxilla-jugal bone fusions) (Fig. S3E')."

- *In Fig S4 the genotypes shown in the fluorescent antibody staining panels are unclear. Each panel or set of panels should be appropriately labeled.*

© 2023. Published by The Company of Biologists under the terms of the Creative Commons Attribution License (https://creativecommons.org/licenses/by/4.0/).

While the ß-gal staining in the original Fig. S4 included a range of genotypes, the TFAP2 antibody staining was conducted on wild-type embryos, thus, we had not listed a 'genotype'—although, we appreciate that this was confusing as shown. The fluorescent images are now shown in their own figure (new Fig. S5), and 'wild-type' is indicated in the figure legend.

- *Additionally, the figure legend describes "absent TFAP2B protein expression in the nasal epithelium, in contrast to the top panel" however, there clearly appears to be staining, though with reduced intensity, in the nasal epithelium, similar to the reduced intensity in mesenchyme in the top half of the image panel. Could the authors explain this discrepancy?*

The initial labels on these two tissue sections (new Fig. S5) highlighted potential changes in TFAP2B expression throughout the nasal epithelium, along the axis sectioned. However, we agree with Reviewer 3's observations and have removed these labels to avoid confusion as this was never commented on in the manuscript text. New Fig. S5 legend:

"**Fig. S5 - TFAP2A and TFAP2B overlapping expression domains during midfacial development.**
(**A**) The top image, derived from BioRender, indicates the plane of each section that wild-type TFAP2 co-staining was performed in panels B and C as well as Fig. 2A. The bottom-left image, derived from the eMouse atlas (Armit et al., 2017), is a representative orientation of the tissue section. Boxed is the region examined in the immunofluorescent images. (**B-B", C-C"**) Co-staining of DAPI (B, C), TFAP2A (B', C'), and TFAP2B (B", C") in wild-type midface tissue in two different planes. White dashed lines indicate boundaries between mesenchyme and epithelium. Additional abbreviations: d, dorsal; ect, ectoderm; ne, nasal epithelium; np, nasal pit; v, ventral."

- *In several places the authors refer to "data not shown" and it is unclear to which data they refer (lines 287, 320-321, 327). Development also strongly discourages this usage.*

Thank you for raising this concern regarding these lines in our initial submission.

Line 287 referred to the TFAP2 antibody used for ChIP-seq. We apologize for not including the reports that suggest the sc-184 antibody recognizes TFAP2A/B/C. Schuur et al., 2001 (PMID: 11278455) demonstrated its non-specific recognition of TFAP2C via gel shift assays (their Fig 5). Jin et al., 2015 (PMID: 25966682) described sc-184 as also recognizing TFAP2B via immunofluorescence and used their own separate paralog-specific antibodies. In support with the literature, we have included additional data into supplement (*in vitro* transcription/translation assay) showing recognition of TFAP2A/B/C—in contrast to TFAP2E—with sc-184 (new Fig. S16).

Lines 320-321 referred to the lack of meaningful terms appearing in cluster 7, which contained new discrete epigenomic signatures. Thus, we have removed the "data not shown" as the pathway analysis for these coordinates is not described in the revised text.

Line 329 referred to comparison of TFAP2-bound frontonasal prominence (FNP) epigenome datasets with those of the maxillary prominence (MxP) at *Alx1/3/4* loci. Because this comparison was only shown for the mandibular prominence (MdP) datasets (Fig. 5H), we have removed the mention of "MxP" entirely.

- *For the zebrafish data the interpretation "that the transcriptional circuit identified in post-migratory CNCCs is conserved between mice and zebrafish" should be toned-down (lines 353-354).*

We appreciate the reviewer's concern of this statement as similarly raised by Reviewers 1 and 2. To test if the basic regulatory logic of TFAP2 being upstream of *ALX* gene expression can also be found in zebrafish, we integrated new CUT&RUN data with published single-nuclei ATAC-seq data generated by Fabian et al. 2022 (PMID: 35013168). Like our mouse TFAP2 ChIP-seq studies, we found that zebrafish Tfap2a is found occupying numerous *alx*-proximal

© 2023. Published by The Company of Biologists under the terms of the Creative Commons Attribution License (https://creativecommons.org/licenses/by/4.0/).

noncoding regions with open chromatin. Further, the single-nuclei resolution provides epigenomic specificity of *alx1/3/4a* gene expression towards the more rostral/upper-face CNCCs. This analysis can be found in the new Fig. S19.

Additionally, as addressed in detail in a prior comment, quantification of *alx3* HCR signal suggests that *alx3* expression patterns are sensitive to loss of *tfap2a*, including reduced expression of *alx3* at the midline, although increased *alx3* expression in lateral domains was noted. We speculate that, although a central ‘core’ of this network is conserved, the regulatory node may be fine-tuned to give rise to diverse facial structures unique to each species.

Together, we propose that the TFAP2-*ALX* regulatory node is conserved and, via evolutionary fine- tuning, serves as an entry point for species-specific midfacial development.

- *Also for the zebrafish data and new tfap2a mutant partially penetrant phenotypes, the authors describe “ectopic cartilage rods projecting dorsally from the ethmoid plate (Fig. 6J)” and while “ethmoid plate anterior dorsal ectopic cartilage” is listed in the table, no example is shown or indicated in the figure panels.*

Thank you for pointing this out. Given the low penetrance of this phenotype in *tfap2a* mutants, we chose to limit an example of it to the representative image of the double mutant (*tfap2a*$^{-/-}$;*alx3*$^{-/-}$) in Fig. 6G, where this phenotype is found at nearly 100% penetrance. We have now clarified this in the text. Also note, we have made the ‘naming’ of this phenotype consistent throughout the text and table.

- *The ChIPseq experiment utilizes an antibody that recognizes TFAP2A/B/C proteins. A comparison of ChIPseq results between Tfap2a/b null would reveal more specifically which sites are occupied by TFAP2A or B versus TFAP2C, though this is minor.*

We agree with the reviewer that this would be a terrific comparison dataset. However, given the amount of material required for ChIP-seq and the limited number of mutant embryos from any one dissection, this would be difficult to acquire, which is why we have not conducted it.

- *Supplementary figure panel labeling is confusing and inconsistent. Each figure panel should be labelled with a letter for easy and specific referral in the text and legend, similar to the main figures.*

We have added additional letter labelling to all supplemental figures.

- *With so many similar abbreviations throughout, the authors should be particularly careful in verifying that they are consistent across the main text, figure legends, and supplementary materials (see specific minor comments below). For example, Figure S7 legend/Line 944: Abbreviation “ppmx” is used for “palatal process of the maxilla” but in Figure S6 legend, “ppmx” is used for “palatal process of the premaxilla” and in Figure 3 “pppm” is used for “palatal process of the premaxilla.”*

We fully agree with this sentiment and greatly appreciate the reviewer’s attention to detail. We have tried to ensure that when used, abbreviations are consistent throughout the entirety of the manuscript.

- *Fig 4 legend “the dysregulated Alx3/4 staining in the MdP that was previously characterized (Van Otterloo et al., 2018).” I think this statement should be in the main text and not figure legend (lines 846- 847:.*

We have now removed this statement from the figure legend.

- *Line 143 “…a mandibular prominences (MdP)” to “…and mandibular prominences (MdP)”.*

© 2023. Published by The Company of Biologists under the terms of the Creative Commons Attribution License (https://creativecommons.org/licenses/by/4.0/).

- *Figure S6: There is some type of graphical error in the "2" of "Sox10:CRE-Tfap2" at top of figure.*
- *Line 941: Extra "the" in "fusion between the the jugal…"*

Thank you for catching these mistakes. We have corrected/modified these accordingly.

- *Line 186: It is difficult to appreciate the "less compacted nasal labyrinths" the authors describe in Figure 3 or S6. They appear similar to control.*

We have now included new images that show nasal labyrinths with the midfacial bones removed (new Fig. S8). Please find one excerpt of their description in the revised text below:

"Additionally, *Tfap2*^{NCKO} mutants presented . . . inflation of the posterior nasal labyrinth, and thickened nasal septum (Fig. S8A, B, arrowheads)."

- *Line 188: Specifically, what does the "both" in "both Wnt1:CRE animals" refer to?*

We apologize for the confusion. "Both *Wnt1:CRE* animals" was referring to *Tfap2a*$^{flox/flox}$;*Tfap2b*$^{flox/flox}$ and *Tfap2a*$^{null/flox}$;*Tfap2b*$^{null/flox}$ animals. We have since removed this statement and its corresponding data (previous Fig. S7) in response to Reviewer 2's suggestions to focus on the flox/flox embryos.

- *Figure S6: The authors' use of "jg?" suggests they are unsure of the identity of the labelled element though the manuscript text and Figure S6 legend unquestionably refer to the jg bone. Are or are not the authors confident in this? If not, then the text should better convey this uncertainty.*

We have removed the question mark from the "jg" label (previously Fig. S7, now Fig. S9).

- *Figure S8 and legend. SS and CS abbreviations are capitalized in the figure but lowercase in the legend.*

This figure has been removed in our resubmission, considering Reviewer 2's suggestions on focusing on flox/flox embryos.

- *Figure 6: The figure would be more appealing if panels A-D" were in the same box rather than separated A, B and C, D. Similarly, panels F-I should not be separated from their companion panels F'-I'.*

We appreciate the reviewer's comments on making Fig. 6 more appealing and easier to navigate. We have significantly modified this main figure's letter assignments for each panel (see new Fig. 6).

- *The authors should provide more information in their methods regarding HCR: e.g. the NCBI reference sequence provided to Molecular Instruments for probe design, how many probes were in the gene set, what amplifier (B1, B2, B3…).*

Details regarding HCR in the methods have now been included:

"We ordered oligos from Molecular Instruments using XM_005167111.4 *alx3* transcript and targeting the same 649-nucleotide 5' UTR sequence previously used for *in situ* hybridization (Mitchell et al., 2021). Then, we followed the manufacturer's protocol for whole-mount zebrafish embryos and larvae using the B2 amplifier (Choi et al., 2016) and probe set size of 20."

Second decision letter

© 2023. Published by The Company of Biologists under the terms of the Creative Commons Attribution License (https://creativecommons.org/licenses/by/4.0/).

MS ID#: DEVELOP/2023/202095

MS TITLE: TFAP2 paralogs regulate midfacial development in part through a conserved *ALX* genetic pathway.

AUTHORS: Timothy T. Nguyen, Jennyfer M. Mitchell, Michaela D. Kiel, Colin P. Kenny, Hong Li, Kenneth L. Jones, Robert A. Cornell, Trevor J. Williams, James T. Nichols, and Eric Van Otterloo
ARTICLE TYPE: Research Article

I am happy to tell you that your manuscript has been accepted for publication in Development, pending our standard ethics checks.

<u>Reviewer 1</u>

Advance summary and potential significance to field

The strengths noted in my initial review are maintained in the revised version, in which the authors have also satisfactorily addressed each of the comments.

Comments for the author

The strengths noted in my initial review are maintained in the revised version, in which the authors have also satisfactorily addressed each of the comments.

<u>Reviewer 2</u>

Advance summary and potential significance to field

This work explores the targets downstream of the TFAP2 family members during mid-craniofacial development. These factors are known to be important for craniofacial development. This work further determines the consequences of losing TFAP2 paralogs, both at the phenotypic level and at the deep genomic level. The key insight to the gene regulatory network is a direct role in regulating ALX factors which are also crucial for craniofacial development.

Comments for the author

The revisions satisfactorily address the comments i raised in the first review.
Furthermore, i think concerns raised by other initial reviewers are also addressed. Thank you for the opportunity to review this manuscript.

© 2023. Published by The Company of Biologists under the terms of the Creative Commons Attribution License (https://creativecommons.org/licenses/by/4.0/).
